# Prophylactic red blood cell transfusions in children and neonates with cancer: An evidence-based clinical practice guideline

**DOI:** 10.1007/s00520-024-08888-3

**Published:** 2024-11-04

**Authors:** Demi M. Kruimer, Debbie C. Stavleu, Renée L. Mulder, Leontien C. M. Kremer, Wim J. E. Tissing, Erik A. H. Loeffen, On behalf of the prophylactic red blood cell transfusion guideline panel, Dorine Bresters, Janneke H. P. Evers, Sjef P. J. van Gestel, Melanie M. Hagleitner, Katja M. J. Heitink-Pollé, Elise J. Huisman, Geert O. R. Janssens, Philip H. M. Kuijper, Maarten O. Mensink, Joppe Nijman, Jeroen G. Noordzij, Ida Ophorst, Willemijn Plieger, Judith Spijkerman, Alida F. W. van der Steeg, Marianne D. van de Wetering

**Affiliations:** 1 Princess Máxima Center for Pediatric Oncology, Utrecht, The Netherlands; 2 Department of Pediatric Oncology/Hematology, Beatrix Children’s Hospital, University Medical Center Groningen, University of Groningen, PO Box 30.001, 9700 RB Groningen, The Netherlands

**Keywords:** Pediatric oncology, Red blood cell transfusions, Clinical practice guideline

## Abstract

**Background:**

Red blood cell (RBC) transfusions play an important role in supportive care in children and neonates with cancer. However, in current clinical practice, evidence-based recommendations are lacking on when to administer prophylactic RBC transfusions. To address this gap, a clinical practice guideline (CPG) was developed to systematically review the available evidence and provide recommendations for clinicians.

**Methods:**

A systematic literature review in three databases was conducted. The GRADE methodology was used to assess, extract, and summarize the evidence. A multidisciplinary panel of 21 professionals was assembled to ensure comprehensive expertise. If there was insufficient evidence in children with cancer, additional evidence was gathered in general pediatric or adult oncology guidelines, or the panel utilized shared expert opinion to develop a comprehensive CPG. Multiple in-person meetings were conducted to discuss evidence, complete evidence-to-decision frameworks, and formulate recommendations.

**Results:**

Four studies including 203 children with all types of cancer, met the inclusion criteria. The expert panel assessed all evidence and translated it into recommendations. In total, 47 recommendations were formulated regarding RBC transfusions in children and neonates with cancer. For instance, specific thresholds for prophylactic RBC transfusions were recommended for children and neonates with cancer who have sepsis, are on ECMO, or are undergoing radiotherapy.

**Conclusion:**

This clinical practice guideline presents evidence-based recommendations regarding RBC transfusions in children and neonates with cancer. By providing these recommendations, we aim to guide clinicians and contribute to improving outcomes for children and neonates with cancer.

**Supplementary Information:**

The online version contains supplementary material available at 10.1007/s00520-024-08888-3.

## Introduction

Red blood cell (RBC) transfusions are important in the supportive care for children with cancer and those undergoing a hematopoietic stem cell transplantation (HSCT). These transfusions are often necessary due to anemia resulting from their underlying oncological disease or due to bone marrow depression during their anti-cancer treatment [1]. Blood transfusions can significantly improve the quality of life of children and neonates with cancer. However, while transfusions are generally well tolerated, they are associated with adverse short- and long-term effects (such as volume overload, transfusion reactions, and iron overload) [2, 3]. Thus, it is essential to strike a balance between unnecessary transfusions—and its adverse effects—and preventing complications caused by anemia.

Unfortunately, current clinical practice lacks evidence-based recommendations for administering RBC transfusions in children with cancer specifically. Given the frequency of these transfusions in these patients, it is crucial to critically review and assess the available evidence to develop accurate recommendations.

Therefore, our aim was to develop a clinical practice guideline (CPG) regarding RBC transfusions in children with all types of cancer in general and children with all types of cancer who are undergoing an HSCT. This CPG focuses on prophylactic RBC transfusions in children and neonates with cancer. We explicitly aimed to provide recommendations even in the absence of evidence, to establish good clinical practice and provide clinicians with a comprehensive guideline.

## Methods

### Guideline panel

A national, comprehensive multidisciplinary panel was assembled, comprising 22 professionals and a patient representative. The panel included pediatric hemato-oncologists, pediatricians, a radiotherapist, a surgeon, a patient representative, nurse specialists, a pediatric intensive care specialist, a laboratory specialist, guideline specialists, and several researchers (see Supplemental Materials S1). Members were invited on the basis of their experience and knowledge on the topic. The core group (DK, DS, RM, LK, WT, EL) provided all the preparatory documents including methodology, study details, and results. Between 2020 and 2022, multiple in-person meetings were held to rank outcomes, discuss the evidence, and formulate recommendations.

### Guideline scope

This CPG includes recommendations regarding prophylactic RBC transfusions in children with cancer aged 0–18 years receiving anti-cancer treatment with curative intent. This guideline was not intended to provide recommendations for palliative care settings or for cases of ongoing blood loss (e.g., emergency care, ongoing blood loss in gastro-intestinal tract, epistaxis). The guideline focuses on prophylactic RBC transfusions; symptoms can however influence the threshold for transfusion and clinical decision-making accordingly.

### Existing guidelines and clinical questions

Existing international guidelines on prophylactic RBC transfusions were searched (latest search February 2023: National Institute for Health and Care Excellence (NICE), Guidelines International Network (GIN), American Society of Clinical Oncology (ASCO), international Pediatric Oncology Group (iPOG), and Cancer Guideline Database) and evaluated for the applicability and completeness of these guidelines. Considering the absence of an applicable evidence-based guideline for children with cancer, clinical questions were formulated by the core group. An overview of the clinical questions is shown in the Supplemental Materials S2.

### Search strategy and selection criteria

An extensive systematic literature search (shown in Supplemental Materials S3) was performed in collaboration with a medical librarian. We searched electronic databases PubMed, Embase, and Cochrane CENTRAL.

In- and exclusion criteria were predefined by the core group. Importantly, all children and neonates with all types of cancer aged 0 to 18 years were included. Studies were included if groups with different thresholds for RBC transfusions were compared. We only included controlled studies, applying a two-step approach by first including RCTs, but in case of insufficient or inconclusive evidence, we included other controlled studies. It was agreed upon that when there were not enough studies identified, we would extrapolate from evidence-based guidelines in other pediatric patient populations (e.g., benign hematology or cardiology) or guidelines in adult oncology patients (applicability depending on clinical question).

### Primary evidence selection and quality assessment

Study identification was performed by title and abstract screening, followed by full text assessment, independently by two reviewers (DK, DS). Discrepancies were resolved by consensus.

Detailed information from each eligible study was extracted into evidence tables. The methodological quality of each single study was assessed and scored on risk of bias. For RCTs, the risk of bias tool v2 from the Cochrane handbook [4] was used. For non-RCT studies, we combined the risk of bias criteria for observational studies, as described in the Handbook of the International Guideline Harmonization Group [5], with specific aspects of the Cochrane RCT tool [4]. By combining these tools, we aimed to have the best possible tool to assess the risk of bias in our types of studies. These risk of bias assessment criteria for non-RCT studies and the risk of bias results are shown in the Supplemental Materials S4.

All the evidence was collected in summary of findings tables. Per outcome, the quality of the total body of evidence was assessed by the Grading of Recommendations Assessment, Development and Evaluation (GRADE) approach [6]. Data-extraction, risk of bias assessment, and GRADE assessment were independently performed by two reviewers (DK, DS). Discrepancies were resolved by consensus.

### Additional evidence selection and quality assessment

In anticipation of a lack of studies in childhood cancer patients, we searched for additional evidence. Guidelines on RBC transfusions in children without cancer or adults with cancer were searched in PubMed, Joint United Kingdom Blood Transfusion Services Professional Advisory Committee (JPAC), NICE, GIN, ASCO, iPOG, and Dutch Federation of Medical Specialists (FMS). The quality of the guidelines was assessed according to the AGREE II [7] method. A guideline was eligible for inclusion if the AGREE II-score was 4 or higher (Supplemental Materials S5). The included single studies in those guidelines served as the evidence base for extrapolation. In addition, in case of lack of evidence, recommendations from high-quality guidelines are adopted.

### Translating evidence into recommendations using the evidence-to-decision framework

The GRADE evidence-to-decision framework was used to translate evidence into recommendations [6]. Within this framework, for every clinical question, the benefits and harms, resource use, equity, acceptability, and feasibility were discussed, and recommendations were formulated by the guideline panel. If no studies were identified, we carefully considered expert consensus (expert opinion). All expert opinion recommendations can be interpreted on the level of “weak recommendations,” as the panel felt there were no expert opinion recommendations that should be labeled as “strong recommendation.” Final recommendations were unanimously supported by all panel members.

The GRADE terminology for evidence-based guidelines was used, such as “we suggest” or “we recommend” [6]. For the expert-based recommendations, the terminology from a recent paper published by the international Pediatric Oncology Guidelines in supportive care (iPOG) network [8] was used. The wording “we believe” was used to emphasize that these recommendations are based on expert opinion and group consensus. A color-coding system was used to improve understandability and to emphasize the strength of the recommendations [9].

## Results

In total, 8132 unique citations were identified in initial literature search (September 2019) and two update searches (latest: February 2023), see flowchart 1.

**Flowchart 1:**
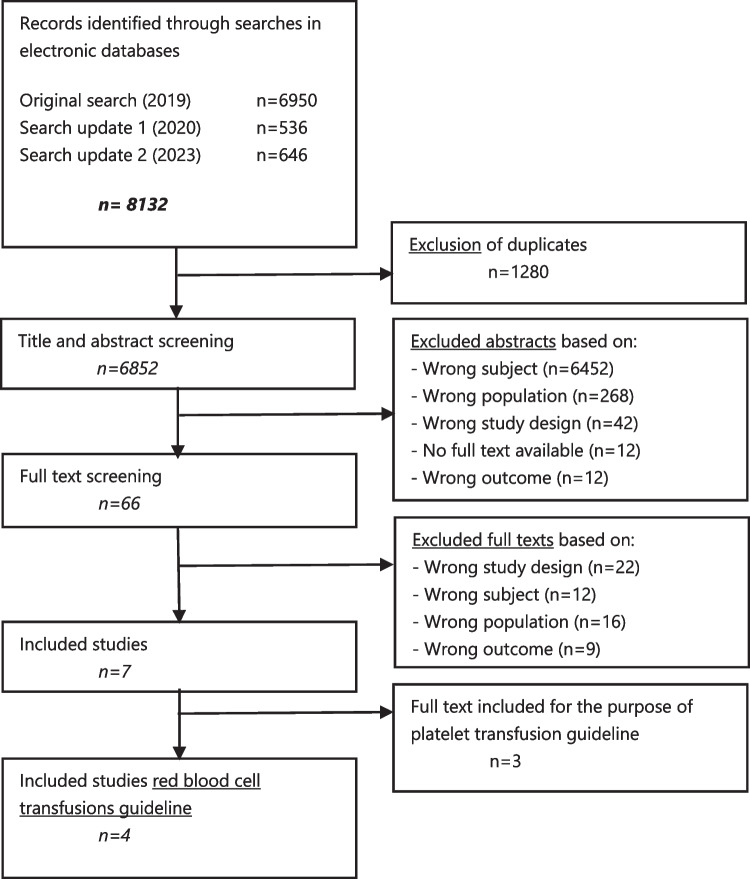
Flowchart of the inclusion and exclusion process (including the interim updates)

Four primary studies (3 RCTs, 1 pre-post trial) were included with a total number of 203 participants (see Fig. 1 in Supplemental Materials S6). All primary study characteristics and conclusions of evidence are shown in Supplemental Materials S6, including the inclusion and exclusion process. Moreover, seven (non-childhood cancer) guidelines were included with a total of 43 different single studies. An overview of the included studies, the conclusions of evidence, the evidence tables, and the GRADE assessments can be found in the Supplemental Materials S7. An overview of RBC transfusion recommendations for children and neonates with cancer is presented in Supplemental Materials S8. Within the overview of all recommendations, a color-coding system was used to improve understandability and to emphasize the strength of the recommendations. Below, all recommendations and their evidence-to-decision processes are discussed per subject. Given the number of recommendations and the extent of the supporting materials, only conclusions and important considerations of the guideline panel are shown. Full details, including the evidence to decision frameworks, are shown in the Supplemental Materials S9. The results section is divided into the different circumstances in which we recommend a prophylactic RBC transfusion. An overview of the recommendations for scientific research is included in Supplemental Materials S10.

**Fig. 1:**
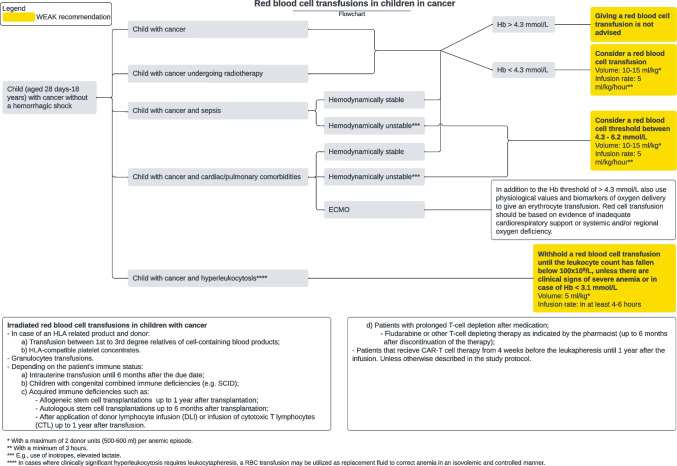
Flowchart of RBC transfusion recommendations for children with cancer

**Fig. 2:**
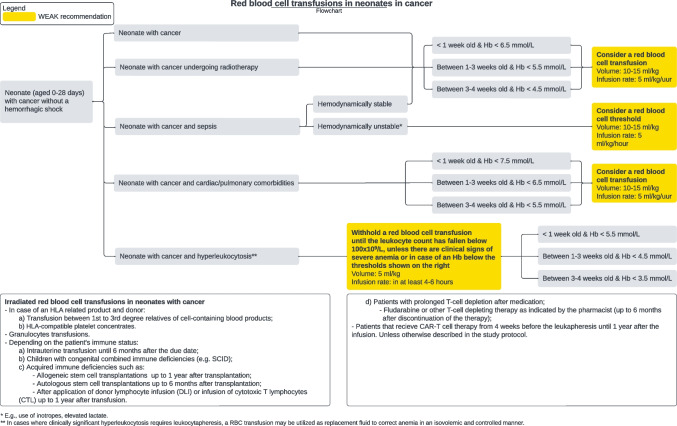
Flowchart of RBC transfusion recommendations for neonates with cancer

The recommendations on RBC transfusions for children and neonates with cancer are visualized below (Figs. 1 and 2). These flowcharts are also offered separately with measurements of Hb in g/dL.

### Prophylactic red blood cell transfusion in general

#### Prophylactic red blood cell transfusion in children with cancer

##### Recommendation 1.1.1.

We suggest a hemoglobin (Hb) threshold of 4.3 mmol/L for RBC transfusion in children with cancer. (weak recommendation, very low quality evidence).

##### Recommendation 1.1.2.

We suggest against an Hb threshold of 3.7 mmol/L for RBC transfusion in children with cancer. (weak recommendation, very low quality evidence).

##### Recommendation 1.1.3.

We recommend against an Hb threshold of 3.1 mmol/L or lower for RBC transfusion in children with cancer. (strong recommendation, very low quality evidence).

##### Recommendation 1.1.4.

We suggest against an Hb threshold greater than 4.3 mmol/L for RBC transfusion in children with cancer. (weak recommendation, very low quality evidence).

##### Evidence to decision

The comparison of an Hb threshold of 4.3 mmol/L to an Hb threshold greater than 4.3 mmol/L involved two pediatric oncology studies, one pediatric non-cancer study, and five adult non-cancer studies. Apart from significantly lower costs, there was no significant increased risk for mortality, morbidity, and transfusion-related complications with a threshold Hb of 4.3 mmol/L in comparison to an Hb threshold greater than 4.3 mmol/L in children with cancer (VERY LOW quality of evidence) [1, 10]. From the guidelines that included single studies with children in general and adults, one adult study reported significantly higher mortality in the group with an Hb < 4.3 mmol/L in comparison to an Hb > 4.3 mmol/L in group [11]. Another adult study reported significantly lower mortality in the group with an Hb < 4.3 mmol/L in comparison to an Hb > 4.3 mmol/L in group [12], while six other pediatric studies with cancer and adult studies reported no significant difference in mortality [1, 10, 12–15]. Based on the available evidence, the panel concluded that there is likely no increased mortality risk. Additionally, two studies demonstrated fewer infections with an Hb threshold of 4.3 mmol/L compared to an Hb threshold greater than 4.3 mmol/L [12, 16]. Furthermore, there was no significant increase in quality of life with a higher Hb threshold than 4.3 mmol/L [12]. Considering these findings, the guideline panel determined that the benefits of maintaining an Hb threshold of 4.3 mmol/L compared to an Hb threshold greater than 4.3 mmol/L are likely substantial. Therefore, we suggest an Hb threshold of 4.3 mmol/L in children with cancer. Moreover, no other study reported significant increase in benefits or harms from a higher Hb threshold, such as 5.0 mmol/L [1, 12, 14, 16–19]. Also, the guideline panel considered the potential risks of iron overload and increased costs associated with a higher Hb threshold, and therefore, we suggest against adopting an Hb threshold greater than 4.3 mmol/L.

Regarding the comparison of an Hb threshold of 3.7 mmol/L to an Hb threshold greater than 3.7 mmol/L, no pediatric oncology studies were found. However, there were two adult non-cancer studies identified from the included guidelines. Pooled results indicated a significantly increased mortality risk in adult patients with an Hb threshold of 3.7 mmol/L in comparison to an Hb threshold greater than 3.7 mmol/L [11, 13]. Similar to the previous comparison, no studies reported any potential benefit from an Hb threshold of 3.7 mmol/L. Therefore, we suggest against an Hb threshold of 3.7 mmol/L.

Regarding the comparison of an Hb threshold of 3.1 mmol/L to an Hb threshold greater than 3.1 mmol/L, no pediatric oncology studies were found. However, there were three adult non-cancer studies and one pediatric non-cancer study identified from the included guidelines. These studies consistently reported significantly higher mortality rates in hospitalized adults and children with an Hb of 3.1 mmol/L [11, 13, 20, 21]. Despite the low level of evidence, which is mainly derived from adult studies, the guideline panel strongly advised against offering this option due to the higher mortality rates.

#### Prophylactic red blood cell transfusion in neonates with cancer

##### Recommendation 1.2.1.

We suggest an Hb threshold of 6.5 mmol/L for RBC transfusion in neonates with cancer when they are less than 1 week old.

(weak recommendation, very low quality evidence).

##### Recommendation 1.2.2.

We suggest an Hb threshold of 5.5 mmol/L for RBC transfusion in neonates with cancer when they are between 1 and 3 weeks old.

(weak recommendation, very low quality evidence).

##### Recommendation 1.2.3.

We suggest an Hb threshold of 4.5 mmol/L for RBC transfusion in neonates with cancer when they are between 3 and 4 weeks old.

(weak recommendation, very low quality evidence).

##### Evidence to decision

The incidence of cancer in neonates is exceedingly low. Despite this, it is crucial to provide recommendations for this specific patient group. Unfortunately, no pediatric oncology studies were identified to inform the guideline panel’s decision. However, the Dutch Association of Medical Specialists (FMS) [22] developed a high-quality guideline addressing this matter, receiving an AGREE II-score of 6 out of 7. They provided recommendations primarily based on studies conducted in very low birth-weight infants (birth weight of 1500 g or less). Although evidence specific to full-term and late-premature neonates (gestational age ≥ 32 weeks) is lacking, the FMS has adopted these thresholds for neonates in general. Considering the lack of evidence, the guideline panel decided to adopt the recommendations regarding neonates with cancer from the guideline of the FMS (2019).

### Prophylactic red blood cell transfusion—sepsis

#### Prophylactic red blood cell transfusion in children with cancer during sepsis

##### Recommendation 2.1.1.

We suggest an Hb threshold of 4.3 mmol/L for RBC transfusion in children with cancer during sepsis who are hemodynamically stable.

(weak recommendation, very low quality evidence).

##### Recommendation 2.1.2.

We believe that for hemodynamically unstable children with cancer during sepsis and evidence of oxygen deficiency (e.g., use of inotropes, elevated lactate), an Hb threshold that ranges between 4.3 and 6.2 mmol/L should be considered.

(expert opinion).

##### Evidence to decision

Regarding children with cancer during sepsis who are hemodynamically stable, one pediatric non-cancer study and one adult non-cancer study were identified. Based on this limited evidence, there is no suggestions that there is an increased risk for mortality or morbidity with an Hb threshold of 4.3 mmol/L in comparison to an Hb threshold greater than 4.3 mmol/L in children and adults with sepsis who are clinically stable [17, 23]. Furthermore, no studies reported any significant potential benefit from an Hb threshold greater than 4.3 mmol/L [17]. Therefore, we suggest an Hb threshold of 4.3 mmol/L in children with cancer during sepsis who are hemodynamically stable. However, in hemodynamically unstable children with cancer during sepsis and evidence of oxygen deficiency (e.g., use of inotropes, elevated lactate), it is suggested to consider an Hb threshold ranging between 4.3 mmol/L and 6.2 mmol/L as part of a comprehensive approach to improve oxygen delivery for children with unstable non hemorrhagic shock and evidence of oxygen debt (WEAK recommendation) [24].

#### Prophylactic red blood cell transfusion in neonates with cancer during sepsis

##### Recommendation 2.2.1.

We suggest an Hb threshold of 6.5 mmol/L for RBC transfusion in neonates with cancer during sepsis when they are less than 1 week old.

(weak recommendation, very low quality evidence).

##### Recommendation 2.2.2.

We suggest an Hb threshold of 5.5 mmol/L for RBC transfusion in neonates with cancer during sepsis when they are between 1 and 3 weeks old.

(weak recommendation, very low quality evidence).

##### Recommendation 2.2.3.

We suggest an Hb threshold of 4.5 mmol/L for RBC transfusion in neonates with cancer during sepsis when they are between 3 and 4 weeks old.

(weak recommendation, very low quality evidence).

##### Evidence to decision

There were no studies found on neonates with cancer during sepsis. There was no suggestion for an increased risk for mortality and morbidity in hemodynamically stable children and adults with sepsis with an Hb threshold of 4.3 mmol/L in comparison to an Hb threshold greater than 4.3 mmol/L (“Prophylactic red blood cell transfusion in children with cancer during sepsis” section) [17, 23, 24]. Therefore, we concluded that children with sepsis do not derive additional benefits from a higher Hb threshold compared to children without sepsis. Based on these findings, and the absence of direct evidence in neonates with sepsis, the guideline panel determined that the recommendations for neonates with cancer can be applied to neonates with cancer during sepsis as well (“Prophylactic red blood cell transfusion in neonates with cancer” section).

### Prophylactic red blood cell transfusion—radiotherapy

#### Prophylactic red blood cell transfusion in children who undergo radiotherapy

##### Recommendation 3.1.1.

We believe an Hb threshold of 4.3 mmol/L for RBC transfusion should be maintained in children with cancer who undergo radiotherapy.

(expert opinion).

##### Evidence to decision

No studies specifically addressing children with cancer undergoing radiotherapy were identified. Several other studies including adults with cancer concluded that there was no improvement in outcomes with an Hb threshold greater than 4.3 mmol/L [25–28]. Therefore, we suggest an Hb threshold of 4.3 mmol/L for RBC transfusion in children with cancer who undergo radiotherapy.

#### Prophylactic red blood cell transfusion in neonates who undergo radiotherapy

##### Recommendation 3.2.1.

We believe an Hb threshold of 6.5 mmol/L for RBC transfusion should be maintained in neonates with cancer who undergo radiotherapy when they are less than 1 week old.

(expert opinion).

##### Recommendation 3.2.2.

We believe an Hb threshold for RBC transfusion of 5.5 mmol/L should be maintained in neonates with cancer who undergo radiotherapy when they are between 1 and 3 weeks old.

(expert opinion).

##### Recommendation 3.2.3.

We believe an Hb threshold for RBC transfusion of 4.5 mmol/L should be maintained in neonates with cancer who undergo radiotherapy when they are between 3 and 4 weeks old.

(expert opinion).

##### Evidence to decision

No specific studies in neonates with cancer were identified. For the considerations of the recommendations we refer to “Prophylactic red blood cell transfusion in children who undergo radiotherapy” section.

### Prophylactic red blood cell transfusion—cardiac and pulmonary comorbidities

#### Prophylactic red blood cell transfusion in children with cancer with cardiac and/or pulmonary comorbidities

##### Recommendation 4.1.1.

We suggest an Hb threshold of 4.3 mmol/L for RBC transfusion in children with cancer and cardiac and/or pulmonary comorbidities.

(weak recommendation, very low quality evidence).

##### Recommendation 4.1.2.

We believe that in case of a hemodynamically unstable child with cancer and pulmonary and/or cardiac comorbidities (e.g., use of inotropes, elevated lactate), a higher Hb threshold can be considered.

(weak recommendation, very low quality evidence).

##### Recommendation 4.1.3.

For children on ECMO:

- In critically ill children on ECMO, there is insufficient evidence to recommend a specific RBC transfusion decision-making strategy using physiologic-based metrics and biomarkers.
- In critically ill children on ECMO, we believe in using physiologic metrics and biomarkers of oxygen delivery in addition to Hb concentration to guide RBC transfusion. Administration of a RBC transfusion should be based on evidence of inadequate cardiorespiratory support or decreased systemic and/or regional oxygen delivery.

(expert opinion).

##### Evidence to decision

No pediatric oncology studies were identified. Two pediatric non-cancer studies and one adult non-cancer study were identified. The evidence gathered from these studies indicated that there is no increased risk for mortality, morbidity, and hospital admission with an Hb threshold of 4.3 mmol/L compared to an Hb threshold greater than 4.3 mmol/L in children and adults with cardiac and pulmonary comorbidities [11, 17, 29]. Studies comparing higher restrictive Hb thresholds (such as 5.0 mmol/L or 5.6 mmol/L) also did not report significant better outcomes regarding mortality, morbidity, quality of life, and admission to hospital [19, 30, 31].  Therefore, the guideline panel decided to suggest an Hb threshold of 4.3 mmol/L in children with cancer and cardiac and pulmonary comorbidities. For hemodynamically unstable children with cancer and pulmonary and/or cardiac comorbidities, such as those requiring inotropes or exhibiting elevated lactate levels, an Hb threshold ranging between 4.3 and 6.2 mmol/L is considered. Regarding children on extracorporeal membrane oxygenation (ECMO), the guideline panel decided to adopt the recommendations stated above from the Valentine (2018) guideline [32], AGREE-II score 5 out of 7.

#### Prophylactic red blood cell transfusion in neonates with cancer with cardiac and/or pulmonary comorbidities

##### Recommendation 4.2.1.

We suggest an Hb threshold of 7.5 mmol/L for RBC transfusion in neonates with cancer and cardiac and pulmonary comorbidities when they are less than 1 week old.

(expert opinion).

##### Recommendation 4.2.2.

We suggest an Hb threshold of 6.5 mmol/L for RBC transfusion in neonates with cancer and cardiac and pulmonary comorbidities when they are between 2 and 3 weeks old.

(expert opinion).

##### Recommendation 4.2.3.

We suggest an Hb threshold of 5.5 mmol/L for RBC transfusion in neonates with cancer and cardiac and pulmonary comorbidities when they are between 3 and 4 weeks old.

(expert opinion).

##### Evidence to decision

No pediatric oncology studies addressing this clinical question were found. However, the Dutch Association of Medical Specialists (FMS) [22] developed recommendations primarily based on studies conducted in very low birth-weight infants (birth weight of 1500 g or less) who required respiratory support. Although evidence specific to full-term and late-premature neonates (gestational age ≥ 32 weeks) is lacking, the FMS has adopted these thresholds for neonates requiring respiratory support. Taking this into account, the guideline panel decided to adopt the recommendations regarding neonates with cancer and pulmonary and/or cardiac comorbidities from the guideline of the FMS (2019).

### Prophylactic red blood cell transfusion—hyperleukocytosis

#### Prophylactic red blood cell transfusion in children with cancer during hyperleukocytosis

##### Recommendation 5.1.1.

In children with cancer and hyperleukocytosis, we believe that a RBC transfusion should be given with restraint until the number of leukocytes has fallen below 100 × 109 /L or in the presence of clinical symptoms of hyperleukocytosis.

##### Recommendation 5.1.2.

In children with cancer and hyperleukocytosis, we believe that a RBC transfusion should be given with restraint, unless there are severe clinical signs of anemia or in case of an Hb below 3.1 mmol/L.

##### Recommendation 5.1.3.

If needed, transfuse with a maximum of 5 ml/kg/4–6 h.

(expert opinion).

##### Evidence to decision

No specific studies addressing this topic were identified. However, a study focusing on the management of hyperleukocytosis in children and adults with cancer provided relevant information. According to this study, the use of RBC transfusions in such cases should generally be avoided due to the potential increase in blood viscosity and the associated risk of leukostasis development or exacerbation, unless the patient exhibits symptoms of anemia [33]. The guideline panel decided to take this into consideration in order to make a recommendation based on expert opinion. However, in cases where clinically significant hyperleukocytosis requires leukocytapheresis, a RBC transfusion may be utilized as replacement fluid to correct anemia in an isovolemic and controlled manner [34].

#### Prophylactic red blood cell transfusion in children and neonates with cancer during hyperleukocytosis

##### Recommendation 5.2.1.

In neonates with cancer and hyperleukocytosis, we believe that a RBC transfusion should be given with restraint until the number of leukocytes has fallen below 100 × 109 /L or in the presence of clinical symptoms of hyperleukocytosis.

##### Recommendation 5.2.2.

In neonates with cancer and hyperleukocytosis, we believe that a RBC transfusion should be given with restraint unless there are severe clinical signs of anemia or in case of an Hb below 5.5 mmol/L in neonates with cancer when they are less than 1 week old.

##### Recommendation 5.2.3.

In neonates with cancer and hyperleukocytosis, we believe that a RBC transfusion should be given with restraint unless there are severe clinical signs of anemia or in case of an Hb below 4.5 mmol/L for RBC transfusion in neonates with cancer when they are between 1 and 3 weeks old.

##### Recommendation 5.2.4.

In neonates with cancer and hyperleukocytosis, we believe that a RBC transfusion should be given with restraint unless there are severe clinical signs of anemia or in case of an Hb below 3.5 mmol/L for RBC transfusion in neonates with cancer when they are between 3 and 4 weeks old.

##### Recommendation 5.2.5.

If needed, transfuse with a maximum of 5 ml/kg/4–6 h.

(expert opinion).

##### Evidence to decision

No specific studies addressing this topic were identified. For the considerations of the recommendations we refer to “Prophylactic red blood cell transfusion in children with cancer during hyperleukocytosis” section. The RBC thresholds were based on expert opinions.

### Irradiated red blood cell transfusions

#### Irradiated red blood cell transfusions in children and neonates with cancer

##### Recommendation 6.1.1.

We believe that irradiated blood products should be used in case of an HLA-related product and donor:

- Transfusion between 1^st^ and 3^rd^ degree relatives of cell-containing blood products
  (expert opinion).

##### Recommendation 6.1.2.

We believe that irradiated blood products should be used in case of granulocyte transfusions.

(expert opinion).

##### Recommendation 6.1.3.

We believe that irradiated blood products should be used depending on the patient’s immune status:

- During intrauterine transfusions until 6 months after the due date;
- Children with congenital combined immune deficiencies (e.g., SCID); and
- Acquired immune deficiencies such as:
  Allogeneic stem cell transplantations up to 1 year after transplantation;
  Autologous stem cell transplantations up to 6 months after transplantation; and
  After application of donor lymphocyte infusion (DLI) or infusion of cytotoxic T lymphocytes (CTL) up to 1 year after transfusion.

(expert opinion).

##### Recommendation 6.1.4.

We believe that irradiated blood products should be used in case of patients with prolonged T cell depletion after medication:

- Fludarabine or other T cell depleting therapy as indicated by the pharmacist (up to 6 months after discontinuation of the therapy)

##### Recommendation 6.1.5.

We believe that irradiated blood products should be used in case of patients that receive CAR-T cell therapy from 4 weeks before the leukapheresis until 1 year after the infusion. Unless otherwise described in the study protocol.

(expert opinion).

##### Evidence to decision

There were no pediatric oncology studies identified. However, the Dutch Association of Medical Specialists (FMS) (21) developed a high-quality guideline addressing this matter. The guideline drew its recommendations from a study of Kopolovic (2015) [35] and a survey among hemovigilance organizations worldwide. Considering the lack of evidence, the guideline panel decided to adopt the recommendations regarding irradiated blood products from the guideline of the FMS (2019) [22]. The guideline panel added the indication for the use of CAR-T cells, based on the recommendations in the current study protocol (the pharmaceutical company that creates the CAR-T cells prescribed this period of irradiated blood products in a research context).

### Low- or high-volume red blood cell transfusions

#### Low- or high-volume red blood cell transfusions in children with cancer

##### Recommendation 7.1.1.

We suggest a transfusion volume of 10–15 ml/kg in children with cancer.

(weak recommendation, very low quality evidence).

##### Recommendation 7.1.2.

We suggest against a transfusion volume of 20 ml/kg or higher in children with cancer.

(weak recommendation, very low quality evidence).

##### Recommendation 7.1.3.

We suggest a transfusion volume with a maximum of 2 donor units (between 500 and 600 ml) per anemic episode.

(expert opinion).

##### Evidence to decision

No pediatric oncology studies were identified. In comparing a RBC transfusion volume of 10 ml/kg to a volume higher than 10 ml/kg, no studies including children were identified. However, one study involving neonates without cancer was identified. The limited evidence available suggests that there is no significant increase in morbidity associated with a transfusion volume of 10 ml/kg compared to a volume higher than 10 ml/kg [36]. Regarding the comparison of a volume of 15 ml/kg to a volume higher than 15 ml/kg, again, no studies including children were identified. However, two studies with neonates were identified. The available evidence suggests that there is no significant increase in mortality or morbidity associated with a transfusion volume of 15 ml/kg compared to a volume higher than 15 ml/kg [37, 38]. One study involving children without cancer compared a RBC transfusion volume of 20 ml/kg to a volume higher than 20 ml/kg [39]. The limited evidence available suggested that there is no significant difference in terms of mortality or morbidity when comparing a transfusion volume of 20 ml/kg to a volume higher than 20 ml/kg [39]. Additionally, the expert panel considered that a lower transfusion volume leads to reduced risk of volume overload and deemed this option as probably acceptable for all stakeholders. Therefore, we suggest in favor of a transfusion volume of 10–15 ml/kg, and suggest against the use of a volume of 20 ml/kg. The expert panel advises transfusing with a maximum of 2 donor units per anemic episode, which corresponds to a volume between 500 and 600 ml, based on shared expert opinion.

#### Low or high-volume red blood cell transfusions in neonates with cancer

##### Recommendation 7.2.1.

We suggest a transfusion volume of 10–15 ml/kg in neonates with cancer.

(weak recommendation, very low quality evidence).

##### Recommendation 7.2.2.

We suggest against a transfusion volume of 20 ml/kg or higher in neonates with cancer.

(weak recommendation, very low quality evidence).

##### Evidence to decision

For the considerations of the recommendations, we refer to “Low or high-volume RBC transfusion in children with cancer” section.

### Infusion rates of red blood cell transfusions

#### Infusion rates of red blood cell transfusions in children with cancer

##### Recommendation 8.1.1.

We believe that the infusion rate of a RBC transfusion should be 5 ml/kg/h in children with cancer, with a minimum of 3 h.

(expert opinion).

##### Evidence to decision

There are no studies regarding infusion rates. However, JPAC [40] has provided a recommendation for an infusion rate of 5 ml/kg/h in children, based on consensus, AGREE II-score of 4 out of 7. The guideline panel decided to adopt this recommendation, but added to the advice that a transfusion should take at least 3 h, based on expert-opinions.

#### Infusion rates of red blood cell transfusions in neonates with cancer

##### Recommendation 8.2.1.

We believe that the infusion rate of a RBC transfusion should be 5 ml/kg/h in neonates with cancer.

(expert opinion).

##### Evidence to decision

No specific studies were identified regarding infusion rates. However, the Dutch Association of Medical Specialists [22] has provided a recommendation for an infusion rate of 5 ml/kg/h in neonates, based on consensus. The guideline panel decided to adopt this recommendation.

## Discussion

This clinical practice guideline comprises recommendations, in line with the GRADE methodology [6], regarding prophylactic RBC transfusions in children and neonates with cancer and has the potential to provide valuable guidance for clinicians in daily practice and contribute to improving quality of life for children and neonates with cancer worldwide.

The most notable limitation of this CPG is the substantial lack of evidence regarding appropriate thresholds for prophylactic RBC transfusions in children and neonates with cancer. To address this limitation, we conducted comprehensive and extensive literature searches, including exploration of RBC transfusion guidelines for children without cancer and (young) adults with cancer. Unfortunately, the yield of relevant evidence was still remarkably low. However, the consensus among the guideline panel was unanimous in their determination to come up with recommendations even in the absence of adequate evidence from the literature. This was deemed essential, as healthcare providers in daily practice rely on practice guidelines to guide decision-making regarding transfusions in their patients. Consequently, the guideline panel incorporated recommendations from existing high-quality guidelines regarding RBC transfusions for adults with cancer and children in general in order to formulate recommendations based on the best available evidence. When such guidelines were unavailable, recommendations were constructed through expert consensus. We firmly believe that the incorporation of expert opinions serves as a valuable asset in enhancing clinical practice and should find more frequent implementation in the development of guidelines when evidence gaps exist.^1^ Nevertheless, with prophylactic RBC transfusions being administered so frequently in children and neonates with cancer and the potential serious consequences of anemia, it is abundantly clear that further research in this field is imperative.

A second limitation of our guideline is the composition of the guideline panel, which consisted of experts from a national level. While this panel provided valuable insights and expertise, it is important to consider the applicability of this guideline to local contexts. However, we have provided extensive supplemental materials and evidence-to-decision frameworks that allow clinicians to assess the relevance and applicability of the guidelines to their specific settings. This approach empowers clinicians in other countries to make informed decisions based on the available evidence and adapt the recommendations as needed for their local context.

Implementation of this evidence-based guideline holds promise for enhancing the quality of life in children and neonates with cancer. With these evidence- and expert-based recommendations, we have endeavored to provide comprehensive and practical guidance. To ensure transparency, we have meticulously documented all the considerations in the evidence-to-decision frameworks. The inclusion of evidence-to-decision frameworks in this guideline provides clinicians with a valuable tool to assess the individual benefits and harms associated with different treatment options for each child and are making the decision-making process transparent. We sincerely hope that this guideline serves as a valuable tool in balancing the benefits and risks, promoting cautiousness and restrictiveness where appropriate.

In conclusion, through the effective implementation of the recommendations outlined in this CPG, the guideline panel aims to improve care provided to children and neonates with cancer and contribute to enhancing their quality of life. These recommendations hold significant importance in current clinical practice, and we hope that the lack of evidence in this area will serve as a stimulus for further research efforts. We are currently developing indicators to monitor the impact of this guideline and to facilitate continuous evaluation and improvement of care in this field.

## Supplementary information

Below is the link to the electronic supplementary material.

S1: Members of the guideline panel Supplementary file1 (DOCX 9 KB)

S2: Overview of the clinical questions Supplementary file2 (DOCX 7 KB)

S3: Systematic literature search Supplementary file3 (DOCX 10 KB)

S4: Risk of Bias-tool & results Supplementary file4 (DOCX 103 KB)

S5: Description of the additional guidelines (including AGREE II-scores) Supplementary file5 (DOCX 19.2 KB)

S6: Study characteristics of the primary studies Supplementary file6 (DOCX 755 KB)

S7: Overview of the included studies, conclusions of evidence, the evidence tables and the GRADE assessments Supplementary file7 (DOCX 81.6 KB)

S8: Overview of the recommendations Supplementary file8 (DOCX 19 KB)

S9: Supporting materials Supplementary file9 (DOCX 753 KB)

S10: Gaps in research Supplementary file10 (DOCX 7 KB)

S11: Flowcharts of RBC transfusion recommendations, in g/dL Supplementary file11 (DOCX 455 KB)

## Data Availability

No datasets were generated or analyzed during the current study.

## Footnotes

1 Important note: The content of this guideline includes expert opinions and recommendations related to blood transfusions. It is crucial to understand that these expert opinions are guiding principles and are intended to serve as a reference for medical decision-making. They do not constitute legally binding regulations and are not meant to compel medical professionals to deviate from their professional judgment in any way. Medical professionals retain the right to, in accordance with their professional assessment, deviate from the recommendations outlined herein. The use of this guideline in legal proceedings should be approached with caution, as it serves as a guide for medical practice and does not impose absolute standards. Always consult your own legal counsel or medical professionals for specific legal or medical issues.
