# Supplementary material for: Prophylactic red blood cell transfusions in children and neonates with cancer: An evidence-based clinical practice guideline
Source: Support Care Cancer. 2024 Nov 4;32(11):766. doi: 10.1007/s00520-024-08888-3 (PMC11534970; doi:10.1007/s00520-024-08888-3)
Supplement: Supplementary file 1 — Supplementary file1 (DOCX 9 KB) [file 520_2024_8888_MOESM1_ESM.docx]

**Supplemental Materials
Supplemental Materials S1: Members of the guideline panel**

Core group

| **Core group** | **Function** | **Additional functions** | **Reported interests** | **Action taken** |
| --- | --- | --- | --- | --- |
| Kruimer | Physician-researcher pediatric oncology, Princess Máxima Center, Utrecht | Clinical doctor in Pediatric Oncology | None | None |
| Loeffen | Postdoc researcher pediatric oncology, Princess Máxima Center, Utrecht and Beatrix Children’s Hospital, University Medical Center Groningen | Pediatric resident, Epidemiologist B | None | None |
| Stavleu | PhD candidate pediatric oncology, Princess Máxima Center, Utrecht and Beatrix Children’s Hospital (University Medical Center Groningen) | None | None | None |
| Mulder | Postdoc researcher, guideline developer, methodologist, Princess Máxima Center, Utrecht | None | None | None |
| Tissing | Pediatric oncologist, professor of Supportive Care, Princess Máxima Center, Utrecht and Beatrix Children’s Hospital (University Medical Center Groningen) | Grants via KiKa, KWF, ZonMW in the context of activities of the Supportive Care Research group | None | None |
| Kremer | Pediatrician, Professor of Late Effects in Pediatric Oncology, Princess Máxima Center, Utrecht and Professor of Proper Care, Amsterdam UMC, Amsterdam | Adviser of the Palliative Care Knowledge Center, grants via KiKa, KWF, ZonMW in the context of activities of the Late Effects Research group | None | None |

Guideline panel

| **Guideline panel member** | **Function** | **Additional functions** | **Reported interests** | **Action taken** |
| --- | --- | --- | --- | --- |
| Bresters | Pediatric oncologist, Princess Máxima Center, Utrecht | None | None | None |
| Evers | Nurse practitioner, Princess Máxima Center, Utrecht | None | None | None |
| Gestel, van | Pediatrician intensivist, Wilhelmina Children’s Hospital, Utrecht | None | None | None |
| Hagleitner | Pediatric oncologist, Princess Máxima Center, Utrecht | None | None | None |
| Heitink-Pollé | Pediatric hematologist-oncologist, Princess Máxima Center, Utrecht | Chairman of the editorial board of the children’s hematology workbook | None | None |
| Huisman | Pediatrician-hematologist/Transfusion specialist, Sophia Children’s Hospital (Erasmus MC, Rotterdam) | Transfusion specialist Unit Transfusion Medicine, Sanquin Blood Supply | None | None |
| Janssens | Pediatric radiation therapist, Wilhelmina Children’s Hospital, Utrecht | None | None | None |
| Kuijper | Laboratory specialist hematology, Máxima Medical Center, Veldhoven | Lecturer at Fontys University of Applied Sciences | None | None |
| Mensink | Pediatric anesthetist, Princess Máxima Center, Utrecht | Board member of the pain and palliative medicine section NVA | None | None |
| Nijman | Pediatrician intensivist, Wilhelmina Children’s Hospital, Utrecht | None | None | None |
| Noordzij | Pediatrician-infectiologist/immunologist, Reinier de Graaf Gasthuis, Delft | None | None | None |
| Ophorst | Pediatric oncology nurse, expert nursing research, Princess Máxima Center, Utrecht | None | None | None |
| Plieger | Policy officer VKN (Dutch Children’s Cancer Association) | None | None | None |
| Spijkerman | Pediatrician, Pediatric Oncology Fellow, Princess Máxima Center, Utrecht | None | None | None |
| Steeg, van der | Pediatric surgeon, Princess Máxima Center, Utrecht | None | None | None |
| Wetering, van de | SKION Supportive Care task group, Pediatric oncologist, Princess Máxima Center, Utrecht | None | None | None |
