# Supplementary material for: Prophylactic red blood cell transfusions in children and neonates with cancer: An evidence-based clinical practice guideline
Source: Support Care Cancer. 2024 Nov 4;32(11):766. doi: 10.1007/s00520-024-08888-3 (PMC11534970; doi:10.1007/s00520-024-08888-3)
Supplement: Supplementary file 2 — Supplementary file2 (DOCX 7 KB) [file 520_2024_8888_MOESM2_ESM.docx]

**Supplemental Materials S2: Overview of the clinical questions**

What is the effect of prophylactic RBC transfusion on quality of life and other outcomes in children with cancer?
P = Children (aged 28 days-18 years) with cancer receiving anti-cancer treatment with curative intent*
I = Prophylactic RBC transfusion (at any threshold)
C = (No prophylactic RBC transfusion or transfusion at any other threshold)
O = Quality of life, transfusion-related complications, treatment-related complications, morbidity, mortality, admission to hospital, costs, late complications, event-free survival
* Excluding the subgroups defined by the authors (e.g. sepsis and cardiac and/or pulmonary comorbidities).

What is the effect of prophylactic RBC transfusion on quality of life and other outcomes in neonates with cancer?
P = Neonates (aged 0-28 days) with cancer receiving anti-cancer treatment with curative intent*
 I = Prophylactic RBC transfusion (at any threshold)
 C = (No prophylactic RBC transfusion or transfusion at any other threshold)
 O = Quality of life, transfusion-related complications, treatment-related complications, morbidity, mortality, admission to hospital, costs, late complications, event-free survival
* Excluding the subgroups defined by the authors (e.g. sepsis and cardiac and/or pulmonary comorbidities).

What is the effect of prophylactic RBC transfusion on quality of life and other outcomes in children with cancer who suffer from sepsis (author-defined)?
P = Children (aged 28 days-18 years) with cancer with curative intent who suffer from sepsis (author-defined)
I = Prophylactic RBC transfusion (at any threshold)
C = (No prophylactic RBC transfusion or transfusion at any other threshold)
O = Quality of life, transfusion-related complications, treatment-related complications, morbidity, mortality, admission to hospital, costs, late complications, event-free survival

What is the effect of prophylactic RBC transfusion on quality of life and other outcomes in neonates with cancer who suffer from sepsis (author-defined)?
P = Neonates (aged 0-28 days) with cancer with curative intent who suffer from sepsis (author-defined)
I = Prophylactic RBC transfusion (at any threshold)
C = (No prophylactic RBC transfusion or transfusion at any other threshold)
O = Quality of life, transfusion-related complications, treatment-related complications, morbidity, mortality, admission to hospital, costs, late complications, event-free survival

What is the effect of prophylactic RBC transfusion on quality of life and other outcomes in children with cancer who undergo radiotherapy?
P = Children (aged 28 days-18 years) with cancer who undergo radiotherapy with curative intent
 I = Prophylactic RBC transfusion (at any threshold)
 C = (No prophylactic RBC transfusion or transfusion at any other threshold)
 O = Quality of life, transfusion-related complications, treatment-related complications, morbidity, mortality, admission to hospital, costs, late complications, event-free survival

What is the effect of prophylactic RBC transfusion on quality of life and other outcomes in neonates with cancer who undergo radiotherapy?
P = Neonates (aged 0-28 days) with cancer who undergo radiotherapy with curative intent
I = Prophylactic RBC transfusion (at any threshold)
C = (No prophylactic RBC transfusion or transfusion at any other threshold)
O = Quality of life, transfusion-related complications, treatment-related complications, morbidity, mortality, admission to hospital, costs, late complications, event-free survival

What is the effect of prophylactic RBC transfusion on patient-related and other outcomes in children with cancer with cardiac and/or pulmonary comorbidity?
P = Children (aged 28 days-18 years) with cancer with curative intent and cardiac and/or pulmonary comorbidity (ASA equal to or bigger than 3)
I = Prophylactic RBC transfusion (at any threshold)
C = (No prophylactic RBC transfusion or transfusion at any other threshold)
O = Quality of life, transfusion-related complications, treatment-related complications, morbidity, mortality, admission to hospital, costs, late complications, event-free survival

What is the effect of prophylactic RBC transfusion on patient-related and other outcomes in neonates with cancer with cardiac and pulmonary comorbidity?
P = Neonates (aged 0-28 days) with cancer with curative intent and cardiac and/or pulmonary comorbidity (ASA equal to or bigger than 3)
I = Prophylactic RBC transfusion (at any threshold)
C = (No prophylactic RBC transfusion or transfusion at any other threshold)
O = Quality of life, transfusion-related complications, treatment-related complications, morbidity, mortality, admission to hospital, costs, late complications, event-free survival

What is the effect of prophylactic RBC transfusion on quality of life and other outcomes in children with cancer and hyper leukocytosis (author-defined)?
P = Children with cancer (aged 28 days-18 years) with curative intent and hyperleukocytosis (author-defined)
I = Prophylactic RBC transfusion (at any threshold)
C = No prophylactic RBC transfusion or transfusion at any other threshold
O = Quality of life, transfusion-related complications, treatment-related complications, morbidity, mortality, admission to hospital, costs, late complications

What is the effect of prophylactic RBC transfusion on quality of life and other outcomes in neonates with cancer and hyper leukocytosis (author-defined)?
P = Neonates with cancer (aged 0-28 days) with curative intent and hyperleukocytosis (author-defined)
I = Prophylactic RBC transfusion (at any threshold)
C = No prophylactic RBC transfusion or transfusion at any other threshold
O = Quality of life, transfusion-related complications, treatment-related complications, morbidity, mortality, admission to hospital, costs, late complications

What is the effect of irradiated RBC products on complications and other outcomes in children with cancer who need to undergo a RBC transfusion?
P = Children with cancer (aged 28 days-18 years) with curative intent who need to undergo a RBC transfusion for any indication
I = Irradiated RBC products
C = Non irradiated RBC products
O = Quality of life, transfusion-related complications, treatment-related complications, morbidity, mortality, admission to hospital, costs, late complications

What is the effect of irradiated RBC products on complications and other outcomes in neonates with cancer who need to undergo a RBC transfusion?
P = Neonates with cancer (aged 0-28 days) with curative intent who need to undergo a RBC transfusion for any indication
I = Irradiated RBC products
C = Non irradiated RBC products
O = Quality of life, transfusion-related complications, treatment-related complications, morbidity, mortality, admission to hospital, costs, late complications

What is the effect of low-volume prophylactic RBC transfusion compared to high-volume RBC transfusion on quality of life and other outcomes in children with cancer?
P = Children with cancer (aged 28 days-18 years) with curative intent
I = Low-volume RBC transfusion (at any threshold)
C = High-volume RBC transfusion
O = Quality of life, transfusion-related complications, treatment-related complications, morbidity, mortality, admission to hospital, costs, late complications

What is the effect of low-volume prophylactic RBC transfusion compared to high-volume RBC transfusion on quality of life and other outcomes in neonates with cancer?
P = Neonates with cancer (aged 0-28 days) with curative intent
I = Low-volume prophylactic RBC transfusion (at any threshold)
C = High-volume prophylactic RBC transfusion
O = Quality of life, transfusion-related complications, treatment-related complications, morbidity, mortality, admission to hospital, costs, late complications

What is the effect of prophylactic RBC transfusion at any infusion rate compared to RBC transfusion at any other infusion rate on quality of life and other outcomes in children with cancer?
P = Children with cancer (aged 28 days-18 years) with curative intent who need to undergo a RBC transfusion for any indication
I = Prophylactic RBC transfusion at any infusion rate (at any threshold)
C = Prophylactic RBC transfusion at any other infusion rate
O = Quality of life, transfusion-related complications, treatment-related complications, morbidity, mortality, admission to hospital, costs, late complications

What is the effect of prophylactic RBC transfusion at any infusion rate compared to RBC transfusion at any other infusion rate on quality of life and other outcomes in neonates with cancer?
P = Neonates with cancer (aged 0-28 days) with curative intent who need to undergo a RBC transfusion for any indication
I = Prophylactic RBC transfusion at any infusion rate (at any threshold)
C = Prophylactic RBC transfusion at any other infusion rate
O = Quality of life, transfusion-related complications, treatment-related complications, morbidity, mortality, admission to hospital, costs, late complication
