# Supplementary material for: Prophylactic red blood cell transfusions in children and neonates with cancer: An evidence-based clinical practice guideline
Source: Support Care Cancer. 2024 Nov 4;32(11):766. doi: 10.1007/s00520-024-08888-3 (PMC11534970; doi:10.1007/s00520-024-08888-3)
Supplement: Supplementary file 3 — Supplementary file3 (DOCX 10 KB) [file 520_2024_8888_MOESM3_ESM.docx]

**Supplemental Materials S3:** Systematic literature search
Performed by: Mw. H.W.J. (Rikkie) Deurenberg, research specialist

Cochrane search
ID Search
#1 (Cancer OR cancers OR cancer* OR oncology OR oncolog* OR neoplasm OR neoplasms OR neoplasm* OR carcinoma OR carcinom* OR tumor OR tumour OR tumor* OR tumour* OR tumors OR tumours OR malignan* OR malignant OR hematooncological OR hemato oncological OR hemato-oncological OR hematologic neoplasms OR hematolo*):ti,ab,kw (Word variations have been searched)
#2 "P variant breed":ti
#3 MeSH descriptor: [Stem Cell Transplantation] explode all trees
#4 stem NEAR/2 cell NEAR/3 transplan*:ti,ab
#5 stem NEAR/2 cell NEAR/3 transplan*:kw
#6 [mh "bone marrow transplantation"]
#7 "bone marrow" NEAR/5 transplant*:ti,ab,kw
#8 "stem cell" NEAR/5 transplant*:ti,ab,kw
#9 #3 OR #4 OR #5 OR #6 OR #7 OR #8
#10 [**Error**]==>"P variant stam cel transplantatie".ti.
#11 MeSH descriptor: [Leukemia] explode all trees
#12 (leukemia or leukemi* or leukaemi*):ti,ab,kw
#13 (aml or anll or lymphoma or lymphom* or hodgkin* or T-cell or B-cell or non-hodgkin or sarcoma or sarcom* or Ewing* or osteosarcom* or wilms* or nephroblastom* or neuroblastom* or rhabdomyosarcom* or teratom* or hepatom* or hepatoblastom* or PNET or medulloblastom* or PNET* or (neuroectodermal adj2 tumors NEAR/2 primitive) or retinoblastoma or retinoblastom* or meningiom* or gliom*):ti,ab,kw
#14 [mh "lymphatic vessel tumors"]
#15 MeSH descriptor: [Lymphatic Vessel Tumors] explode all trees
#16 [mh lymphoma] OR [mh "neoplasms, complex and mixed"] OR [mh "neoplasms, connective and soft tissue"] OR [mh "neoplasms, germ cell and embryonal"] OR [mh "neoplasms, glandular and epithelial"] OR [mh “neoplasms, gonadal tissue”] OR [mh “neoplasms, nerve tissue”] OR [mh “neoplasms, plasma cell”] OR [mh “neoplasms, vascular tissue"] OR [mh “neoplasms by site"] OR [mh “neoplasms, hormone-dependent"] OR [mh “neoplasms, radiation-induced"] OR [mh “neoplastic syndromes, hereditary”]
#17 ((brain NEAR/1 tumor*) OR (brain NEAR/1 tumour) OR (brain NEAR/1 neoplasm*) or (central NEAR/1 nervous NEAR/1 system NEAR/1 neoplasm*) OR (central NEAR/1 nervous NEAR/1 system NEAR/1 tumo*) or (central NEAR/1 nervous NEAR/1 system NEAR/1 cancer*) or (brain NEAR/1 cancer*) or (brain NEAR/1 neoplasm*) or (intracranial NEAR/1 neoplasm*) or (leukemia NEAR/1 lymphocytic NEAR/1 acute*)):ti,ab,kw
#18 #11 OR #12 OR #13 OR #14 OR #15 OR #16 OR #17
#19 #1 OR #9 OR #18
#20 ((pediatric NEAR/3 oncolog*) OR (paediatric NEAR/3 oncol*) OR (child* NEAR/3 (cancer* OR tumor* OR tumour* OR neoplasm*))):ti,ab,kw
#21 [mh "young adult"] OR [mh child] OR [mh infant]
#22 ((young NEAR/1 adult*) OR child* OR infant* OR pediatr* OR paediatr* OR perinat* OR neonat* OR newborn* OR infan* OR boy OR boys OR girl OR girls OR kid OR kids or schoolage* or juvenil* or teenage* or adolescen* or toddler*):ti,ab,kw
#23 #20 OR #21 OR #22
#24 #18 AND #23
#25 [mh "Platelet Transfusion"]
#26 [mh Plateletpheresis]
#27 [mh "Blood Platelets"]
#28 ((platelet* OR thrombocyte*) NEAR/5 (prophyla* OR transfus* OR infus* OR administ* OR requir* OR need* OR product* OR component* OR concentrate* OR apheres* OR pooled OR single NEAR/1 donor OR random NEAR/1donor)):ti,ab,kw
#29 (thrombocytopheres* or plateletpheres*):ti,ab,kw
#30 #25 OR #26 OR #27 OR #28 OR #29
#31 [mh "blood component transfusion"] OR [mh "erythrocyte transfusion"]
#32 ((blood NEAR/3 transfus*) or (erythrocyt* NEAR/2 transfus*)):ti,ab,kw
#33 ((erythrocy* OR hemoglobin* OR haemoglobin*) NEAR/5 (prophyla* OR transfus* OR infus* OR administ* OR requir* OR need* OR product* OR component* OR concentrate* OR apheres* OR pooled OR single NEAR/1 donor OR random NEAR/1donor)):ti,ab,kw
#34 #31 OR #32 OR #33
#35 [mh "blood component transfusion"] OR [mh "erythrocyte transfusion"] OR [mh "platelet transfusion"]
#36 [mh "Platelet Count"]
#37 #35 OR #36
#38 #30 OR #34 OR #37
#39 #24 AND #38

Medline search
Database: Ovid MEDLINE(R) ALL <1946 to December 11, 2020>
Search Strategy:
--------------------------------------------------------------------------------
1 exp stem cell transplantation/ or exp hematopoietic stem cell transplantation/ (83929)
2 (stem adj2 cell adj3 transplan*).tw. (50727)
3 (stem adj2 cell adj3 transplan*).kf. (7551)
4 bone marrow transplantation/ (44746)
5 ("bone marrow" adj5 transplant$).tw. (38419)
6 ("bone marrow" adj5 transplant$).kf. (1865)
7 ("stem cell" adj5 transplant$).tw. (51747)
8 ("stem cell" adj5 transplant$).kf. (7713)
9 or/1-8 (151154)
10 exp Leukemia/ (234051)
11 (leukemia or leukemi* or leukaemi*).tw. (269738)
12 (leukemia or leukemi* or leukaemi*).kf. (32354)
13 (aml or anll or lymphoma or lymphom* or hodgkin* or T-cell or B-cell or non-hodgkin or sarcoma or sarcom* or Ewing* or osteosarcom* or wilms* or nephroblastom* or neuroblastom* or rhabdomyosarcom* or teratom* or hepatom* or hepatoblastom* or PNET or medulloblastom* or PNET* or (neuroectodermal adj2 tumors adj2 primitive) or retinoblastoma or retinoblastom* or meningiom* or gliom*).tw. (834591)
14 (aml or anll or lymphoma or lymphom* or hodgkin* or T-cell or B-cell or non-hodgkin or sarcoma or sarcom* or Ewing* or osteosarcom* or wilms* or nephroblastom* or neuroblastom* or rhabdomyosarcom* or teratom* or hepatom* or hepatoblastom* or PNET or medulloblastom* or PNET* or (neuroectodermal adj2 tumors adj2 primitive) or retinoblastoma or retinoblastom* or meningiom* or gliom*).kf. (90826)
15 exp lymphatic vessel tumors/ or exp lymphoma/ or exp "neoplasms, complex and mixed"/ or exp "neoplasms, connective and soft tissue"/ or exp "neoplasms, germ cell and embryonal"/ or exp "neoplasms, glandular and epithelial"/ or exp neoplasms, gonadal tissue/ or exp neoplasms, nerve tissue/ or exp neoplasms, plasma cell/ or exp neoplasms, vascular tissue/ or exp neoplasms by site/ or exp neoplasms, hormone-dependent/ or exp neoplasms, radiation-induced/ or exp neoplastic syndromes, hereditary/ (2727316)
16 ((brain adj tumo?r*) or (brain adj neoplasm?) or (central adj nervous adj system adj neoplasm?) or (central adj nervous adj system adj tumo?r?) or (central adj nervous adj system adj cancer?) or (brain adj cancer*) or (brain adj neoplasm*) or (intracranial adj neoplasm*) or (leukemia adj lymphocytic adj acute*)).tw. (49779)
17 ((brain adj tumo?r*) or (brain adj neoplasm?) or (central adj nervous adj system adj neoplasm?) or (central adj nervous adj system adj tumo?r?) or (central adj nervous adj system adj cancer?) or (brain adj cancer*) or (brain adj neoplasm*) or (intracranial adj neoplasm*) or (leukemia adj lymphocytic adj acute*)).kf. (10686)
18 or/10-17 (3380178)
19 "variant neurocognitive P".ti. (0)
20 "P variant breed".ti. (0)
21 ((p?ediatric adj3 oncolog*) or (child* adj3 (cancer? or tumo?r? or neoplasm?))).tw. (39721)
22 ((p?ediatric adj3 oncolog*) or (child* adj3 (cancer? or tumo?r? or neoplasm?))).kf. (2540)
23 young adult/ or exp child/ or exp infant/ (3206941)
24 ((young adj adult?) or child??? or childhood or infant* or p?ediatr* or perinat* or neonat* or newborn* or infan* or boy? or girl? or kid? or schoolage* or juvenil* or teenage* or adolescen* or toddler?).tw. (2465097)
25 ((young adj adult?) or child??? or childhood or infant* or p?ediatr* or perinat* or neonat* or newborn* or infan* or boy? or girl? or kid? or schoolage* or juvenil* or teenage* or adolescen* or toddler?).kf. (336883)
26 or/21-25 (4126178)
27 (cancer* or oncolog* or neoplasm* or carcinom* or tumor* or tumour* or malignan* or hematooncological or hemato?oncological or hemato-oncological or (hematologic adj neoplasm*)).tw. (3361279)
28 (cancer* or oncolog* or neoplasm* or carcinom* or tumor* or tumour* or malignan* or hematooncological or hemato?oncological or hemato-oncological or (hematologic adj neoplasm*)).kf. (611739)
29 9 or 18 or 27 or 28 (4682141)
30 "P in 3 varianten".ti. (0)
31 21 or 22 or 29 (4682141)
32 "P 3 variaties of kinderoncologie".ti. (0)
33 23 or 24 or 25 (4126038)
34 31 and 33 (506334)
35 29 and 31 (4682141)
36 9 or 18 (3468080)
37 33 and 36 (413142)
38 "onderdeel transfusies".ti. (0)
39 exp Platelet Transfusion/ (7273)
40 Plateletpheresis/ (1486)
41 Blood Platelets/ (77417)
42 ((platelet* or thrombocyte*) adj5 (prophyla* or transfus* or infus* or administ* or requir* or need* or product* or component* or concentrate* or apheres* or pooled or single donor or random donor)).tw,kf. (24757)
43 (thrombocytopheres* or plateletpheres*).tw,kf. (606)
44 ((platelet* or thrombocyte*) adj5 (protocol* or trigger* or threshold* or schedul* or dose* or dosing or usage or utili?ation)).tw,kf. (5706)
45 (platelet* or thrombocyte*).ti. (94077)
46 or/39-45 (133917)
47 blood component transfusion/ or erythrocyte transfusion/ (12677)
48 ((blood adj3 transfus*) or (erythrocyt* adj2 transfus*)).tw,kf. (65682)
49 ((erythrocy* or h?emoglobin*) adj5 (prophyla* or transfus* or infus* or administ* or requir* or need* or product* or component* or concentrate* or apheres* or pooled or single donor or random donor)).tw,kf. (13816)
50 47 or 48 or 49 (82926)
51 46 or 50 (211321)
52 "onderdeel transfusies".ti. (0)
53 37 and 51 (3603)
54 35 and 51 (29143)
55 exp Case Reports/ (2140140)
56 (case adj2 serie?).ti,ab,kf. (81166)
57 55 or 56 (2206004)
58 53 not 57 (2895)
59 blood component transfusion/ or erythrocyte transfusion/ or platelet transfusion/ (18929)
60 Platelet Count/ (21987)
61 transfus*.ti,kf. (46897)
62 46 or 50 or 59 or 60 or 61 (236803)
63 37 and 62 (4385)
64 63 not 57 (3528)
65 64 (3528)

Embase search
Database: Embase <1974 to 2020 December 10>
Search Strategy:

--------------------------------------------------------------------------------
1 exp stem cell transplantation/ or exp allogeneic stem cell transplantation/ (156010)
2 exp hematopoietic stem cell transplantation/ (67381)
3 (stem adj2 cell adj3 transplan*).tw,kw. (98779)
4 bone marrow transplantation/ (51280)
5 ("bone marrow" adj5 transplant$).tw,kw. (55498)
6 ("stem cell" adj5 transplant$).tw,kw. (100630)
7 or/1-6 (236441)
8 exp leukemia/ (308369)
9 (leukemia or leukemi* or leukaemi*).tw,kw. (360281)
10 (aml or anll or lymphoma or lymphom* or hodgkin* or T-cell or B-cell or non-hodgkin or sarcoma or sarcom* or Ewing* or osteosarcom* or wilms* or nephroblastom* or neuroblastom* or rhabdomyosarcom* or teratom* or hepatom* or hepatoblastom* or PNET or medulloblastom* or PNET* or (neuroectodermal adj2 tumors adj2 primitive) or retinoblastoma or retinoblastom* or meningiom* or gliom*).tw,kw. (1143260)
11 exp lymphangioma/ (8661)
12 exp lymphoma/ (301746)
13 (neoplasms, complex and mixed).mp. [mp=title, abstract, heading word, drug trade name, original title, device manufacturer, drug manufacturer, device trade name, keyword, floating subheading word, candidate term word] (40)
14 (neoplasms, connective and soft tissue).mp. [mp=title, abstract, heading word, drug trade name, original title, device manufacturer, drug manufacturer, device trade name, keyword, floating subheading word, candidate term word] (15)
15 (neoplasms, germ cell and embryonal).mp. [mp=title, abstract, heading word, drug trade name, original title, device manufacturer, drug manufacturer, device trade name, keyword, floating subheading word, candidate term word] (176)
16 (neoplasms, glandular and epithelial).mp. [mp=title, abstract, heading word, drug trade name, original title, device manufacturer, drug manufacturer, device trade name, keyword, floating subheading word, candidate term word] (170)
17 exp gonad tumor/ (178138)
18 exp nerve tumor/ (53565)
19 plasmacytoma/ (11334)
20 exp vascular tumor/ (80957)
21 exp neoplasms subdivided by anatomical site/ (4055653)
22 neoplasms, hormone-dependent.mp. (47)
23 radiation induced neoplasm/ (2365)
24 exp hereditary tumor syndrome/ (47443)
25 or/8-24 (4628664)
26 ((brain adj tumo?r*) or (brain adj neoplasm?) or (central adj nervous adj system adj neoplasm?) or (central adj nervous adj system adj tumo?r?) or (central adj nervous adj system adj cancer?) or (brain adj cancer*) or (brain adj neoplasm*) or (intracranial adj neoplasm*) or (leukemia adj lymphocytic adj acute*)).tw,kw. (77614)
27 25 or 26 (4633475)
28 (cancer* or oncolog* or neoplasm* or carcinom* or tumor* or tumour* or malignan* or hematooncological or hemato?oncological or hemato-oncological or (hematologic adj neoplasm*)).tw,tw. (4490931)
29 7 or 27 or 28 (6033104)
30 "P in 3 varianten".ti. (0)
31 ((p?ediatric adj3 oncolog*) or (child* adj3 (cancer? or tumo?r? or neoplasm?))).tw,kw. (60814)
32 29 or 31 (6033185)
33 young adult/ (381995)
34 child/ or boy/ or girl/ or exp infant/ or preschool child/ or school child/ or toddler/ (2680621)
35 exp infant/ (1007136)
36 ((young adj adult?) or child??? or childhood or infant* or p?ediatr* or perinat* or neonat* or newborn* or infan* or boy? or girl? or kid? or schoolage* or juvenil* or teenage* or adolescen* or toddler?).tw,kw. (3075029)
37 or/33-36 (4109692)
38 32 and 37 (544704)
39 (7 or 27) and 37 (447217)
40 "onderdeel transfusies".ti. (0)
41 blood component therapy/ or erythrocyte transfusion/ or granulocyte transfusion/ or leukocyte transfusion/ or lymphocyte transfusion/ or thrombocyte transfusion/ (48068)
42 thrombocytopheresis/ (1878)
43 ((platelet* or thrombocyte*) adj5 (prophyla* or transfus* or infus* or administ* or requir* or need* or product* or component* or concentrate* or apheres* or pooled or single donor or random donor)).tw,kw. (39914)
44 (thrombocytopheres* or plateletpheres*).tw,kw. (924)
45 ((platelet* or thrombocyte*) adj5 (protocol* or trigger* or threshold* or schedul* or dose* or dosing or usage or utili?ation)).tw,kw. (9210)
46 (platelet* or thrombocyte*).ti. (116472)
47 ((blood adj3 transfus*) or (erythrocyt* adj2 transfus*)).tw,kw. (94695)
48 ((erythrocy* or h?emoglobin*) adj5 (prophyla* or transfus* or infus* or administ* or requir* or need* or product* or component* or concentrate* or apheres* or pooled or single donor or random donor)).tw,kw. (19578)
49 platelet count/ (23320)
50 blood transfusion/ (123544)
51 transfus*.ti,kw. (53810)
52 or/41-51 (351651)
53 or/41-49,51 (300466)
54 53 and 39 (7377)
55 case report/ (2565963)
56 case study/ (74534)
57 (case adj2 serie?).ti,ab,kw. (114100)
58 or/55-57 (2683514)
59 54 not 58 (4879)
60 59 (4879)
61 limit 60 to embase status (2920)
62 52 and 39 (9143)
63 62 not 58 (5988)
64 limit 63 to embase status (3704)
65 64 not 61 (784)
66 60 (4879)
