# Supplementary material for: Prophylactic red blood cell transfusions in children and neonates with cancer: An evidence-based clinical practice guideline
Source: Support Care Cancer. 2024 Nov 4;32(11):766. doi: 10.1007/s00520-024-08888-3 (PMC11534970; doi:10.1007/s00520-024-08888-3)

**Supplemental Materials S4: Risk of Bias-tool & results**

Risk of Bias-tool for Non-RCTs
As only three of the studies were RCTs, the methodology for Risk of Bias assessment had to be complemented by a non-RCT risk of bias tool. We therefore combined the Risk of Bias tool for observational studies, as described in the IGHG Handbook (2), with a couple of aspects of the RCT tool as described earlier. By combining these tools, we aimed to have the best possible tool to assess the Risk of Bias in these types of studies.

**Table 1.** Adjusted Risk of Bias criteria for non-RCTs (4)

| Selection bias | Is the study group representative?  Cases and controls were selected based on comparable patient characteristics (i.e. age, gender and tumor type)  *Low risk if*: no significant differences between cases and controls with respect to age, gender and tumor type.  *High risk if*: cases and controls differ with respect to age, gender and tumor type (baseline imbalances caused by selection). |
| --- | --- |
|  |  |
| Attrition bias | Is complete outcome data for all the participants available in this study? Is the follow up adequate?  *Low risk if:* no missing data, reasons for missing data not related to outcome, missing data balanced across groups, proportion missing or plausible effect size not enough to have a clinically relevant effect  *High risk if:* imbalance in numbers or reasons, proportion missing or plausible effect size enough to have a clinically relevant effect, inappropriate use of imputation, ‘as treated’ analysis with substantial departure from allocation |
|  |  |
| Detection bias | Are the outcome assessors blinded for important determinants related to the outcome?  *Low risk if*: the outcome assessors were blinded for important determinants related to the outcome  *High risk if*: no blinding or broken blinding **and** measurement is likely to be influenced |
|  |  |
| Reporting bias | Is the report complete? Are the outcomes that were planned to be measured also reported?  *High risk if*: Outcomes are not reported as pre-specified or expected and/or outcomes are reported incompletely so they cannot be entered in a meta-analysis |
|  |  |
| Confounding bias | Are the analyses adjusted for important confounding factors?  *Low risk if*: important prognostic factors (i.e. age, gender, diagnosis and risk stratification) were taken adequately into account  *High risk if*: important prognostic factors (i.e. age, genders, diagnosis and risk stratification) were inadequately or not taken into account |
|  |  |
| Other bias | The following list of other potential sources of bias in a clinical study may aid detection of further problems.  *High if:* - The conduct of the study is affected by interim results (e.g. recruiting additional participants from a subgroup showing more benefit). - There is deviation from the study protocol in a way that does not reflect clinical practice (e.g. *post hoc* stepping-up of doses to exaggerated levels). - There is pre-randomization administration of an intervention that could enhance or diminish the effect of a subsequent, randomized, intervention. - Inappropriate administration of an intervention (or co-intervention). - Contamination (e.g. participants pooling drugs). - Occurrence of ‘null bias’ due to interventions being insufficiently well delivered or overly wide inclusion criteria for participants (Woods 1995). - An insensitive instrument is used to measure outcomes (which can lead to under-estimation of both beneficial and harmful effects). - Selective reporting of subgroups. - Fraud. - Baseline imbalances for other reasons than through selection - Other |
|  |  |

**Figure 1.** Risk of bias - RCTs

**
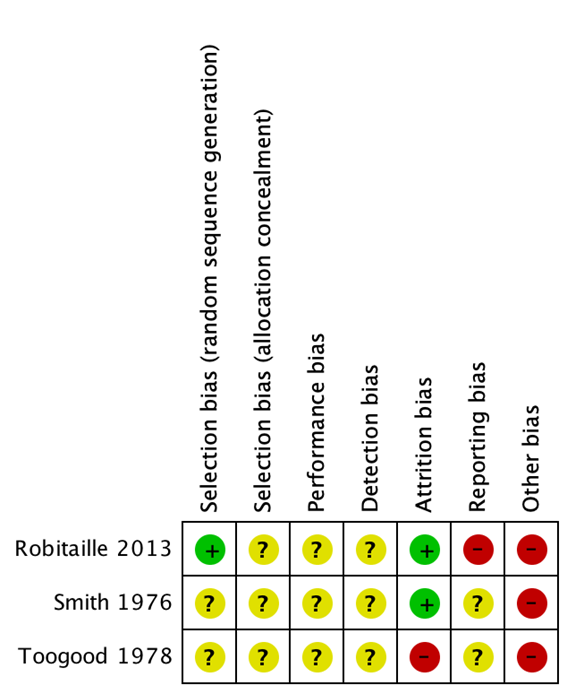
**

**Figure 2.** Risk of bias - non-RCTs


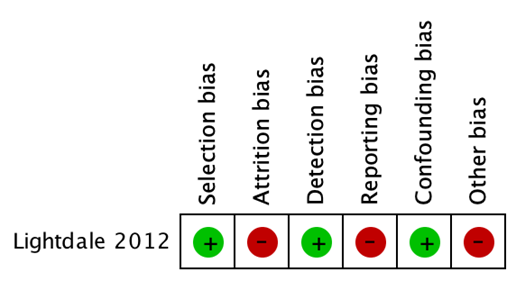

Supplement: Supplementary file 4 — Supplementary file4 (DOCX 103 KB) [file 520_2024_8888_MOESM4_ESM.docx]
