# Supplementary material for: Prophylactic red blood cell transfusions in children and neonates with cancer: An evidence-based clinical practice guideline
Source: Support Care Cancer. 2024 Nov 4;32(11):766. doi: 10.1007/s00520-024-08888-3 (PMC11534970; doi:10.1007/s00520-024-08888-3)
Supplement: Supplementary file 5 — Supplementary file5 (DOCX 19.2 KB) [file 520_2024_8888_MOESM5_ESM.docx]

**Supplemental materials S5: Description of the additional guidelines (including AGREE II-scores)**Valentine (2018)
Valentine (2018) created recommendations for RBC transfusions in critically ill children by bringing together international, multidisciplinary experts in a guideline panel. This to develop evidence-based and when evidence is lacking, expert-based consensus statements to guide transfusion and blood management practices. Thirty-eight content experts and 4 non-voting methodology and implementation experts, representing 8 countries, 29 academic institutions and 8 medical specialties, agreed and participated in all aspects. The following 9 clinical subtopics: indications for RBC transfusion based on Hb and physiologic thresholds in critically ill children 1) in the general PICU population, with 2) respiratory failure, 3) non-hemorrhagic shock, 4) non-life threatening bleeding and hemorrhagic shock, 5) acute brain injury, 6) acquired and congenital heart disease, 7) sickle cell and oncologic disease, 8) support from extracorporeal membrane oxygenation, ventricular assist devices, renal replacement therapy, and 9) the use of alternative processing of blood products are discussed. They conducted a systematic review for the 9 subtopics and analyzed the evidence using the GRADE methodology (33).

*AGREE II assessment*- Domain 1. Scope and Purpose: The scaled domain score = 89%
- Domain 2. Stakeholder Involvement: The scaled domain score = 44%
- Domain 3. Rigour of Development: The scaled domain score = 79%
- Domain 4. Clarity of Presentation: The scaled domain score = 78%
- Domain 5. Applicability: The scaled domain score = 63%
- Domain 6. Editorial Independence: The scaled domain score = 92%
- Overall Guideline Assessment: Score 5 (I would recommend this guideline for use)

JPAC (2013)
The Joint United Kingdom Blood Transfusion Services Professional Advisory Committee (JPAC) has the purpose to deliver detailed service guidelines for blood transfusions and is an advisory organ to the United Kingdom Blood Services. And they created a transfusion handbook (2013) and based their advice on other guidelines (41).

*AGREE II assessment*- Domain 1. Scope and Purpose: The scaled domain score = 94%
- Domain 2. Stakeholder Involvement: The scaled domain score = 56%
- Domain 3. Rigour of Development: The scaled domain score = 35%
- Domain 4. Clarity of Presentation: The scaled domain score = 56%
- Domain 5. Applicability: The scaled domain score = 58%
- Domain 6. Editorial Independence: The scaled domain score = 25%
- Overall Guideline Assessment: Score 4 (I would recommend this guideline for use, with modifications)

CBO (2011)
CBO (2011) created a guideline that consists of recommendations for the blood transfusion practice and the underlying arguments for these recommendations. These recommendations were established through extensive literature research and subsequent opinions within the multidisciplinary guideline panel that consisted of delegate representatives of the various professional associations involved. The literature research was performed according to the Evidence-Based Guideline method Development (EBRO). Initially, the search was focused on evidence-based guidelines and review in the period from early 2003 to February 2008. These guidelines and reviews were judged for quality using the AGREE instruments and the evidence was used from these guidelines to answer the research question. Then, there were searches for additional studies per chapter from the moment the search in the guideline and/or review ended (42).

*AGREE II assessment*- Domain 1. Scope and Purpose: The scaled domain score = 89%
- Domain 2. Stakeholder Involvement: The scaled domain score = 94%
- Domain 3. Rigour of Development: The scaled domain score = 85%
- Domain 4. Clarity of Presentation: The scaled domain score = 61%
- Domain 5. Applicability: The scaled domain score = 50%
- Domain 6. Editorial Independence: The scaled domain score = 0%
- Overall Guideline Assessment: Score 5 (I would recommend this guideline for use)

NICE (2015)
The National Institute for Health and Care Excellence (NICE) (2015) provides national guidance and advice to improve health and social care. NICE produces evidence-based guidance and advice for health, public health and social care practitioners and develops quality standards and performance metrics (43).

*AGREE II assessment*- Domain 1. Scope and Purpose: The scaled domain score = 100%
- Domain 2. Stakeholder Involvement: The scaled domain score = 89%
- Domain 3. Rigour of Development: The scaled domain score = 69%
- Domain 4. Clarity of Presentation: The scaled domain score = 94%
- Domain 5. Applicability: The scaled domain score = 54%
- Domain 6. Editorial Independence: The scaled domain score = 92%
- Overall Guideline Assessment: Score 5 (I would recommend this guideline for use)

New (2016)
New (2016) created a revision of the 2004 British Committee for Standards in Haematology (BCSH) guideline on transfusion in neonates and older children. The guideline writing group was a selection of medical representatives including specialists from fetal medicine, neonatology, pediatric intensive care, cardiac anesthesia, pediatric hematology, clinical and laboratory transfusion medicine. The guideline is based on a systematic literature search after the 2004 guideline up to November 2014. This together with other relevant papers identified. The guideline was externally reviewed by the members of the Transfusion Task Force of the BCSH and by a sounding board including UK hematologists, pediatricians, and neonatologists. The evidence was graded according to the GRADE method (44).

*AGREE II assessment*- Domain 1. Scope and Purpose: The scaled domain score = 83%
- Domain 2. Stakeholder Involvement: The scaled domain score = 55%
- Domain 3. Rigour of Development: The scaled domain score = 54%
- Domain 4. Clarity of Presentation: The scaled domain score = 83%
- Domain 5. Applicability: The scaled domain score = 29%
- Domain 6. Editorial Independence: The scaled domain score = 50%
- Overall Guideline Assessment: Score 4 (I would recommend this guideline for use, with modifications)

FMS (2019)
The Federation of Medical Specialists (2019) created an evidence-based blood transfusion guideline with a guideline panel. First, an exploratory search was conducted for existing foreign guidelines and systematic reviews (Medline). Subsequently, for the individual research questions, specific terms were used to search for literature in various electronic databases. Additional literature was sought based on the literature lists of the selected studies. The quality of the studies was assessed using the Risk of Bias tables and meta-analyses were performed with Review Manager 5. To assess the power of scientific evidence the GRADE method was used (22).

*AGREE II assessment*- Domain 1. Scope and Purpose: The scaled domain score = 94%
- Domain 2. Stakeholder Involvement: The scaled domain score = 100%
- Domain 3. Rigour of Development: The scaled domain score = 71%
- Domain 4. Clarity of Presentation: The scaled domain score = 89%
- Domain 5. Applicability: The scaled domain score = 13%
- Domain 6. Editorial Independence: The scaled domain score = 100%
- Overall Guideline Assessment: Score 6 (I would recommend this guideline for use)

Patient Blood Management Guidelines National Blood Authority (2012)
The Patient Blood Management Guidelines National Blood Authority (2012) created an evidence-based blood management guideline by conducting a search to answer their research questions in relevant electronic databases, bibliographies of studies that were identified as relevant and by assessing the recommended literature from experts. This was done by the Expert Working Group and the Clinical/Consumer Reference Groups. For every research question the body of evidence was consolidated into evidence statements and rated based on five domains: evidence based, consistency, clinical impact, generalizability, and applicability. Initially, studies of higher levels of evidence were included in preference over lower levels of evidence and thus minimized the bias (29).

*AGREE II assessment*- Domain 1. Scope and Purpose: The scaled domain score = 89%
- Domain 2. Stakeholder Involvement: The scaled domain score = 44%
- Domain 3. Rigour of Development: The scaled domain score = 67%
- Domain 4. Clarity of Presentation: The scaled domain score = 89%
- Domain 5. Applicability: The scaled domain score = 29%
- Domain 6. Editorial Independence: The scaled domain score = 58%
- Overall Guideline Assessment: Score 4 (I would recommend this guideline for use, with modifications)

**REFERENCES**

22. Federation of Medical Specialists (2019) Startpagina - Bloedtransfusiebeleid - Richtlijn - Richtlijnendatabase. Federation of Medical Specialists. https://richtlijnendatabase.nl/richtlijn/bloedtransfusiebeleid/startpagina_-_bloedtransfusiebeleid.html

29. Patient Blood Management Guidelines: Module 3 (2012) Patient Blood Management Guidelines National Blood Authority. https://www.blood.gov.au/pubs/pbm/module3/abbreviations_and_acronyms.html

41. JPAC. United Kingdom Blood Services (2013) Handbook of transfusion medicine 5th Edi (5th ed., 2013 editie). TSO

42. Richtlijn bloedtransfusie (2011) CBO. https://www.nvog.nl/wp-content/uploads/2018/02/Bloedtransfusie-2.0-11-11-2011.pdf

43. NICE (2015) Everview | blood transfusion | guidance | NICE. https://www.nice.org.uk/guidance/ng24/evidence/full-guideline-pdf-2177160733

44. New HV, Berryman J, Bolton-Maggs PHB, Cantwell C, Chalmers EA, Davies T, Gottstein R, Kelleher A, Kumar S, Morley SL, Stanworth SJ (2016) Guidelines on transfusion for fetuses, neonates and older children. Br J Haematol 175(5):784–828. https://doi.org/10.1111/bjh.14233
