# Supplementary material for: Prophylactic red blood cell transfusions in children and neonates with cancer: An evidence-based clinical practice guideline
Source: Support Care Cancer. 2024 Nov 4;32(11):766. doi: 10.1007/s00520-024-08888-3 (PMC11534970; doi:10.1007/s00520-024-08888-3)
Supplement: Supplementary file 6 — Supplementary file6 (DOCX 755 KB) [file 520_2024_8888_MOESM6_ESM.docx]

**Supplemental materials S6: Study characteristics of the primary studies**

Figure 1: Inclusion and exclusion process

Original search:


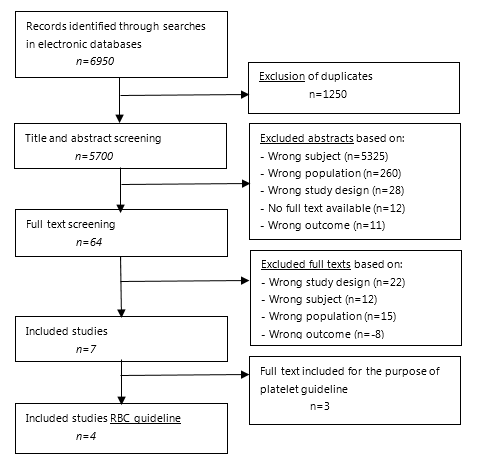

 1st update 2020:
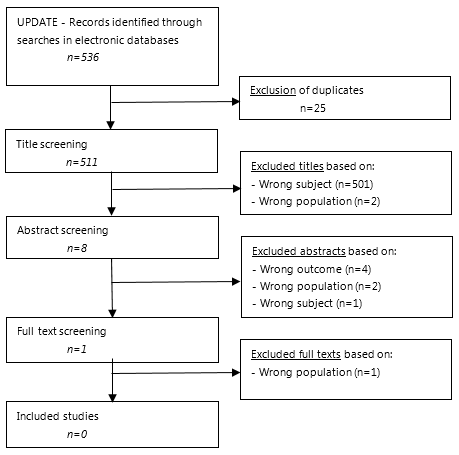


2nd update 2023


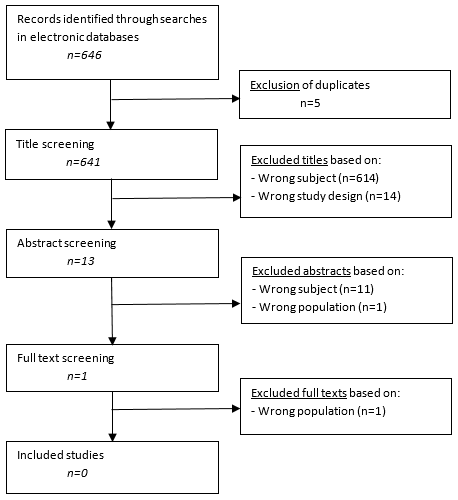


Flowchart of the inclusion and exclusion process (including the interim updates).
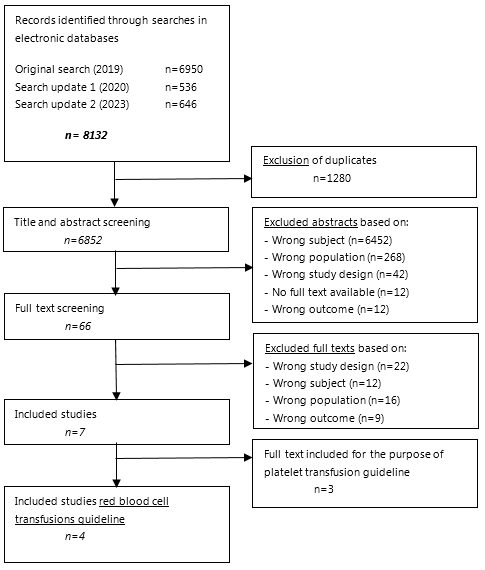


**Table 1:** Characteristics of the RCTs included (*n*=3).

| Study  **Author, year Study type** | Population **a. No. of patients b. Age (years) c. Gender (% males) d. Diagnosis** | Intervention group **a. Intervention (including dosage) b. No. of patients c. Transfusion duration/dosage** | Control Group **a. Intervention (including dosage) b. No. of patients c. Transfusion time/dosage** | Included outcomes | Risk of bias assessment  **a. Selection bias (random**  **sequence generation)**  **b. Selection bias (allocation concealment)**  **c. Performance bias**  **d. Detection bias**  **e. Attrition bias**  **f. Reporting bias**  **g. Other bias^1^** |
| --- | --- | --- | --- | --- | --- |
| Robitaille, 2013  RCT | a. 6  b. Mean 11.65 years  c. 33.3% male  d. Allogeneic HSCT | a. Hb threshold <7.5 mmol/L  b. 3 patients  c. 10-15 mL/kg | a. Hb threshold <4.3 mmol/L  b. 3 patients  c. 10-15 mL/kg | - Transfusion-related complications | a. Low b. Unclear c. Unclear d. Unclear e. Low f. High  g. High |
| Smith, 1976  RCT | a. 30  b. 3-14 years  c. 56.7% male  d. ALL, AML, Histiocytosis X, rhabdomyosarcoma | a. Hb threshold 8.69-9.93 mmol/L  b. 11 patients  c. Not stated | a. Hb threshold 6.2-7.5 mmol/L  b. 16 patients  c. Not stated | - Anti-cancer treatment-related complications  - Morbidity | a. Unclear b. Unclear c. Unclear d. Unclear e. Low f. Unclear  g. High |
| Toogood, 1978  RCT | a. 26  b. Median 4 years (range 18 months-14 years 2 months  c. 69% male  d. ALL | a. Hb threshold 6.2-7.5 mmol/L  b. 13 patients  c. Not stated | a. Hb threshold 9.93-11.17 mmol/L  b. 13 patients  c. Not stated | - Anti-cancer treatment-related complications  - Morbidity | a. Unclear b. Unclear c. Unclear d. Unclear e. High f. Unclear  g. High |

**Table 2.** Characteristics of the non-RCT included studies (*n*=1).

| Study **Author, year Study type** | Population **a. No. of patients b. Age (years) c. Gender (% males) d. Diagnosis** | Intervention group **a. Intervention (including dosage) b. No. of patients c. Transfusion duration/dosage** | Control Group **a. Intervention (including dosage) b. No. of patients c. Transfusion time/dosage** | Included outcomes | Risk of bias assessment  **a. Selection bias**  **b. Attrition bias**  **c. Detection bias**  **d. Reporting bias**  **e. Confounding bias**  **f. Other bias** |
| --- | --- | --- | --- | --- | --- |
| Lightdale, 2012 Pre-post trial | a. 141  b. Pre 6 (IQR 2-12.3), post 6 (3-13)  c. Pre 43.9%, post 73.3% male  d. HSCT: hematological malignancies, lymphoma, solid tumor, non-malignant hematology, neuroblastoma | a. Hb threshold <4.3 mmol/L  b. 75 patients  c. Not stated | a. Hb threshold <5.6 mmol/L  b. 66 patients  c. Not stated | - Mortality - Admission to hospital - Costs | a. Low b. High c. Low^1^ d. High e. Low f. High |

^1^ Low considering that the included outcomes are not likely to be biased.

**Table 3.** Conclusions of evidence related to red blood cell transfusions in children and neonates with cancer

| **Conclusions of evidence** | **Quality of evidence** |
| --- | --- |
| Children with cancer - in general | |
| **Mortality**  100-days mortality: There is no significant difference in mortality between children with cancer who received RBC transfusion at a higher threshold (Hb of 5.6 mmol/L) or at a lower threshold (Hb of 4.3 mmol/L).  **Transfusion-related complications**  Incidence of VOD: There is no significant difference in incidence of VOD between children after SCT who received a RBC transfusion at a higher threshold (Hb of 7.5 mmol/L) in comparison to a lower threshold (Hb of 4.3 mmol/L).  **Anti-cancer treatment-related complications**  Deferred chemotherapy: Less delay of chemotherapy occurred in children with cancer who received a RBC transfusion at a higher threshold (Hb of 8.69-11.17 mmol/L) in comparison to patients with lower threshold (Hb of 6.2-7.5 mmol/L).  **Morbidity**  Incidence of infections: Inconclusive results, in one study less infections occurred in the higher RBC transfusion level (Hb of 8.69- 9.93 mmol/L versus 6.2-7.5 mmol/L) and in the other study there was no difference (Hb of 9.93-11.17 mmol/L versus 6.2-7.5 mmol/L).  **Admission to hospital**  Length of stay: There was no significant difference in length of stay between children with cancer who received RBC transfusion at a higher threshold (Hb of 5.6 mmol/L) and a lower threshold (Hb of 4.3 mmol/L).  **Costs**  Higher costs are associated with a RBC transfusion in children with cancer at a higher threshold (Hb of 5.6 mmol/L) in comparison to a lower threshold (Hb of 4.3 mmol/L). | ⨁◯◯◯ (1 study) VERY LOW  ⨁⨁◯◯ (1 study) LOW  ⨁⨁◯◯ (2 studies)  LOW  ⨁⨁◯◯ (1 study) LOW  ⨁⨁◯◯ (1 study) LOW  ⨁◯◯◯ (1 study) VERY LOW  ⨁◯◯◯ (1 study) VERY LOW |
