# Supplementary material for: Prophylactic red blood cell transfusions in children and neonates with cancer: An evidence-based clinical practice guideline
Source: Support Care Cancer. 2024 Nov 4;32(11):766. doi: 10.1007/s00520-024-08888-3 (PMC11534970; doi:10.1007/s00520-024-08888-3)
Supplement: Supplementary file 7 — Supplementary file7 (DOCX 81.6 KB) [file 520_2024_8888_MOESM7_ESM.docx]

**Supplemental materials S7: Overview of the included studies, conclusions of evidence, the evidence tables and the GRADE assessments**

**1. PROPHYLACTIC RED BLOOD CELL TRANSFUSION IN GENERAL
1.1 PROPHYLACTIC RED BLOOD CELL TRANSFUSION IN CHILDREN WITH CANCER**

**A. Discussion of the literature**

*A1: Discussion of the evidence*

A1.1: Evidence in pediatric oncology

Four pediatric oncology studies were included from the initial search (Robitaille, 2013; Smith, 1976; Toogood, 1978; Lightdale, 2012).

A1.2: Recommendations and evidence derived from guidelines

Two out of seven guidelines included recommendations for children with cancer. Valentine (2018) (33) advised an Hb threshold between 4.3 and 5.0 mmol/L based on 1 general pediatric study (15) and one general adult study (16). JPAC (2013) (41) recommended an Hb threshold of 4.3 mmol/L based on the same pediatric study (15).

Three out of seven guidelines had recommendations for children. These recommendations were based on both adult and pediatric studies. Valentine (2018) (33) advised an Hb threshold of 3.1 mmol/L in critically ill children or those at risk for critical illness. An Hb threshold higher than 4.3 mmol/L is not advised in critically ill children or those at risk for critical illness, who are hemodynamically stable. They did not make recommendations regarding Hb thresholds between 3.1 and 4.3 mmol/L due to lack of evidence (9, 15, 35, 46-48). New (2016) (44) advised an Hb threshold of 4.3 mmol/L in stable non-cyanotic patients and an Hb threshold higher than 4.3 mmol/L may be considered in unstable patients or symptomatic anemia (12, 15, 18, 43). NICE (2015) (43) has recommended an Hb threshold of 4.3 mmol/L as well (15, 30).

Two out of seven guidelines had recommendations for adults with cancer, however these recommendations were based merely on consensus and not on evidence. The JPAC (2013) (41) advised an Hb threshold between 5.0 and 5.6 mmol/L based on what most hospitals in the UK followed, but it is not based on evidence. CBO (2011) (42) stated that an Hb of 3.0 mmol/L is an absolute indication for a RBC transfusion (10). Based on consensus, it is advised to give a prophylactic RBC transfusion based on the patient’s cardiopulmonary compensation abilities and when there are no clear limited cardiopulmonary compensation options or risk factors, an Hb threshold between 3.5-4.5 may be used for children and adolescents under 25 years. In case of solid tumors, an Hb threshold of 6.0 mmol/L can be considered. For lymphocytic and myeloid leukemias, no recommendation could be made. In case of aplasia-inducing treatments it has been shown that a restrictive transfusion policy (4.4-5.5 mmol/L) compared to a more liberal policy (6 mmol/L) did not lead to more adverse patient outcomes (19).

*A2: Description of the included studies*

A2.1: Pediatric oncology

Four pediatric oncology studies were included (13, 14, 45, 46).

The characteristics of the included studies are stated in the evidence table below. The studies differ from each other in patient population and in methodology. The full tables of characteristics are stated in supplemental materials 2 and 3.

**Table 1.** Characteristics of the included studies regarding children with cancer.

| **Included studies** | | | | |
| --- | --- | --- | --- | --- |
| **Study** | **Population** | **Liberal threshold** | **Restrictive threshold** | **Outcomes** |
| *Robitaille (2013) RCT* | 6 children who received an allogeneic bone marrow transplantation after receiving myeloablative condition | - 7.5 mmol/L | - 4.3 mmol/L | - Transfusion-related complications* |
| *Lightdale (2012)*  *Pre-post study* | 141 children with different types of cancer | - 5.6 mmol/L | - 4.3 mmol/L | - Mortality  - Admission to hospital  - Costs |
| *Toogood (1978)*  *RCT* | 26 children with acute lymphocytic leukemia | - Between 9.9 and 11.2 mmol/L | - Between 6.2 and 7.5 mmol/L | - Anti-cancer treatment-related complications  - Morbidity |
| *Smith (1976)*  *RCT* | 27 children with different types of cancer | - Between 8.7 and 9.9 mmol/L | - Between 6.2 and 7.5 mmol/L | - Anti-cancer treatment-related complications  - Morbidity |

* This study was stopped for safety concerns when all patients in the experimental arm were diagnosed with veno-occlusive disease. The incidence of veno-occlusive disease was not statistically higher in the experimental arm, *p*=.05.

A2.2: Children in general

Five additional pediatric studies were included (9, 14, 47-49).

The characteristics of the included studies are stated in the evidence table below. The studies differ from each other in patient population and in methodology.

**Table 2.** Characteristics of the included studies regarding children.

| **Included studies** | | | | |
| --- | --- | --- | --- | --- |
| **Study** | **Population** | **Liberal threshold** | **Restrictive threshold** | **Outcomes** |
| *English (2002)*  *Retrospective and prospective cohort study* | 1516 severely anemic children divided into 1185 children who had malaria and 331 children with other diagnoses in Kenya | - 3.1 mmol/L | - 2.5 mmol/L  - 3.1 mmol/L with respiratory distress | - Mortality  - Morbidity  - Admission to hospital |
| *Akech (2008)*  *Prospective observational study* | 213 children who survived malaria | - 3.1 mmol/L | - 2.5 mmol/L  - 3.1 mmol/L with respiratory distress | - Mortality |
| *Lackritz (1992)*  *Observational study* | 683 children with severe anemia (Hb of 3.1 mmol/L) | - Transfusion (when they had an Hb of 2.4 mmol/L) | - No transfusion (when they had an Hb of 2.4 mmol/L | - Mortality |
| *Lackritz (1997)*  *Prospective cohort study* | 303 children with severe anemia | - Transfusion (when they had an Hb of 3.1 mmol/L) | - No transfusion (when they had an Hb of 3.1 mmol/L | - Mortality |
| *Lacroix (2007)*  *RCT* | 637 stable critically ill children | - 5.0 mmol/L | - 4.3 mmol/L | - Mortality  - Morbidity  - Admission to hospital |

A2.3: Adults

Six additional adult studies were included (8, 10-12, 15, 16).

The characteristics of the included studies are stated in the evidence table below. The studies differ from each other in patient population and in methodology.

**Table 3.** Characteristics of the included studies regarding adults.

| **Included studies** | | | | |
| --- | --- | --- | --- | --- |
| **Study** | **Population** | **Liberal threshold** | **Restrictive threshold** | **Outcomes** |
| *Carson (2002)*  *Retrospective study* | 300 adults with a postoperative Hb of 5.0 mmol/L | - Different Hb concentrations | | - Mortality |
| *Shander (2014)*  *Retrospective study* | 293 adults with postoperative Hb of 5.0 mmol/L | - Different Hb concentrations | | - Mortality |
| *Viele & Weskopf (1994)*  *MEDLINE search of case reports* | 61 reports of non-transfused Jehovah’s Witnesses with Hb concentrations of 5.0 mmol/L or hematocrits of 0.24 L/L | - Different Hb concentrations | | - Mortality |
| *Carson (2012)*  *Review of 31 studies* | 12587 adult patients | - 5.6 mmol/L | - 4.3 mmol/L | - Mortality  - Morbidity  - Admission to hospital  - Quality of life |
| *Hébert (1999)*  *RCT* | 838 critically ill patients with euvolemia | - 6.2 mmol/l | - 4.3 mmol/L | - Mortality  - Morbidity  - Admission to hospital |
| *Rohde (2014)*  *Review of 17 studies* | 7456 adult patients | - Between 5.6 and 8.4 mmol/L | - Between 4.0 and 6.8 mmol/L | - Morbidity |

A2.4: Excluded studies

| **Excluded studies** | |
| --- | --- |
| **Study** | **Reasons for exclusion** |
| *Lacroix (2012)* | This was a subanalysis and consisted of the same studydata as Lacroix (2007). However, the subanalyses are included in the section “Children with sepsis” and “Children with cancer and cardiac and/or pulmonary comorbidities”. |
| *Olupot-Olupot (2014)* | Not the right comparison (RBC volume instead of RBC thresholds). However, included in the section “Low or high-volume transfusions in children with cancer”. |
| *Cholette (2011)* | This study included children with cardiac comorbidities and thus was included in the section “Children with cancer and cardiac and/or pulmonary comorbidities”. |
| *Hébert & Carson (2014)* | This was an editorial. |
| *Hajjar (2010)* | This study included adults with cardiac comorbidities and thus was included in the section “Children with cancer and cardiac and/or pulmonary comorbidities”. |

**B. Results**

B1.1: Hb of 1.2 mmol/L versus Hb greater than 1.2 mmol/L

| Conclusion of evidence | Effect | Level of evidence / studies |
| --- | --- | --- |
| Children with cancer – Systematic literature search | | |
| No studies | - | - |
| Children non-cancer – Evidence cited in existing guidelines | | |
| No studies | - | - |
| Adults – Evidence cited in existing guidelines | | |
| Mortality | | |
| Significantly higher mortality in the group with an Hb of 1.2 mmol/L vs. greater than 1.2 mmol/L. | **RR 6.64 (95% CI 4.76 - 9.27)** | *Very low*** / Carson 2002 |

** The level of evidence is taken directly from the concerned guidelines *minus* 1 level for indirectness in this guideline.

B1.2: Hb of 1.9 mmol/L versus Hb greater than 1.9 mmol/L

| Conclusion of evidence | Effect | Level of evidence / studies |
| --- | --- | --- |
| Children with cancer – Systematic literature search | | |
| No studies | - | - |
| Children non-cancer – Evidence cited in existing guidelines | | |
| No studies | - | - |
| Adults – Evidence cited in existing guidelines | | |
| Mortality | | |
| Significantly higher mortality in the group with an Hb of 1.9 mmol/L vs. greater than 1.9 mmol/L. | **RR 5.20 (95% CI 3.13 - 8.65)**  **RR 6.83 (95% CI 2.78 - 16.81)**  *Pooled effect:* **RR 5.46 (95% CI 3.49 - 8.54)** | *Very low*** / Carson 2002  *Very low*** / Shander 2014 |

** The level of evidence is taken directly from the relevant guidelines *minus* 1 level for indirectness in this guideline.

B1.3: Hb of 2.5 mmol/L versus Hb greater than 2.5 mmol/L

| Conclusion of evidence | Effect | Level of evidence / studies |
| --- | --- | --- |
| Children with cancer – Systematic literature search | | |
| No studies | - | - |
| Children non-cancer – Evidence cited in existing guidelines | | |
| Mortality | | |
| Significantly higher mortality in the group with an Hb of 2.5 mmol/L vs. greater than 2.5 mmol/L in 2 studies, however 1 study reported no significant difference. | **RR 2.08 (95% CI 1.25 - 3.46)**  RR 1.12 (95% CI 0.32 - 3.94)  **RR 2.51 (95% CI 1.66 - 3.79)*** | *Very low*** / English 2002  *Very low*** / Akech 2008  *Very low*** / Lackritz 1992 |
| Adults – Evidence cited in existing guidelines | | |
| Mortality | | |
| Significantly higher mortality in the group with an Hb of 2.5 mmol/L vs. greater than 2.5 mmol/L in 1 study, however 1 study reported no significant difference. The pooled effect showed a significantly higher mortality in the group with an Hb of 2.5 mmol/L vs. greater than 2.5 mmol/L. | **RR 2.87 (95% CI 1.34 - 6.14)**  RR 2.82 (95% CI 0.93 - 8.59)  *Pooled effect:* **RR 2.85 (95% CI 1.52 - 5.35)** | *Very low*** / Carson 2002  *Very low*** / Shander 2014 |

* Results could not be pooled due to different study populations or outcome measures.

** The level of evidence is taken directly from the relevant guidelines *minus* 1 level for indirectness in this guideline.

B1.4: Hb of 3.1 mmol/L versus Hb greater than 3.1 mmol/L

| Conclusion of evidence | Effect | Level of evidence / studies |
| --- | --- | --- |
| Children with cancer – Systematic literature search | | |
| No studies | - | - |
| Children non-cancer – Evidence cited in existing guidelines | | |
| Mortality | | |
| Significantly higher mortality in the group with an Hb of 3.1 mmol/L vs. greater than 3.1 mmol/L. | **RR 1.93 (95% CI 1.36 - 2.74)** | *Very low*** / Lackritz 1997 |
| Adults – Evidence cited in existing guidelines | | |
| Mortality | | |
| Significantly higher mortality in the group with an Hb of 3.1 mmol/L vs. greater than 3.1 mmol/L. | **RR 3.87 (95% CI 1.56 - 9.58)**  **RR 7.18 (95% CI 3.32 – 15.54)**  *Pooled effect:* **RR 5.50 (95% CI 3.08 – 9.83)**  **23 of 50 deaths primarily due to anemia with Hb <3.1 mmol/L** | *Very low *** / Shander 2014  *Very low*** / Carson 2002  *Very low *** / Viele 1994 |

** The level of evidence is taken directly from the relevant guidelines *minus* 1 level for indirectness in this guideline.

B1.5: Hb of 3.7 mmol/L versus Hb greater than 3.7 mmol/L

| Conclusion of evidence | Effect | Level of evidence / studies |
| --- | --- | --- |
| Children with cancer – Systematic literature search | | |
| No studies | - | - |
| Children non-cancer – Evidence cited in existing guidelines | | |
| No studies | - | - |
| Adults – Evidence cited in existing guidelines | | |
| Mortality | | |
| Higher mortality in the group with an Hb of 3.7 mmol/L vs. an Hb greater than 3.7 mmol/L in one study. No significant effect in another study. However, the pooled effect was significant. | **RR 5.46 (95% CI 1.81 - 16.46)**  RR 2.87 (95% CI 0.86 - 9.53)  *Pooled effect:* **RR 4.01 (95% CI 1.80 - 8.95)** | *Very low*** / Shander 2014  *Very low*** / Carson 2002 |

** The level of evidence is taken directly from the relevant guidelines *minus* 1 level for indirectness in this guideline.

B1.6: Hb of 4.3 mmol/L versus Hb greater than 4.3

| Conclusion of evidence | Effect | Level of evidence / studies |
| --- | --- | --- |
| Children with cancer – Systematic literature search | | |
| Mortality | | |
| No significant difference in the group with an Hb of 4.3 mmol/L vs. greater than 4.3 mmol/L. | RR 0.67 (95% CI 0.35 - 1.28) | *Very low* / Lightdale 2012 |
| Transfusion-related complications | | |
| No significant difference in the group with an Hb of 4.3 mmol/L vs. greater than 4.3 mmol/L. | RR 7.00 (95% CI 0.51 - 96.06) | *Low* / Robitaille 2013 |
| Hospital admission | | |
| No significant difference in the group with an Hb of 4.3 mmol/L vs. greater than 4.3 mmol/L. | Not significant (no effect measure reported) | *Very low* / Lightdale 2012 |
| Costs | | |
| Significantly lower costs in the group with an Hb of 4.3 mmol/L vs. greater than 4.3 mmol/L. | **P=0.004 (no effect measure reported)** | *Very low* / Lightdale 2012 |
| Children non-cancer – Evidence cited in existing guidelines | | |
| Mortality | | |
| No significant differences in the group with an Hb of 4.3 mmol/L vs. greater than 4.3 mmol/L. | RR 0.99 (95% CI 0.48 - 2.04) | *Low*** / Lacroix 2007 |
| Morbidity | | |
| No significant differences in the group with an Hb of 4.3 mmol/L vs. greater than 4.3 mmol/L. | RR 0.97 (95% CI 0.63 - 1.47) | *Low*** / Lacroix 2007 |
| Hospital admission | | |
| No significant differences in the group with an Hb of 4.3 mmol/L vs. greater than 4.3 mmol/L. | MD -0.46 (95% CI -0.70 - 1.70) | *Low*** / Lacroix 2007 |
| Adults – Evidence cited in existing guidelines | | |
| Mortality | | |
| One study reported significantly less mortality in the group with an Hb of 4.3 mmol/L. 1 study reported significantly more mortality in the group with an Hb of 4.3 mmol/L. Others reported no significant differences in group with an Hb of 4.3 mmol/L vs. greater than 4.3 mmol/L. | **RR 0.75 (95% CI 0.59 - 0.96)**  RR 0.79 (95% CI 0.63 - 1.00)  **RR 19.30 (95% CI 1.09 - 342.66)**  RR 3.44 (95% CI 0.59 - 20.04)* | *Low*** / Carson 2012  *Very low*** / Hébert 1999  *Very low*** / Carson 2002  *Very low*** / Shander 2014 |
| Quality of life | | |
| No significant differences in the group with an Hb of 4.3 mmol/L vs. greater than 4.3 mmol/L. | Not significant (no effect measure reported) | *Low*** / Carson 2012 |
| Morbidity | | |
| Significantly less infections in the group with an Hb of 4.3 mmol/L vs. greater than 4.3 mmol/L.  No significant differences regarding other morbidities in the group with an Hb of 4.3 mmol/L vs. greater than 4.3 mmol/L. | **RR 0.81 (95% CI 0.67 - 0.98)**  **RR 0.83 (95% CI 0.72 - 0.96)***  RR 1.23 (95% CI 0.67 - 2.26) | *Low*** / Carson 2012  *Low*** / Rohde 2014  *Very low*** / Hébert 1999 |
| Hospital admission | | |
| No significant differences in the group with an Hb of 4.3 mmol/L vs. greater than 4.3 mmol/L. | MD -0.70 (95% CI -3.37 - 1.97)  MD 0.11 (95% CI -0.16 - 0.13) | *Very low*** / Hébert 1999  *Low*** / Carson 2012 |

* Results could not be pooled due to different study populations or outcome measures.

** The level of evidence is taken directly from the relevant guidelines *minus* 1 level for indirectness in this guideline.

**C. Conclusions**

| **VERY LOW QUALITY OF EVIDENCE (GRADE)** | Significantly more mortality in the group with *a hemoglobin (Hb) of 3.1 mmol/L versus a hemoglobin (Hb) greater than 3.1 mmol/L* in 4 studies.  Sources (*Lackritz, 1997; Shander, 2014; Carson, 2002; Viele & Weiskopf, 1994*) |
| --- | --- |

| **VERY LOW QUALITY OF EVIDENCE (GRADE)** | Significantly more mortality in the group with *a hemoglobin (Hb) of 3.7 mmol/L versus a hemoglobin (Hb) greater than 3.7 mmol/L* in 2 studies.  Sources (*Shander, 2014; Carson, 2002*) |
| --- | --- |

| **VERY LOW QUALITY OF EVIDENCE (GRADE)** | There is no increased risk for mortality, morbidity, and transfusion-related complications with *a hemoglobin (Hb) of 4.3 mmol/L versus a hemoglobin (Hb) greater than 4.3 mmol/L* in 4 out of 6 studies. However, 1 study reported significantly less mortality and 1 study reported significantly more mortality. Moreover, there were two studies who reported significantly less infections with *a hemoglobin (Hb) of 4.3 mmol/L versus a hemoglobin (Hb) greater than 4.3 mmol/L.* In addition, there are no studies reporting any other significant potential benefit from a hemoglobin (Hb) greater than 4.3 mmol/L versus a hemoglobin (Hb) of 4.3 mmol/L (5 studies).  Sources (*Lightdale, 2012; Robitaille, 2013; Lacroix, 2012; Carson, 2002; Carson, 2012; Hébert, 1999; Rohde, 2014; Shander, 2014*) |
| --- | --- |

**1.2 PROPHYLACTIC RED BLOOD CELL TRANSFUSION IN NEONATES WITH CANCER**

**A. Discussion of the literature**

*A1: Discussion of the evidence*

A1.1: Evidence in pediatric oncology

No pediatric oncology studies were included.

A1.2: Recommendations and evidence derived from guidelines

Four out of seven guidelines included recommendations for neonates. The Dutch Association of Medical Specialists (22) has recommended a hemoglobin (Hb) threshold of 6.5 mmol/L in neonates less than 1 week old, between 2-3 weeks old an Hb threshold of 5.5 mmol/L and an Hb threshold of 4.5 mmol/L in neonates older than 3 weeks (49-53). JPAC (2013) (41) has based their recommendations on the British Committee for Standards in Haematology (BCSH) (2016) (44) and has recommended Hb threshold of 6.2 mmol/L in neonates less than 1 week old. For neonates older than 2 weeks it is recommended to maintain the Hb between 4.7 and 5.3 mmol/L depending on their clinical situation (50-54).

*A2: Description of the included studies*

A2.1: Neonates with cancer

There were no primary pediatric oncology studies included from the systematic literature search.

A2.2: Neonates in general

There were six studies included from the additional literature review (49-53, 55, 56).

The characteristics of the included studies are stated in the evidence table below. The studies differ from each other in patient population (gestational age and birth weight) and in methodology.

**Table 1.** Characteristics of the included studies regarding neonates with cancer.

| **Included studies** | | | | |
| --- | --- | --- | --- | --- |
| **Study** | **Population** | **Liberal threshold** | **Restrictive threshold** | **Outcomes** |
| Bell (2005) *RCT* | 103 neonates with very low birth weight (500 to 1300 grams) | - Neonates on ventilation 8.4 mmol/L  - Neonates on oxygen/CPAP 6.9 mmol/L  - Neonates without respiratory support 5.4 mmol/L | - Neonates on ventilation 6.2 mmol/L  - Neonates on oxygen/CPAP 5.0 mmol/L  - Neonates without respiratory support 4.4 mmol/L | - Mortality  - Morbidity  - Costs |
| Brooks (1999)  *RCT* | 50 neonates with very low birthweight (<1250 grams) | - Hematocrit of 0.40 L/L | - Asymptomatic: Hematocrit of 0.20 L/L + reticulocyte count 0.1x10e9/L  - Symptomatic: Hematocrit of 0.30 L/L | - Mortality  - Morbidity |
| Chen (2009)  *RCT* | 36 neonates with very low birth weight (<1500 grams) | - Neonates on ventilation 9.1 mmol/L  - Neonates on CPAP 8.3 mmol/L  - Neonates without respiratory support 6.2 mmol/L | - Neonates on ventilation 7.2 mmol/L  - Neonates on CPAP 6.2 mmol/L  - Neonates without respiratory support 4.5 mmol/L | - Mortality  - Morbidity  - Costs |
| Connelly (1999)  *Prospective study* | 24 neonates with very low birth weight (<1500 grams) | - First week of life 8.1 mmol/L  - Second week of life with respiratory support 6.8 mmol/L  - Third week of life 5.0 mmol/L | - First week of life 6.8 mmol/L  - Second week of life with respiratory support 5.6 mmol/L  - Third week of life 5.0 mmol/L | - Mortality  - Morbidity  - Costs |
| Mukhopadhyay (2004)  *RCT* | 38 preterm neonates weighing between 1000 and 1800 grams | - Hematocrit threshold of 0.40 L/L | - Hematocrit threshold of 0.30 L/L | - Mortality |
| Whyte & Kirpalani (2006)  *RCT* | 451 neonates with very low birth weight (<1000 gram) | - Neonates without respiratory support in week 1 7.5 mmol/L  - Neonates without respiratory support in week 2 6.2 mmol/L  - Neonates without respiratory support in week 3 5.3 mmol/L  - Neonates on respiratory support in week 1 8.4 mmol/L  - Neonates on respiratory support in week 2 7.5 mmol/L  - Neonates on respiratory support in week 3 6.2 mmol/L | - Neonates without respiratory support in week 1 6.2 mmol/L  - Neonates without respiratory support in week 2 5.3 mmol/L  - Neonates without respiratory support in week 3 4.7 mmol/L  - Neonates on respiratory support in week 1 7.1 mmol/L  - Neonates on respiratory support in week 2 6.2 mmol/L  - Neonates on respiratory support in week 3 5.3 mmol/L | - Mortality  - Morbidity  - Costs |

A2.3: Excluded studies

| **Excluded studies** | |
| --- | --- |
| **Study** | **Reasons for exclusion** |
| *Whyte & Kirpalani (2011)* | This was a review, including the following studies: Connelly (1999), Bell (2005), Chen (2009), and Whyte & Kirpalani (2006) and were all included. |
| *Venkatesh (2012)* | This was a review, including the following studies: Bell (2005), Chen (2009), Whyte & Kirpalani (2006), Brooks (1999), Mukhopadhya (2004), and Ransome (1989) and were all included except Ransome (1989) considering that this article was not found. |

**B. Results**

*B1: Neonates in general*
B1.1: Hb of 4.3 mmol/L versus Hb greater than 4.3 mmol/L in neonates

| Conclusion of evidence | Effect | Level of evidence / studies |
| --- | --- | --- |
| Children with cancer – Systematic literature search | | |
| No studies | - | - |
| Children non-cancer – Evidence cited in existing guidelines | | |
| Mortality | | |
| There was no significant difference regarding mortality when comparing an Hb of 4.3 mmol/L vs. greater than 4.3 mmol/L in neonates. | RR 0.52 (95% CI 0.05 - 5.56)  None died (not estimable) | *Low** / Bell 2005  *Low** / Brooks 1999 |
| Morbidity | | |
| There was no significant difference regarding morbidity when comparing an Hb of 4.3 mmol/L vs. greater than 4.3 mmol/L in neonates. | Not significant (no effect measure reported)  Not significant (no effect measure reported) | *Low** / Bell 2005  *Low** / Brooks 1999 |
| Costs | | |
| There was a significant difference regarding costs when comparing an Hb of 4.3 mmol/L vs. greater than 4.3 mmol/L in neonates. | **MD -1.10 (95% CI -2.10 - -0.10).** | *Low** / Bell 2005 |
| Adults – Evidence cited in existing guidelines | | |
| No studies | - | - |

* The level of evidence is taken directly from the relevant guidelines *minus* 1 level for indirectness in this guideline.

B.1.2: Hb of 5.0 mmol/L versus Hb greater than 5.0 mmol/L in neonates

| Conclusion of evidence | Effect | Level of evidence / studies |
| --- | --- | --- |
| Children with cancer – Systematic literature search | | |
| No studies | - | - |
| Children non-cancer – Evidence cited in existing guidelines | | |
| Mortality | | |
| There was no significant difference regarding mortality when comparing an Hb of 5.0 mmol/L vs. greater than 5.0 mmol/L in neonates. | RR 1.79 (95% CI 0.18 - 18.02) | *Low** / Chen 2009 |
| Morbidity | | |
| There was no significant difference regarding morbidity when comparing an Hb of 5.0 mmol/L vs. greater than 5.0 mmol/L in neonates. | Not significant (no effect measure reported) | *Low** / Chen 2009 |
| Costs | | |
| There was a significant difference regarding costs when comparing an Hb of 5.0 mmol/L vs. greater than 5.0 mmol/L in neonates. | MD -1.00 (95% CI -2.49 - 0.49) | *Low** / Chen 2009 |
| Adults – Evidence cited in existing guidelines | | |
| No studies | - | - |

* The level of evidence is taken directly from the relevant guidelines *minus* 1 level for indirectness in this guideline.

B1.3: Hb of 6.2 mmol/L versus Hb greater than 6.2 mmol/L in neonates

| Conclusion of evidence | Effect | Level of evidence / studies |
| --- | --- | --- |
| Children with cancer – Systematic literature search | | |
| No studies | - | - |
| Children non-cancer – Evidence cited in existing guidelines | | |
| Mortality | | |
| There was no significant difference regarding mortality when comparing an Hb of 6.2 mmol/L vs. greater than 6.2 mmol/L in neonates. | RR 3.50 (95% CI 0.62 - 1.18) | *Low** Mukhopadyay 2004 |
| Adults – Evidence cited in existing guidelines | | |
| No studies | - | - |

* The level of evidence is taken directly from the relevant guidelines *minus* 1 level for indirectness in this guideline.

*B2: Neonates in first week of life*

B2.1: Hb of 6.2 mmol/L versus Hb greater than 6.2 mmol/L in neonates in the first week of life

| Conclusion of evidence | Effect | Level of evidence / studies |
| --- | --- | --- |
| Children with cancer – Systematic literature search | | |
| No studies | - | - |
| Children non-cancer – Evidence cited in existing guidelines | | |
| Mortality | | |
| There was no significant difference regarding mortality when comparing an Hb of 6.20 mmol/L vs. greater than 6.2 mmol/L in neonates in the first week of life. | RR 1.23 (95% CI 0.84 - 1.79) | *Low** / Whyte & Kirpalani 2006 |
| Morbidity | | |
| There was no significant difference regarding morbidity when comparing an Hb of 6.20 mmol/L vs. greater than 6.2 mmol/L in neonates in the first week of life.  There was a significant difference regarding retinopathy of prematurity when comparing an Hb of 6.20 mmol/L vs. greater than 6.2 mmol/L in neonates in the first week of life. | Not significant (no effect measure reported)  **RR 0.79 (95% CI 0.66 - 0.95)** | *Low** / Whyte & Kirpalani 2006  *Low** / Whyte & Kirpalani 2006 |
| Costs | | |
| There was no significant difference regarding costs when comparing an Hb of 6.20 mmol/L vs. greater than 6.2 mmol/L in neonates in the first week of life. | MD -0.80 (95% CI -1.65 - 0.05) | *Low** / Whyte & Kirpalani 2006 |
| Adults – Evidence cited in existing guidelines | | |
| No studies | - | - |

* The level of evidence is taken directly from the relevant guidelines *minus* 1 level for indirectness in this guideline.

B2.2: Hb of 6.8 mmol/L versus Hb greater than 6.8 mmol/L in neonates in the first week of life

| Conclusion of evidence | Effect | Level of evidence / studies |
| --- | --- | --- |
| Children with cancer – Systematic literature search | | |
| No studies | - | - |
| Children non-cancer – Evidence cited in existing guidelines | | |
| Mortality | | |
| There was no significant difference regarding mortality when comparing an Hb of 6.8 mmol/L vs. greater than 6.8 mmol/L in neonates in the first week of life. | Not significant (no effect measure reported) | *Low** / Connelly 1999 |
| Morbidity | | |
| There was no significant difference regarding morbidity when comparing an Hb of 6.8 mmol/L vs. greater than 6.8 mmol/L in neonates in the first week of life. | Not significant (no effect measure reported) | *Low** / Connelly 1999 |
| Costs | | |
| There was a significant difference regarding costs when comparing an Hb of 6.8 mmol/L vs. greater than 6.8 mmol/L in neonates in the first week of life. | **MD -2.90 (95% CI -4.94 - -0.86)** | *Low** / Connelly 1999 |
| Adults – Evidence cited in existing guidelines | | |
| No studies | - | - |

* The level of evidence is taken directly from the relevant guidelines *minus* 1 level for indirectness in this guideline.

*B3: Neonates on the second week of life*

B3.1: Hb of 5.3 mmol/L versus Hb greater than 5.3 mmol/L in neonates in the second week of life

| Conclusion of evidence | Effect | Level of evidence / studies |
| --- | --- | --- |
| Children with cancer – Systematic literature search | | |
| No studies | - | - |
| Children non-cancer – Evidence cited in existing guidelines | | |
| Mortality | | |
| There was no significant difference regarding mortality when comparing an Hb of 5.3 mmol/L vs. greater than 5.3 mmol/L in neonates in the second week of life. | RR 1.23 (95% CI 0.84 - 1.79) | *Low** / Whyte & Kirpalani 2006 |
| Morbidity | | |
| There was no significant difference regarding morbidity when comparing an Hb of 5.3 mmol/L vs. greater than 5.3 mmol/L in neonates in the second week of life.  There was a significant difference regarding retinopathy of prematurity when comparing an Hb of 5.3 mmol/L vs. greater than 5.3 mmol/L in neonates in the second week of life. | Not significant (no effect measure reported)  **RR 0.79 (95% CI 0.66 - 0.95)** | *Low** / Whyte & Kirpalani 2006  *Low** / Whyte & Kirpalani 2006 |
| Costs | | |
| There was no significant difference regarding costs when comparing an Hb of 5.3 mmol/L vs. greater than 5.3 mmol/L in neonates in the second week of life. | MD -0.80 (95% CI -1.65 - 0.05) | *Low** / Whyte & Kirpalani 2006 |
| Adults – Evidence cited in existing guidelines | | |
| No studies | - | - |

* The level of evidence is taken directly from the relevant guidelines *minus* 1 level for indirectness in this guideline.

*B4. Neonates on the third week of life*

B4.1: Hb of 4.7 mmol/L versus Hb greater than 4.7 mmol/L in the third week of life

| Conclusion of evidence | Effect | Level of evidence / studies |
| --- | --- | --- |
| Children with cancer – Systematic literature search | | |
| No studies | - | - |
| Children non-cancer – Evidence cited in existing guidelines | | |
| Mortality | | |
| There was no significant difference regarding mortality when comparing an Hb of 4.7 mmol/L vs. greater than 4.7 mmol/L in neonates in the third week of life. | RR 1.23 (95% CI 0.84 - 1.79) | *Low** / Whyte & Kirpalani 2006 |
| Morbidity | | |
| There was no significant difference regarding morbidity when comparing an Hb of 4.7 mmol/L vs. greater than 4.7 mmol/L in neonates in the third week of life.  There was a significant difference regarding retinopathy of prematurity when comparing an Hb of 4.7 mmol/L vs. greater than 4.7 mmol/L in neonates in the third week of life. | Not significant (no effect measure reported)  **RR 0.79 (95% CI 0.66 - 0.95)** | *Low** / Whyte & Kirpalani 2006  *Low** / Whyte & Kirpalani 2006 |
| Costs | | |
| There was no significant difference regarding costs when comparing an Hb of 4.7 mmol/L vs. greater than 4.7 mmol/L in neonates in the third week of life. | MD -0.80 (95% CI -1.65 - 0.05) | *Low** / Whyte & Kirpalani 2006 |
| Adults – Evidence cited in existing guidelines | | |
| No studies | - | - |

* The level of evidence is taken directly from the relevant guidelines *minus* 1 level for indirectness in this guideline.

**C. Conclusions**

No conclusions were formulated.

**2. PROPHYLACTIC RED BLOOD CELL TRANSFUSION - SEPSIS**

**2.1 PROPHYLACTIC RED BLOOD CELL TRANSFUSION IN CHILDREN WITH CANCER DURING SEPSIS**

**A. Discussion of the literature**

*A1: Discussion of the evidence*

A1.1: Evidence in pediatric oncology

No pediatric oncology studies included children with sepsis.

A1.2: Recommendations and evidence derived from guidelines

Only one out of seven guidelines formulated recommendations for children with sepsis stating that a hemoglobin (Hb) threshold of 4.3 mmol/L can be safely applied in clinically stable children; however, in clinically unstable children a higher threshold may be suggested (15, 22, 29).

*A2: Description of the included studies*

A2.1: Pediatric oncology

There were no primary pediatric oncology studies included from the systematic literature search.

A2.2: Children in general

One pediatric study was included from the additional literature review (18). The characteristics of the included study are stated in the evidence table below.

**Table 1.** Characteristics of the included studies regarding children.

| **Included studies** | | | | |
| --- | --- | --- | --- | --- |
| **Study** | **Population** | **Liberal threshold** | **Restrictive threshold** | **Outcomes** |
| *Lacroix (2012)*  *RCT* | 137 stable critically ill children with sepsis | - 5.0 mmol/L | - 4.3 mmol/L | - Mortality  - Morbidity  - Admission to hospital |

A2.3: Adults

One adult study was included from the additional literature review (23). The characteristics of the included study are stated in the evidence table below.

**Table 2.** Characteristics of the included studies regarding adults.

| **Included studies** | | | | |
| --- | --- | --- | --- | --- |
| **Study** | **Population** | **Liberal threshold** | **Restrictive threshold** | **Outcomes** |
| *Holst (2014)*  *RCT* | 998 adult patients with sepsis | - 5.6 mmol/L | - 4.3 mmol/L | - Mortality  - Morbidity |

C2.4: Excluded studies

| **Excluded studies** | |
| --- | --- |
| **Study** | **Reasons for exclusion** |
| *Lacroix (2007)* | This study included children in general. For this section the subanalysis of Lacroix (2012) was included. |

**B. Results**

B1.1: Hb of 4.3 mmol/L versus Hb greater than 4.3

| Conclusion of evidence | Effect | Level of evidence / studies |
| --- | --- | --- |
| Children with cancer – Systematic literature search | | |
| No studies | - | - |
| Children non-cancer – Evidence cited in existing guidelines | | |
| Mortality | | |
| No significant differences in the group with an Hb of 4.3 mmol/L vs. greater than 4.3 mmol/L. | RR 3.45 (95% CI 0.74 - 16.02) | *Low** / Lacroix 2012 |
| Morbidity | | |
| No significant differences in the group with an Hb of 4.3 mmol/L vs. greater than 4.3 mmol/L. | RR 0.99 (95% CI 0.49-1.97) | *Low** / Lacroix 2012 |
| Hospital admission | | |
| No significant differences in the group with an Hb of 4.3 mmol/L vs. greater than 4.3 mmol/L. | Not significant (no effect measure reported) | *Low** / Lacroix 2012 |
| Adults with sepsis – Evidence cited in existing guidelines | | |
| Mortality | | |
| No significant differences in the group with an Hb of 4.3 mmol/L vs. greater than 4.3 mmol/L. | RR 0.96 (95% CI 0.83 - 1.10) | *Very low** / Holst 2014 |
| Morbidity | | |
| No significant differences in the group with an Hb of 4.3 mmol/L vs. greater than 4.3 mmol/L. | RR 0.33 (95% CI 0.01 - 8.18) | *Very low** / Holst 2014 |

* The level of evidence is taken directly from the relevant guidelines *minus* 1 level for indirectness in this guideline.

**C. Conclusions**

| **VERY LOW QUALITY OF EVIDENCE (GRADE)** | There were no significant differences regarding mortality, morbidity, and admission to hospital with *a hemoglobin (Hb) of 4.3 mmol/L versus a hemoglobin (Hb) greater than 4.3 mmol/L* in 2 studies.  Sources (*Lacroix, 2012; Holst, 2014*) |
| --- | --- |

**2.2 PROPHYLACTIC RED BLOOD CELL TRANSFUSION IN NEONATES WITH CANCER DURING SEPSIS
A: Discussion of the literature**

*A1: Discussion of the evidence*

A1.1: Evidence in pediatric oncology

No pediatric oncology studies included neonates.

A1.2: Recommendations and evidence derived from guidelines

No guidelines included neonates.

*A2: Description of the included studies*

There were no primary pediatric oncology studies included from the systematic literature search and no additional studies from the additional literature review.

**B. Results**

As no studies were included, no results were presented.

**C. Conclusions**

As no studies were included, no conclusions were formulated.

**3 PROPHYLACTIC RED BLOOD CELL TRANSFUSION - RADIOTHERAPY**

**3.1 PROPHYLACTIC RED BLOOD CELL TRANSFUSION IN CHILDREN WHO UNDERGO RADIOTHERAPY
A. Discussion of the literature**

*A1: Discussion of the evidence*

A1.1: Evidence in pediatric oncology

No pediatric oncology studies included children with cancer who undergo radiotherapy.

A1.2: Recommendations and evidence derived from guidelines

One guideline was found from the National Blood Authority (2012) (29) in which it was stated that the same hemoglobin (Hb) threshold should be used in patients with cancer who undergo radiotherapy (not further specified). This was based on consensus.

*A2: Description of the included studies*

There were no primary pediatric oncology studies included from the systematic literature search and no additional studies from the additional literature review. However, studies have demonstrated that low pretreatment Hb levels serve as a robust prognostic indicator for poor disease control and survival (24, 25). Conversely, a study involving 414 adult patients with Head and Neck Squamous Cell Carcinoma (HNSCC) investigated the impact of RBC transfusions on patients with “low” pre-irradiation Hb values (females Hb of 8.07 mmol/L, males Hb of 9.0 mmol/L). The study showed that the transfusions prior to and during the radiotherapy treatment did not improve the poor prognosis in patients with low Hb values (26). In addition, other RCTs have also failed to show the effectiveness of transfusion strategies and the use of erythropoietin was found to be counterproductive (27). In contrast, a study explored the potential of ARCON, a treatment involving the administration of a gas mixture carbogen and taking nicotinamide pills, in reversing the poor outcomes associated with anemia in cancer patients. The study aimed to elucidate why transfusions do not influence outcomes and proposed that RBC transfusions stimulate inflammatory and immunosuppressive pathways. Erythropoietin was found to decrease tissue oxygenation due to increased viscosity resulting from elevated Hb levels, and the presence of erythropoietin receptors on tumor cell membranes was observed to stimulate tumor growth. Animal studies indicated that correcting anemia through blood transfusions initially increased tumor radiosensitivity by improving tumor cell oxygenation. However, this effect was transient and dissipated within 24 hours. It is thought that tumors that are chronically exposed to higher oxygen levels, such as those associated with normal Hb levels, eventually adapt and exhibit increased proliferation and will outgrow their oxygen supply. In the case of ARCON, the duration of increased oxygenation is short (10-15 minutes) and prevents adaptation from occurring (28).

**B. Results**

As no studies were included, no results were presented.

**C. Conclusions**

As no studies were included, no conclusions were formulated.

**3.2 PROPHYLACTIC RED BLOOD CELL TRANSFUSION IN NEONATES WHO UNDERGO RADIOTHERAPY
A. Discussion of the literature**

*A1: Discussion of the evidence*

A1.1: Evidence in pediatric oncology

No pediatric oncology studies included neonates.

A1.2: Recommendations and evidence derived from guidelines

One guideline was found from the National Blood Authority (2012) (43) and they state that the same hemoglobin (Hb) threshold should be used in patients with cancer who undergo radiotherapy (not further specified). This was based on consensus.

*A2: Description of the included studies*

There were no primary pediatric oncology studies included from the systematic literature search and no additional studies from the additional literature review. However, studies have demonstrated that low pretreatment Hb levels serve as a robust prognostic indicator for poor disease control and survival (24, 25). Conversely, a study involving 414 adult patients with Head and Neck Squamous Cell Carcinoma (HNSCC) investigated the impact of RBC transfusions on patients with “low” pre-irradiation Hb values (females Hb of 8.07 mmol/L, males Hb of 9.0 mmol/L). The study showed that the transfusions prior to and during the radiotherapy treatment did not improve the poor prognosis in patients with low Hb values (26). In addition, other RCTs have also failed to show the effectiveness of transfusion strategies and the use of erythropoietin was found to be counterproductive (27). In contrast, a study explored the potential of ARCON, a treatment involving the administration of a gas mixture carbogen and taking nicotinamide pills, in reversing the poor outcomes associated with anemia in cancer patients. The study aimed to elucidate why transfusions do not influence outcomes and proposed that RBC transfusions stimulate inflammatory and immunosuppressive pathways. Erythropoietin was found to decrease tissue oxygenation due to increased viscosity resulting from elevated Hb levels, and the presence of erythropoietin receptors on tumor cell membranes was observed to stimulate tumor growth. Animal studies indicated that correcting anemia through blood transfusions initially increased tumor radiosensitivity by improving tumor cell oxygenation. However, this effect was transient and dissipated within 24 hours. It is thought that tumors that are chronically exposed to higher oxygen levels, such as those associated with normal Hb levels, eventually adapt and exhibit increased proliferation and will outgrow their oxygen supply. In the case of ARCON, the duration of increased oxygenation is short (10-15 minutes) and prevents adaptation from occurring (27)

**B. Results**

As no studies were included, no results were presented.

**C. Conclusions**

As no studies were included, no conclusions were formulated.

**4. PROPHYLACTIC RED BLOOD CELL TRANSFUSION - CARDIAC AND PULMONARY COMORBIDITIES**

**4.1 PROPHYLACTIC RED BLOOD CELL TRANSFUSION IN CHILDREN WITH CANCER WITH CARDIAC AND/OR PULMONARY COMORBIDITIES**

**A. Discussion of the literature**

*A1: Discussion of the evidence*

A1.1: Evidence in pediatric oncology

No pediatric oncology studies were included.

A1.2: Recommendations and evidence derived from guidelines

Three out of seven guidelines included recommendations for children with cardiac and pulmonary comorbidities. These recommendations are distinguished in children with acute respiratory failure and children with acquired and congenital heart disease.

Only Valentine (2018) (33) included recommendations for children with respiratory failure, advising a hemoglobin (Hb) threshold of 3.1 mmol/L. They do not advise an Hb threshold higher than 4.3 mmol/L in critically ill children with respiratory failure without severe acute hypoxemia, chronic cyanotic conditions, or hemolytic anemia. There was not enough evidence to create a recommendation regarding RBC transfusion thresholds between 3.1 and 4.3 mmol/L. A recommendation could not be made for critically ill children with respiratory failure and severe hypoxemia. These recommendations were based on 7 studies in both children and adults (8, 10, 11, 15, 46, 47, 57).

Valentine (2018) (33) and the BCSH (2016) (42) included recommendations for children with acquired and congenital heart disease. Valentine (2018) (33) advises in hemodynamically stable critically ill children with uncorrected congenital heart disease an Hb threshold between 4.3 mmol/L and 5.6 mmol/L depending on the cardiopulmonary reserve. There is not enough evidence to create recommendations for children with right or left ventricular myocardial dysfunction (acquired or congenital) or for children with a structurally normal heart and idiopathic or acquired pulmonary hypertension (mean pulmonary arterial pressure >25 mmHg with normal pulmonary capillary wedge pressure) (15). The BCSH (2016) (42) advises an Hb threshold of 4.3 mmol/L in stable children with non-cyanotic heart disease. There is insufficient evidence to make a recommendation for pre-transfusion Hb thresholds in pediatric hematology/oncology patients and those undergoing stem cell transplantation and for children with cyanotic heart disease (12, 15, 16, 18, 20, 41, 58).

*A2: Description of the included studies*

A2.1: Pediatric oncology

There were no primary pediatric oncology studies included from the systematic literature search.

A2.2: Children in general

Five pediatric studies were included from the additional literature review (18, 28, 30, 46, 57).

The characteristics of the included studies are stated in the evidence table below. The studies differ from each other in patient population and in methodology.

**Table 1.** Characteristics of the included studies regarding children.

| **Included studies** | | | | |
| --- | --- | --- | --- | --- |
| **Study** | **Population** | **Liberal threshold** | **Restrictive threshold** | **Outcomes** |
| *Lacroix (2012)*  *Subananalysis of Lacroix (2007)* | 400 stable critically ill children with respiratory distress | - 5.0 mmol/L | - 4.3 mmol/L | - Mortality  - Morbidity  - Admission to hospital |
| *Cholette (2011)*  *RCT* | 60 infants and children with variations of single-ventricle physiology presenting for cavopulmonary connection | - 8.1 mmol/L | - 5.6 mmol/L | - Mortality  - Admission to hospital |
| *Willems (2010)*  *Subananalysis of Lacroix (2007)* | 125 stable critically ill children post cardiac surgery (noncyanotic) | - 5.0 mmol/L | - 4.3 mmol/L | - Mortality  - Morbidity  - Admission to hospital |
| *Marsh (1995)*  *Prospective study* | 1844 children with malaria and an Hb below 3.1 mmol/L with respiratory distress | - | - | - Mortality |
| *English (2002)*  *Retrospective and prospective cohort study* | 1516 severely anemic children divided into 1185 children who had malaria and 331 children with other diagnoses in Kenya | - 3.1 mmol/L | - 2.5 mmol/L  - 3.1 mmol/L with respiratory distress | - Mortality  - Morbidity  - Admission to hospital |

A2.3: Adults

Three adult studies were included from the additional literature review (8, 20, 29).

The characteristics of the included studies are stated in the evidence table below. The studies differ from each other in patient population and in methodology.

**Table 2.** Characteristics of the included studies regarding adults.

| **Included studies** | | | | |
| --- | --- | --- | --- | --- |
| **Study** | **Population** | **Liberal threshold** | **Restrictive threshold** | **Outcomes** |
| *Carson (2002)*  *Retrospective study* | 300 adults with a postoperative Hb of 5.0 with cardiovasculair comorbidities | Different Hb levels | | - Mortality |
| *Hajjar (2010)*  *RCT* | RCT including 512 adults undergoing elective cardiac surgery | - Hematocrit 0.30 L/L | - Hematocrit 0.24 L/L | - Mortality  - Morbidity  - Admission to hospital |
| *Carson (2011)*  *RCT* | 2016 adults with either a history of or risk factors for cardiovascular disease and whose Hb level was of 6.2 mmol/L after hip-fracture surgery | - 6.2 mmol/L | - 5.0 mmol/L | - Mortality  - Morbidity  - Admission to hospital  - Quality of life |

A2.4: Excluded studies

| **Excluded studies** | |
| --- | --- |
| **Study** | **Reasons for exclusion** |
| *Lacroix (2007)* | This study included children in general. For this section the subanalysis of Lacroix (2012) was included. |
| *Lackritz (1992)* | This study included children in general and was included in the section “Children with cancer”. |
| *Viele & Weiskopf (1994)* | This study included adults in general and was included in the section “Children with cancer”. |
| *Carson (2002)* | This study included adults in general and was included in the section “Children with cancer”. |
| *Shander (2014)* | This study included adults in general and was included in the section “Children with cancer”. |
| *Carson (2012)* | This study included adults in general and was included in the section “Children with cancer”. |
| *BCSH (2013)* | This is a former guideline from the BCSH group and was thus not included. |
| *Hébert (1999)* | This study included adults in general and was included in the section “Children with cancer”. |

**B. Results**

B1.1: Hb of 4.3 mmol/L versus Hb greater than 4.3 mmol/L

| Conclusion of evidence | Effect | Level of evidence / studies |
| --- | --- | --- |
| Children with cancer – Systematic literature search | | |
| No studies | - | - |
| Children non-cancer – Evidence cited in existing guidelines | | |
| Mortality | | |
| No significant differences regarding mortality when comparing an Hb of 4.3 mmol/L with 5.0 mmol/L. | RR 0.98 (95% CI 0.14 - 6.77)* | *Very low*** / Willems 2010 |
| Morbidity | | |
| No significant differences regarding morbidity when comparing an Hb of 4.3 mmol/L with 5.0 mmol/L. | RR 0.99 (95% CI 0.64 - 1.54)  RR 1.97 (95% CI 0.62 - 6.20) | *Very low*** / Lacroix 2012 (respiratory distress)  *Very low*** / Willems 2010 |
| Admission to hospital | | |
| No significant differences regarding admission to hospital when comparing an Hb of 4.3 mmol/L with 5.0 mmol/L. | MD 0.10 (95% CI -0.78 - 0.98)  MD -0.40 (95% CI -2.42 - 1.62)* | *Very low*** / Lacroix 2012  *Very low*** / Willems 2010 |
| Adults – Evidence cited in existing guidelines | | |
| Mortality | | |
| No significant differences regarding mortality when comparing an Hb of 4.3 mmol/L with 5.0 mmol/L. | None died (not estimable) | *Very low*** / Carson 2002 |

* Results could not be pooled due to different study populations or outcome measures.

** The level of evidence is taken directly from the relevant guidelines *minus* 1 level for indirectness in this guideline.

B1.2: Hb of 5.0 mmol/L versus Hb greater than 5.0 mmol/L

| Conclusion of evidence | Effect | Level of evidence / studies |
| --- | --- | --- |
| Children with cancer – Systematic literature search | | |
| No studies | - | - |
| Children non-cancer – Evidence cited in existing guidelines | | |
| No studies | - | - |
| Adults – Evidence cited in existing guidelines | | |
| Mortality | | |
| No significant differences regarding mortality when comparing an Hb of 5.0 mmol/L with 6.2 mmol/L. | RR 1.16 (95% CI 0.56 - 2.39)  RR 0.87 (95% CI 0.63 - 1.19)* | *Moderate*** / Hajjar 2010  *Very low*** / Carson 2011 |
| Morbidity | | |
| No significant differences regarding morbidity when comparing an Hb of 5.0 mmol/L with 6.2 mmol/L. | Not significant (no effect measure reported) | *Moderate*** / Hajjar 2010  *Very low*** / Carson 2011 |
| Quality of life | | |
| No significant differences regarding quality of life when comparing an Hb of 5.0 mmol/L with 6.2 mmol/L. | Not significant (no effect measure reported) | *Very low*** / Carson 2011 |
| Admission to hospital | | |
| No significant differences regarding admission to hospital when comparing an Hb of 5.0 mmol/L with 6.2 mmol/L. | Not significant (no effect measure reported)  MD 0.30 (95% CI -0.11 - 0.71) | *Moderate*** / Hajjar 2011  *Very low*** / Carson 2011 |

* Results could not be pooled due to different study populations or oucome measures.

** The level of evidence is taken directly from the relevant guidelines *minus* 1 level for indirectness in this guideline.

B1.3: Hb of 5.6 mmol/L versus Hb greater than 5.6 mmol/L

| Conclusion of evidence | Effect | Level of evidence / studies |
| --- | --- | --- |
| Children with cancer – Systematic literature search | | |
| No studies | - | - |
| Children non-cancer – Evidence cited in existing guidelines | | |
| Mortality | | |
| No significant differences regarding mortality when comparing an Hb of 5.6 mmol/L with 8.07 mmol/L. | RR 0.33 (95% CI 0.01 - 7.87) | *Very low*** / Cholette 2011 |
| Admission to hospital | | |
| No significant differences regarding admission to hospital when comparing an Hb of 5.6 mmol/L with 8.07 mmol/L. | Not significant (no effect measure reported) | *Very low*** / Cholette 2011 |
| Adults – Evidence cited in existing guidelines | | |
| No studies | - | - |

** The level of evidence is taken directly from the relevant guidelines *minus* 1 level for indirectness in this guideline.

**C. Conclusions**

| **VERY LOW QUALITY OF EVIDENCE (GRADE)** | No significant difference regarding mortality, morbidity, and admission to hospital in both children and adults with cardiac and pulmonary comorbidities with *a hemoglobin (Hb) of 4.3 mmol/L versus a hemoglobin (Hb) greater than 4.3 mmol/L* in 3 studies.  Sources (*Lacroix, 2012; Carson, 2002; Willems, 2010*) |
| --- | --- |

**4.2 PROPHYLACTIC RED BLOOD CELL TRANSFUSION IN NEONATES WITH CANCER WITH CARDIAC AND/OR PULMONARY COMORBIDITIES**

**A. Discussion of the literature**

*A1: Discussion of the evidence*

A1.1: Evidence in pediatric oncology

No pediatric oncology studies included neonates.

A1.2: Recommendations and evidence derived from guidelines

Four out of seven guidelines included recommendations for neonates with cardiac and/or pulmonary comorbidities. The Dutch Association of Medical Specialists (21) have recommended a hemoglobin (Hb) threshold of 7.5 mmol/L in neonates less than 1 week old, between weeks 2-3 an Hb threshold of 6.5 and to maintain an Hb threshold of 5.5 mmol/L in neonates older than 3 weeks (49-53). JPAC (2013) (41) have based their recommendations on the BCSH (2016) (44). BCSH (2016) (44) have distinguished their recommendations between neonates on ventilation and neonates on oxygen or CPAP. In case of neonates on ventilation, their advice is to maintain an Hb greater than 7.5 mmol/L in neonates younger than 1 week old. In case of neonates older than 2 weeks old maintain the Hb greater than 6.2 mmol/L. In case of neonates on oxygen or CPAP, their advice is to maintain the Hb greater than 7.5 mmol/L in neonates less then 24 hours old. In case of neonates less than 1 week maintain an Hb greater than 6.2 mmol/L. Between 2 and 3 weeks old maintain the Hb greater than 5.9 mmol/L. In case of neonates older than 4 weeks old, maintain an Hb greater than 5.3 mmol/L (50-54). Valentine (2018) (33) have advised in hemodynamically stable infants with uncorrected congenital heart disease an Hb threshold between 4.3 mmol/L and 5.6 mmol/L, depending on the degree of cardiopulmonary reserve. This recommendation is based on the TRIPICU study stating that an Hb greater than 4.3 mmol/dL is safe (15). However, there is no evidence that transfusion to the Hb greater than 5.6 mmol/dL is beneficial and might be of some risk.

*A2: Description of the included studies*

A2.1: Pediatric oncology

There were no primary pediatric oncology studies included from the systematic literature search.

A2.2: Neonates in general

There were four studies included from the additional literature review (49-53).

The characteristics of the included studies are stated in the evidence table below. The studies differ from each other in patient population (gestational age and birth weight) and in methodology.

**Table 1.** Characteristics of the included studies regarding neonates with cancer with cardiac and/or pulmonary comorbidities.

| **Included studies** | | | | |
| --- | --- | --- | --- | --- |
| Study | Population | Liberal threshold | Restrictive threshold | Outcomes |
| Bell (2005) *RCT* | 103 neonates with very low birth weight (500 to 1300 grams) | - Neonates on ventilation 8.4 mmol/L  - Neonates on oxygen/CPAP 6.9 mmol/L  - Neonates without respiratory support 5.4 mmol/L | - Neonates on ventilation 6.2 mmol/L  - Neonates on oxygen/CPAP 5.0 mmol/L  - Neonates without respiratory support 4.4 mmol/L | - Mortality  - Morbidity  - Costs |
| Chen (2009)  *RCT* | 36 neonates with very low birth weight (<1500 grams) | - Neonates on ventilation 9.1 mmol/L  - Neonates on CPAP 8.3 mmol/L  - Neonates without respiratory support 6.2 mmol/L | - Neonates on ventilation 7.2 mmol/L  - Neonates on CPAP 6.2 mmol/L  - Neonates without respiratory support 4.5 mmol/L | - Mortality  - Morbidity  - Costs |
| Connelly (1999)  *Prospective study* | 24 neonates with very low birth weight (<1500 grams) | - First week of life 8.1 mmol/L  - Second week of life with respiratory support 6.8 mmol/L  - Third week of life 5.0 mmol/L | - First week of life 6.8 mmol/L  - Second week of life with respiratory support 5.6 mmol/L  - Third week of life 5.0 mmol/L | - Mortality  - Morbidity  - Costs |
| Whyte & Kirpalani (2006)  *RCT* | 451 neonates with very low birth weight (<1000 gram) | - Neonates without respiratory support in week 1 7.5 mmol/L  - Neonates without respiratory support in week 2 6.2 mmol/L  - Neonates without respiratory support in week 3 5.3 mmol/L  - Neonates on respiratory support in week 1 8.4 mmol/L  - Neonates on respiratory support in week 2 7.5 mmol/L  - Neonates on respiratory support in week 3 6.2 mmol/L | - Neonates without respiratory support in week 1 6.2 mmol/L  - Neonates without respiratory support in week 2 5.3 mmol/L  - Neonates without respiratory support in week 3 4.7 mmol/L  - Neonates on respiratory support in week 1 7.1 mmol/L  - Neonates on respiratory support in week 2 6.2 mmol/L  - Neonates on respiratory support in week 3 5.3 mmol/L | - Mortality  - Morbidity  - Costs |

A2.3: Excluded studies

| **Excluded studies** | |
| --- | --- |
| **Study** | **Reasons for exclusion** |
| *Whyte & Kirpalani (2011)* | This was a review, including the following studies: Connelly (1999), Bell (2005), Chen (2009), and Whyte & Kirpalani (2006) and were all included. |
| *Venkatesh (2012)* | This was a review, including the following studies: Bell (2005), Chen (2009), Whyte & Kirpalani (2006), Brooks (1999), Mukhopadhya (2004), and Ransome (1989) and were all included except Ransome (1989) considering that this article was not found. |
| *Lacroix (2007)* | This study included children in general and was thus included in the section “Children with cancer”. |

**B. Results**

*B1: Neonates on oxygen/CPAP*

B1.1: Hb of 5.0 mmol/L versus Hb greater than 5.0 mmol/L in neonates on oxygen/CPAP

| Conclusion of evidence | Effect | Level of evidence / studies |
| --- | --- | --- |
| Children with cancer – Systematic literature search | | |
| No studies | - | - |
| Children non-cancer – Evidence cited in existing guidelines | | |
| Mortality | | |
| There was no significant difference regarding mortality when comparing an Hb of 5.6 mmol/L vs. greater than 5.6 mmol/L in neonates. | RR 0.52 (95% CI 0.05 - 5.56). | *Low** / Bell 2005 |
| Morbidity | | |
| There was no significant difference regarding morbidity when comparing an Hb of 5.6 mmol/L vs. greater than 5.6 mmol/L in neonates. | Not significant (no effect measure reported) | *Low** / Bell 2005 |
| Costs | | |
| There was a significant reduction regarding costs when comparing an Hb of 5.6 mmol/L vs. greater than 5.6 mmol/L in neonates. | **MD -1.10 (95% CI -2.10 - -0.10)** | *Low** / Bell 2005 |
| Adults – Evidence cited in existing guidelines | | |
| No studies | - | - |

* The level of evidence is taken directly from the relevant guidelines *minus* 1 level for indirectness in this guideline.

B1.2: Hb of 6.2 mmol/L versus Hb greater than 6.2 mmol/L in neonates on oxygen/CPAP

| Conclusion of evidence | Effect | Level of evidence / studies |
| --- | --- | --- |
| Children with cancer – Systematic literature search | | |
| No studies | - | - |
| Children non-cancer – Evidence cited in existing guidelines | | |
| Mortality | | |
| There was no significant difference regarding mortality when comparing an Hb of 6.2 mmol/L vs. greater than 6.2 mmol/L in neonates. | RR 1.79 (95% CI 0.18 - 18.02) | *Low** / Chen 2009 |
| Morbidity | | |
| There was no significant difference regarding morbidity when comparing an Hb of 6.2 mmol/L vs. greater than 6.2 mmol/L in neonates. | Not significant (no effect measure reported) | *Low** / Chen 2009 |
| Costs | | |
| There was no significant reduction regarding costs when comparing an Hb of 6.2 mmol/L vs. greater than 6.2 mmol/L in neonates. | MD -1.00 (95% CI -2.49 - 0.49) | *Low** / Chen 2009 |
| Adults – Evidence cited in existing guidelines | | |
| No studies | - | - |

* The level of evidence is taken directly from the relevant guidelines *minus* 1 level for indirectness in this guideline.

*B2: Neonates on ventilation*

B2.1: Hb of 6.2 mmol/L versus Hb greater than 6.2 mmol/L in neonates on ventilation

| Conclusion of evidence | Effect | Level of evidence / studies |
| --- | --- | --- |
| Children with cancer – Systematic literature search | | |
| No studies | - | - |
| Children non-cancer – Evidence cited in existing guidelines | | |
| Mortality | | |
| There was no significant difference regarding mortality when comparing an Hb of 6.2 mmol/L vs. greater than 6.2 mmol/L in neonates. | RR 0.52 (95% CI 0.05 - 5.56) | *Low** / Bell 2005 |
| Morbidity | | |
| There was no significant difference regarding morbidity when comparing an Hb of 6.2 mmol/L vs. greater than 6.2 mmol/L in neonates. | Not significant (no effect measure reported) | *Low** / Bell 2005 |
| Costs | | |
| There was a significant reduction regarding costs when comparing an Hb of 6.2 mmol/L vs. greater than 6.2 mmol/L in neonates. | **MD -1.10 (95% CI -2.10 - -0.10)** | *Low** / Bell 2005 |
| Adults – Evidence cited in existing guidelines | | |
| No studies | - | - |

* The level of evidence is taken directly from the relevant guidelines *minus* 1 level for indirectness in this guideline.

B2.2: Hb of 7.5 mmol/L versus Hb greater than 7.5 mmol/L in neonates on ventilation

| Conclusion of evidence | Effect | Level of evidence / studies |
| --- | --- | --- |
| Children with cancer – Systematic literature search | | |
| No studies | - | - |
| Children non-cancer – Evidence cited in existing guidelines | | |
| Mortality | | |
| There was no significant difference regarding mortality when comparing an Hb of 7.5 mmol/L vs. greater than 7.5 mmol/L in neonates. | RR 1.79 (95% CI 0.18 - 18.02) | *Low** / Chen 2009 |
| Morbidity | | |
| There was no significant difference regarding morbidity when comparing an Hb of 7.5 mmol/L vs. greater than 7.5 mmol/L in neonates. | Not significant (no effect measure reported) | *Low** / Chen 2009 |
| Costs | | |
| There was no significant reduction regarding costs when comparing an Hb of 7.5 mmol/L vs. greater than 7.5 mmol/L in neonates. | MD -1.00 (95% CI -2.49 - 0.49) | *Low** / Chen 2009 |
| Adults – Evidence cited in existing guidelines | | |
| No studies | - | - |

* The level of evidence is taken directly from the relevant guidelines *minus* 1 level for indirectness in this guideline.

*B3: Neonates in the first week of life*

B3.1: Hb of 7.5 mmol/L versus Hb greater than 7.5 mmol/L in neonates in the first week of life

| Conclusion of evidence | Effect | Level of evidence / studies |
| --- | --- | --- |
| Children with cancer – Systematic literature search | | |
| No studies | - | - |
| Children non-cancer – Evidence cited in existing guidelines | | |
| Mortality | | |
| There was no significant difference regarding mortality when comparing an Hb of 7.5 mmol/L vs. greater than 7.5 mmol/L in neonates in the first week of life. | RR 1.23 (95% CI 0.84 - 1.79) | *Low** / Whyte & Kirpalani 2006 |
| Morbidity | | |
| There was no significant difference regarding morbidity when comparing an Hb of 7.5 mmol/L vs. greater than 7.5 mmol/L in neonates in the first week of life.  There was a significant difference regarding retinopathy of prematurity when comparing an Hb of 7.5 mmol/L vs. greater than 7.5 mmol/L in neonates in the first week of life. | Not significant (no effect measure reported)  **RR 0.79 (95% CI 0.66 - 0.95)** | *Low** / Whyte & Kirpalani 2006 |
| Costs | | |
| There was no significant difference regarding costs when comparing an Hb of 7.5 mmol/L vs. greater than 7.5 mmol/L in neonates in the first week of life. | MD -0.80 (95% CI -1.65 - 0.05) | *Low** / Whyte & Kirpalani 2006 |
| Adults – Evidence cited in existing guidelines | | |
| No studies | - | - |

* The level of evidence is taken directly from the relevant guidelines *minus* 1 level for indirectness in this guideline.

*B4: Neonates in the second week of life*

B4.1: Hb of 5.6 mmol/L versus Hb greater than 5.6 mmol/L in neonates on respiratory support in the second week of life

| Conclusion of evidence | Effect | Level of evidence / studies |
| --- | --- | --- |
| Children with cancer – Systematic literature search | | |
| No studies | - | - |
| Children non-cancer – Evidence cited in existing guidelines | | |
| Mortality | | |
| There was no significant difference regarding mortality when comparing an Hb of 5.6 mmol/L vs. greater than 5.6 mmol/L in neonates in the second week of life. | None died (not estimable) | *Low** / Connelly 1999 |
| Morbidity | | |
| There was no significant difference regarding morbidity when comparing an Hb of 5.6 mmol/L vs. greater than 5.6 mmol/L in neonates in the second week of life. | Not significant (no effect measure reported) | *Low** / Connelly 1999 |
| Costs | | |
| There was a significant reduction regarding costs when comparing an Hb of 5.6 mmol/L vs. greater than 5.6 mmol/L in neonates in the second week of life. | **MD -2.90 (95% CI -4.94 - -0.86)** | *Low** / Connelly 1999 |
| Adults – Evidence cited in existing guidelines | | |
| No studies | - | - |

* The level of evidence is taken directly from the relevant guidelines *minus* 1 level for indirectness in this guideline.

B4.2: Hb of 6.2 mmol/L versus Hb greater than 6.2 mmol/L in neonates in the second week of life

| Conclusion of evidence | Effect | Level of evidence / studies |
| --- | --- | --- |
| Children with cancer – Systematic literature search | | |
| No studies | - | - |
| Children non-cancer – Evidence cited in existing guidelines | | |
| Mortality | | |
| There was no significant difference regarding mortality when comparing an Hb of 6.2 mmol/L vs. greater than 6.2 mmol/L in neonates in the second week of life. | RR 1.23 (95% CI 0.84 - 1.79) | *Low** / Whyte & Kirpalani 2006 |
| Morbidity | | |
| There was no significant difference regarding morbidity when comparing an Hb of 6.2 mmol/L vs. greater than 6.2 mmol/L in neonates in the second week of life.  There was a significant difference regarding retinopathy of prematurity when comparing an Hb of 6.2 mmol/L vs. greater than 6.2 mmol/L in neonates in the second week of life. | Not significant (no effect measure reported)  **RR 0.79 (95% CI 0.66 - 0.95)** | *Low** / Whyte & Kirpalani 2006 |
| Costs | | |
| There was no significant difference regarding costs when comparing an Hb of 6.2 mmol/L vs. greater than 6.2 mmol/L in neonates in the second week of life. | MD -0.80 (95% CI -1.65 - 0.05) | *Low** / Whyte & Kirpalani 2006 |
| Adults – Evidence cited in existing guidelines | | |
| No studies | - | - |

* The level of evidence is taken directly from the relevant guidelines *minus* 1 level for indirectness in this guideline.

*B5: Neonates in the third week of life*

B5.1: Hb of 5.6 mmol/L versus Hb greater than 5.6 mmol/L in neonates on respiratory support in the third week of life

| Conclusion of evidence | Effect | Level of evidence / studies |
| --- | --- | --- |
| Children with cancer – Systematic literature search | | |
| No studies | - | - |
| Children non-cancer – Evidence cited in existing guidelines | | |
| Mortality | | |
| There was no significant difference regarding mortality when comparing an Hb of 5.6 mmol/L vs. greater than 5.6 mmol/L in neonates in the third week of life. | RR 1.23 (95% CI 0.84 - 1.79) | *Low** / Whyte & Kirpalani 2006 |
| Morbidity | | |
| There was no significant difference regarding morbidity when comparing an Hb of 5.6 mmol/L vs. greater than 5.6 mmol/L in neonates in the third week of life.  There was a significant difference regarding retinopathy of prematurity when comparing an Hb of 5.6 mmol/L vs. greater than 5.6 mmol/L in neonates in the third week of life. | Not significant (no effect measure reported)  **RR 0.79 (95% CI 0.66 - 0.95)** | *Low** / Whyte & Kirpalani 2006 |
| Costs | | |
| There was no significant difference regarding costs when comparing an Hb of 5.6 mmol/L vs. greater than 5.6 mmol/L in neonates in the third week of life. | MD -0.80 (95% CI - 1.65 - 0.05) | *Low** / Whyte & Kirpalani 2006 |
| Adults – Evidence cited in existing guidelines | | |
| No studies | - | - |

* The level of evidence is taken directly from the relevant guidelines *minus* 1 level for indirectness in this guideline.

**C. Conclusions**

No conclusions were formulated.

**5. PROPHYLACTIC RED BLOOD CELL TRANSFUSION - HYPERLEUKOCYTOSIS**

**5.1 PROPHYLACTIC RED BLOOD CELL TRANSFUSION IN CHILDREN AND NEONATES WITH CANCER DURING HYPERLEUKOCYTOSIS**

**A. Discussion of the literature**

*A1: Discussion of the evidence*

A1.1: Evidence in pediatric oncology

No pediatric oncology studies included children or neonates with cancer during hyperleukocytosis.

A1.2: Recommendations and evidence derived from guidelines

No guidelines included children or neonates with cancer during hyperleukocytosis.

*A2: Description of the included studies*

There were no primary pediatric oncology studies included from the systematic literature search and no additional studies from the additional literature review.

**B. Results**

As no studies were included, no results were presented.

**C. Conclusions**

As no studies were included, no conclusions were formulated.

**6. IRRADIATED RED BLOOD CELL TRANSFUSIONS
6.1 IRRADIATED RED BLOOD CELL TRANSFUSIONS IN CHILDREN AND NEONATES WITH CANCER**

**A. Discussion of the literature**

*A1: Discussion of the evidence*

A1.1: Evidence in pediatric oncology

No pediatric oncology studies were included.

A1.2: Recommendations and evidence derived from guidelines

The only guideline that included recommendations for irradiated RBC products was the Dutch Association of Medical Specialists (21) based on one study (34) and a survey under hemovigilance organizations worldwide:

- In case of HLA related products and donors:

1. Transfusion between 1st to 3rd degree relatives of cell-containing blood products;
2. HLA-compatible plated concentrates.

- In case of granulocyte transfusions
- Depending on the patient's immune status:

1. During intrauterine transfusions until 6 months after the due date;
2. Children with congenital combined immune deficiencies (e.g., SCID);
3. Acquired immune deficiencies such as:
   - Allogeneic stem cell transplantations up to 1 year after transplantation;
   - Autologous stem cell transplantations up to 6 months after transplantation;
   - After application of donor lymphocyte infusion (DLI) or infusion of cytotoxic T lymphocytes (CTL) up to 1 year after transfusion.

- In case of patients with prolonged T-cell depletion after medication:

1. Fludarabine or other T-cell depleting therapy or indicated by the pharmacotherapeutic compass (up to 6 months after discontinuation of the therapy);
2. Medications that, in combination with the disease, cause long-term T-cell depletions, such as anti-CD52 treatments in hematological diseases and ATG treatment in aplastic anemia from the initiation to 6 months after completion of the treatment.

*A2: Description of the included studies*

There were no primary pediatric oncology studies included from the systematic literature search and no additional studies from the additional literature review.

**B. Results**

As no studies were included, no results were presented.

**C. Conclusions**

As no studies were included, no conclusions were formulated.

**7. LOW OR HIGH-VOLUME RED BLOOD CELL TRANSFUSIONS**

**7.1 LOW OR HIGH-VOLUME RED BLOOD CELL TRANSFUSIONS IN CHILDREN WITH CANCER**
**A. Discussion of the literature***A1: Discussion of the evidence*A1.1: Evidence in pediatric oncology
No pediatric oncology studies included RBC volumes in children.

A1.2: Recommendations and evidence derived from guidelines

No guidelines included RBC volumes in children.

*A2: Description of the included studies*

A2.1: Pediatric oncology

There were no primary pediatric oncology studies included from the systematic literature search.

A2.2: Children in general

One pediatric study was included from the additional literature review (38).

The characteristics of the included study are stated in the evidence table below.

**Table 1.** Characteristics of the included studies regarding children.

| **Included studies** | | | | |
| --- | --- | --- | --- | --- |
| **Study** | **Population** | **Liberal volume** | **Restrictive volume** | **Outcomes** |
| *Olupot-Olupot (2014)*  *RCT* | 160 children with severe anemia (Hb of 3.7 mmol/L) | - 30 ml/kg | - 20 ml/kg | - Mortality  - Morbidity  - Costs |

**B. Results**

B1.1 Prophylactic RBC transfusion volume 20 ml/kg versus >20 ml/kg

| Conclusion of evidence | Effect | Level of evidence / studies |
| --- | --- | --- |
| Children with cancer – Systematic literature search | | |
| No studies | - | - |
| Children non-cancer – Evidence cited in existing guidelines | | |
| Mortality | | |
| No significant differences in group with volume of 20 ml/kg vs. higher than 20 ml/kg | RR 5.71 (95% CI 0.70 - 46.34) | *Very low** / Olupot-Olupot 2014 |
| Morbidity | | |
| No significant differences in group with volume of 20 ml/kg vs. higher than 20 ml/kg | RR 2.85 (95% CI 0.59 - 13.72) | *Very low** / Olupot-Olupot 2014 |
| Costs | | |
| No significant differences in group with volume of 20 ml/kg vs. higher than 20 ml/kg | RR 2.85 (95% CI 0.96 - 8.47) | *Very low** / Olupot-Olupot 2014 |
| Adults with cancer – Evidence cited in existing guidelines | | |
| No studies | - | - |

* The level of evidence is taken directly from the concerned guidelines *minus* 1 level for indirectness in this guideline.

**C. Conclusions**

| **VERY LOW QUALITY OF EVIDENCE (GRADE)** | No significant difference regarding mortality, morbidity and costs with a *volume of 20 ml/kg versus higher than 20 ml/kg* in 1 study.  Sources (*Olupot-Olupot, 2014*) |
| --- | --- |

**7.2 LOW OR HIGH-VOLUME RED BLOOD CELL TRANSFUSIONS IN NEONATES WITH CANCER
A. Discussion of the literature***A1: Discussion of the evidence*A1.1: Evidence in pediatric oncology
No pediatric oncology studies included low or high-volume prophylactic RBC transfusions

A1.2: Recommendations and evidence derived from guidelines

Two out of seven guidelines included recommendations regarding the volume of the prophylactic RBC transfusion. All (21, 42) recommend a volume of 15 ml/kg in neonates. These recommendations were based on 4 studies (35-38).

*A2: Description of the included studies*

A2.1: Pediatric oncology

There were no primary pediatric oncology studies included from the systematic literature search.

A2.2: Children in general

Three pediatric studies were included from the additional literature review (35-37).

The characteristics of the included studies are stated in the evidence table below. The studies differ from each other in patient population and in methodology.

**Table 1.** Characteristics of the included studies regarding children.

| **Included studies** | | | | |
| --- | --- | --- | --- | --- |
| **Study** | **Population** | **Liberal volume** | **Restrictive volume** | **Outcomes** |
| *Paul (2002)*  *RCT* | 13 neonates with a very low birthweight (<1500 grams) | - 20 ml/kg | - 10 ml/kg | - Mortality |
| *Wong (2005)*  *RCT* | 20 neonates with a very low birthweight (<1500 grams) | - 20 ml/kg | - 15 ml/kg | - Mortality |
| *Khodabux (2009)*  *Observational study* | 459 premature born neonates with a gestational age between 24+0 and 31+6 weeks | - 20 ml/kg | - 15 ml/kg | - Mortality  - Morbidity |

A2.3: Excluded studies

| **Excluded studies** | |
| --- | --- |
| **Study** | **Reasons for exclusion** |
| *Olupot-Olupot (2014)* | This study included children and was thus included in the section “Low or high-volume transfusion in children”. |

**B. Results**

B1.1: Prophylactic RBC transfusion volume 10 ml/kg versus higher than 10 ml/kg

| Conclusion of evidence | Effect | Level of evidence / studies |
| --- | --- | --- |
| Children with cancer – Systematic literature search | | |
| No studies | - | - |
| Children non-cancer – Evidence cited in existing guidelines | | |
| Morbidity | | |
| No significant differences in group with volume of 10 ml/kg vs. higher than 10 ml/kg | Not significant (no effect measure reported) | *Very low*** / Paul 2002 |
| Adults with cancer – Evidence cited in existing guidelines | | |
| No studies | - | - |

** The level of evidence is taken directly from the relevant guidelines *minus* 1 level for indirectness in this guideline.

B1.2: Prophylactic RBC transfusion volume 15 ml/kg versus >15 ml/kg

| Conclusion of evidence | Effect | Level of evidence / studies |
| --- | --- | --- |
| Children with cancer – Systematic literature search | | |
| No studies | - | - |
| Children non-cancer – Evidence cited in existing guidelines | | |
| Mortality | | |
| No significant differences in group with volume of 15 ml/kg vs. higher than 15 ml/kg | RR 1.00 (95% CI 0.07 - 13.87)  RR 0.94 (95% CI 0.43 - 2.04)* | *Very low*** / Wong 2005  *Very low*** / Khodabux 2009 |
| Morbidity | | |
| No significant differences in group with volume of 15 ml/kg vs. higher than 15 ml/kg | Not significant (no effect measure reported) | *Very low*** / Wong 2005  *Very low*** / Khodabux 2009 |
| Adults with cancer – Evidence cited in existing guidelines | | |
| No studies | - | - |

* Results could not be pooled due to different study populations or outcome measures.

** The level of evidence is taken directly from the relevant guidelines *minus* 1 level for indirectness in this guideline.

**C. Conclusions**

| **VERY LOW QUALITY OF EVIDENCE (GRADE)** | No significant difference regarding morbidity with a *volume of 10 ml/kg versus higher than 10 ml/kg* in 1 study.  Sources (*Paul, 2002*) |
| --- | --- |

| **VERY LOW QUALITY OF EVIDENCE (GRADE)** | No significant difference regarding mortality and morbidity with a *volume of 15 ml/kg versus higher than 15 ml/kg* in 2 studies.  Sources (*Khodabux, 2009; Wong, 2005*) |
| --- | --- |

**8. INFUSION RATES OF RED BLOOD TRANSFUSIONS**

**8.1 INFUSION RATES OF RED BLOOD TRANSFUSIONS IN CHILDREN WITH CANCER
A. Discussion of the literature***A1: Discussion of the evidence*A1.1: Evidence in pediatric oncology
No pediatric oncology studies included children.

A1.2: Recommendations and evidence derived from guidelines

One guideline was identified. JPAC (2013) (41) has recommended a RBC infusion rate of 5 ml/kg/hour and the transfusion must be completed within 4 hours, this was based on consensus.

*A2: Description of the included studies*

There were no primary pediatric oncology studies included from the systematic literature search and no additional studies from the additional literature review.

**B. Results**

As no studies were included, no results were presented.

**C. Conclusions**

As no studies were included, no conclusions were formulated.

**8.2 INFUSION RATES OF RED BLOOD TRANSFUSIONS IN NEONATES WITH CANCER
A. Discussion of the literature**

*A1: Discussion of the evidence*

A1.1: Evidence in pediatric oncology

No pediatric oncology studies included neonates.

A1.2: Recommendations and evidence derived from guidelines

The Dutch Association of Medical Specialists (21) has recommended an infusion rate in neonates of 5 ml/kg/hour based on consensus.

*A2: Description of the included studies*

There were no primary pediatric oncology studies included from the systematic literature search and no additional studies from the additional literature review.

**B. Results**

As no studies were included, no results were presented.

**C. Conclusions**

As no studies were included, no conclusions were formulated.

REFERENCES

1. Lightdale JR, Randolph AG, Tran CM, Jiang H, Colon A, Houlahan K, Lehmann LE (2012) Impact of a conservative red blood cell transfusion strategy in children undergoing hematopoietic stem cell transplantation. Biol Blood Marrow Transplant 18(5):813–817. https://doi.org/10.1016/j.bbmt.2011.10.043

2. Bateman ST, Lacroix J, Boven K, Forbes P, Barton R, Thomas NJ, Jacobs B, Markovitz B, Goldstein B, Hanson JH, Li HA, Randolph AG (2008) Anemia, blood loss, and blood transfusions in North American children in the intensive care unit. Am J Respir Crit Care Med 178(1):26–33. https://doi.org/10.1164/rccm.200711-1637oc

3. Lucarelli G, Galimberti M, Polchi P, Angelucci E, Baronciani D, Giardini C, Politi P, Durazzi SMT, Muretto P, Albertini F (1990) Bone marrow transplantation in patients with thalassemia. N Engl J Med 322(7):417–421. https://doi.org/10.1056/nejm199002153220701

4. Cochrane handbook for systematic reviews of interventions (2011) Cochrane handbook for systematic reviews of interventions. https://handbook-5-1.cochrane.org/

5. Mulder RL, Brown MC, Skinner R, van Dalen EC, Hudson MM, Kremer LCM (2021) Handbook for guideline development; collaboration between International Guideline Harmonization Group, PanCare Guideline Group and Cochrane Childhood Cancer

6. Schünemann H, Brożek J, Guyatt G, Oxman A (2013) GRADE handbook. GRADE handbook. https://netherlands.cochrane.org/sites/netherlands.cochrane.org/files/public/uploads/6_agree_ii_dutch.pdf

7. AGREE Next Steps Consortium. Appraisal of Guidelines for Research & Evaluation (AGREE) II Instrument. www.agreetrust.org

8. Guyatt GH, Oxman AD, Vist GE, Kunz R, Falck-Ytter Y, Alonso-Coello P et al (2008) GRADE: an emerging consensus on rating quality of evidence and strength of recommendations. BMJ 336(7650):924–926

9. Kirpalani H, Whyte RK, Andersen C, Asztalos EV, Heddle N, Blajchman MA, Peliowski A, Rios A, LaCorte M, Connelly R, Barrington K, Roberts RS (2006) The premature infants in need of transfusion (pint) study: a randomized, controlled trial of a restrictive (LOW) versus liberal (HIGH) transfusion threshold for extremely low birth weight infants. J Pediatr 149(3):301-307.e3. https://doi.org/10.1016/j.jpeds.2006.05.011

10. Robitaille N, Lacroix J, Alexandrov L, Clayton L, Cortier M, Schultz KR, Duval M (2013) Excess of veno-occlusive disease in a randomized clinical trial on a higher trigger for red blood cell transfusion after bone marrow transplantation: a Canadian blood and marrow transplant group trial. Biol Blood Marrow Transplant 19(3):468–473. https://doi.org/10.1016/j.bbmt.2012.12.002

11. Carson JL, Noveck H, Berlin JA, Gould SA (2002) Mortality and morbidity in patients with very low postoperative Hb levels who decline blood transfusion. Transfusion 42(7):812–818. https://doi.org/10.1046/j.1537-2995.2002.00123.x

12. Carson JL, Carless PA, Hébert PC (2012) Transfusion thresholds and other strategies for guiding allogeneic red blood cell transfusion. Cochrane Database Syst Rev 1–62. https://doi.org/10.1002/14651858.cd002042.pub3

13. Shander A, Javidroozi M, Naqvi S, Aregbeyen O, Çaylan M, Demir S, Juhl A (2014) An update on mortality and morbidity in patients with very low postoperative hemoglobin levels who decline blood transfusion (CME). Transfusion 54(10pt2):2688–2695. https://doi.org/10.1111/trf.12565

14. Lacroix J, Hébert PC, Hutchison JS, Hume HA, Tucci M, Ducruet T, Gauvin F, Collet J-P, Toledano BJ, Robillard P, Joffe A, Biarent D, Meert K, Peters MJ (2007) Transfusion strategies for patients in pediatric intensive care units. N Engl J Med 356(16):1609–1619. https://doi.org/10.1056/nejmoa066240

15. Hébert PC, Wells G, Blajchman MA, Marshall J, Martin C, Pagliarello G, Tweeddale M, Schweitzer I, Yetisir E (1999) A multicenter, randomized, controlled clinical trial of transfusion requirements in critical care. N Engl J Med 340(6):409–417. https://doi.org/10.1056/nejm199902113400601

16. Rohde JM, Dimcheff DE, Blumberg N, Saint S, Langa KM, Kuhn L, Hickner A, Rogers MAM (2014) Health care–associated infection after red blood cell transfusion. JAMA 311(13):1317. https://doi.org/10.1001/jama.2014.2726

17. Lacroix J, Demaret P, Tucci M (2012) Red blood cell transfusion: decision making in pediatric intensive care units. Semin Perinatol 36(4):225–231. https://doi.org/10.1053/j.semperi.2012.04.002

18. Jansen AJG, Essink-Bot M, Beckers EAM, Hop WCJ, Schipperus MR, Van Rhenen D (2003) Quality of life measurement in patients with transfusion-dependent myelodysplastic syndromes. Br J Haematol 121(2):270–274. https://doi.org/10.1046/j.1365-2141.2003.04272.x

19. Carson JL, Terrin ML, Noveck H, Sanders DW, Chaitman BR, Rhoads GG, Nemo G, Dragert K, Beaupre L, Hildebrand K, Macaulay W, Lewis C, Cook DR, Dobbin G, Zakriya KJ, Apple FS, Horney RA, Magaziner J (2011) Liberal or restrictive transfusion in high-risk patients after hip surgery. N Engl J Med 365(26):2453–2462. https://doi.org/10.1056/nejmoa1012452

20. Lackritz EM, Hightower AW, Zucker JR, Ruebush TK, Onudi CO, Steketee RW, Were JBO, Patrick E, Campbell CC (1997) Longitudinal evaluation of severely anemic children in Kenya. AIDS 11(12):1487–1494. https://doi.org/10.1097/00002030-199712000-00013

21. Viele MK, Weiskopf RB (1994) What can we learn about the need for transfusion from patients who refuse blood? The experience with Jehovah’s witnesses. Transfusion 34(5):396–401. https://doi.org/10.1046/j.1537-2995.1994.34594249050.x

22. Federation of Medical Specialists (2019) Startpagina - Bloedtransfusiebeleid - Richtlijn - Richtlijnendatabase. Federation of Medical Specialists. https://richtlijnendatabase.nl/richtlijn/bloedtransfusiebeleid/startpagina_-_bloedtransfusiebeleid.html

23. Holst LB, Haase N, Wetterslev J, Wernerman J, Guttormsen AB, Karlsson S, Johansson PI, Åneman A, Vang ML, Winding R, Nebrich L, Nibro HL, Rasmussen BS, Lauridsen JRM, Nielsen JS, Oldner A, Pettilä V, Cronhjort MB, Andersen LH, Perner A (2014) Lower versus higher hemoglobin threshold for transfusion in septic shock. N Engl J Med 371(15):1381–1391. https://doi.org/10.1056/nejmoa1406617

24. Muszynski JA, Guzzetta NA, Hall MW, Macrae D, Valentine SL, Bateman ST, Spinella PC (2018) Recommendations on RBC transfusions for critically ill children with nonhemorrhagic shock from the pediatric critical care transfusion and anemia expertise initiative. Pediatr Crit Care Med 19:S121–S126. https://doi.org/10.1097/pcc.0000000000001620

25. Henke M, Sindlinger F, Ikenberg H, Gerds T, Schumacher M (2004) Blood hemoglobin level and treatment outcome of early breast cancer. Strahlenther Onkol 180(1):45–51. https://doi.org/10.1007/s00066-004-1123-7

26. Hoff CM, Hansen HS, Overgaard M, Grau C, Johansen J, Bentzen J, Overgaard J (2011) The importance of haemoglobin level and effect of transfusion in HNSCC patients treated with radiotherapy—results from the randomized DAHANCA 5 study. Radiother Oncol 98(1):28–33. https://doi.org/10.1016/j.radonc.2010.09.024

27. Hoff CM, Lassen P, Eriksen JG, Hansen HS, Specht L, Overgaard M, Grau C, Johansen J, Bentzen J, Andersen L, Evensen JF, Overgaard J (2011) Does transfusion improve the outcome for HNSCC patients treated with radiotherapy?—results from the randomized DAHANCA 5 and 7 trials. Acta Oncol 50(7):1006–1014. https://doi.org/10.3109/0284186x.2011.592650

28. Janssens GO, Rademakers SE, Terhaard CH, Doornaert PA, Bijl HP, van den Ende P, Chin A, Takes RP, de Bree R, Hoogsteen IJ, Bussink J, Span PN, Kaanders JH (2014) Improved recurrence-free survival with ARCON for anemic patients with laryngeal cancer. Clin Cancer Res 20(5):1345–1354. https://doi.org/10.1158/1078-0432.ccr-13-1730

29. Patient Blood Management Guidelines: Module 3 (2012) Patient Blood Management Guidelines National Blood Authority. https://www.blood.gov.au/pubs/pbm/module3/abbreviations_and_acronyms.html

30. Willems A, Harrington K, Lacroix J, Biarent D, Joffe AR, Wensley D, Ducruet T, Hébert PC, Tucci M (2010) Comparison of two red-cell transfusion strategies after pediatric cardiac surgery: a subgroup analysis. Crit Care Med 38(2):649–656. https://doi.org/10.1097/ccm.0b013e3181bc816c

31. Hajjar LA, Vincent J-L, Galas FRBG, Nakamura RE, Silva CMP, Santos MH, Fukushima J, Filho RK, Sierra DB, Lopes NH, Mauad T, Roquim AC, Sundin MR, Leão WC, Almeida JP, Pomerantzeff PM, Dallan LO, Jatene FB, Stolf NAG, Auler JOC (2010) Transfusion requirements after cardiac surgery. JAMA 304(14):1559. https://doi.org/10.1001/jama.2010.1446

32. Cholette JM, Rubenstein JS, Alfieris GM, Powers KS, Eaton M, Lerner NB (2011) Children with single-ventricle physiology do not benefit from higher hemoglobin levels post cavopulmonary connection: results of a prospective, randomized, controlled trial of a restrictive versus liberal red-cell transfusion strategy*. Pediatr Crit Care Med 12(1):39–45. https://doi.org/10.1097/pcc.0b013e3181e329db

33. Valentine SL, Bembea MM, Muszynski JA, Cholette JM, Doctor A, Spinella PC, Steiner ME, Tucci M, Hassan NE, Parker RI, Lacroix J, Argent A, Carson JL, Remy KE, Demaret P, Emeriaud G, Kneyber MCJ, Guzzetta N, Hall MW, Bateman ST (2018) Consensus recommendations for RBC transfusion practice in critically ill children from the pediatric critical care transfusion and anemia expertise initiative. Pediatr Crit Care Med 19(9):884–898. https://www.ncbi.nlm.nih.gov/pmc/articles/PMC6126913/pdf/nihms966887.pdf32.332

34. Giammarco S, Chiusolo P, Piccirillo N, Di Giovanni A, Metafuni E, Laurenti L, Sica S, Pagano L (2016) Hyperleukocytosis and leukostasis: management of a medical emergency. Expert Rev Hematol 10(2):147–154. https://doi.org/10.1080/17474086.2017.1270754

35. Padmanabhan A, Connelly-Smith L, Aqui N, Balogun RA, Klingel R, Meyer E, Pham HP, Schneiderman J, Witt V, Wu Y, Zantek ND, Dunbar NM, Schwartz GEJ (2019) Guidelines on the use of therapeutic apheresis in clinical practice—evidence-based approach from the Writing Committee of the American Society for Apheresis: the eighth special Issue. J Clin Apheresis 34(3):171–354. https://doi.org/10.1002/jca.21705

36. Kopolovic I, Ostro J, Tsubota H, Lin Y, Cserti-Gazdewich CM, Messner HA, Keir AK, DenHollander N, Dzik WS, Callum J (2015) A systematic review of transfusion-associated graft-versus-host disease. Blood 126(3):406–414. https://doi.org/10.1182/blood-2015-01-620872

37. Paul DA, Leef KH, Locke RG, Stefano JL (2002) Transfusion volume in infants with very low birth weight: a randomized trial of 10 versus 20 mL/kg. J Pediatr Hematol Oncol 24(1):43–46. https://doi.org/10.1097/00043426-200201000-00012

38. Khodabux CM, Hack KEA, von Lindern JS, Brouwers H, Walther FJ, Brand A (2009) A comparative cohort study on transfusion practice and outcome in two Dutch tertiary neonatal centres. Transfus Med 19(4):195–201. https://doi.org/10.1111/j.1365-3148.2009.00934.x

39. Wong H, Connelly R, Day A, Flavin MP (2007) A comparison of high and standard blood transfusion volumes in premature infants. Acta Paediatr 94(5):624–625. https://doi.org/10.1111/j.1651-2227.2005.tb01949.x

40. Olupot-Olupot P, Engoru C, Thompson J, Nteziyaremye J, Chebet M, Ssenyondo T, Dambisya CM, Okuuny V, Wokulira R, Amorut D, Ongodia P, Mpoya A, Williams TN, Uyoga S, Macharia A, Gibb DM, Walker AS, Maitland K (2014) Phase II trial of standard versus increased transfusion volume in Ugandan children with acute severe anemia. BMC Med 12(1):67. https://doi.org/10.1186/1741-7015-12-67

41. JPAC. United Kingdom Blood Services (2013) Handbook of transfusion medicine 5th Edi (5th ed., 2013 editie). TSO

42. *Richtlijn bloedtransfusie* (2011) CBO. https://www.nvog.nl/wp-content/uploads/2018/02/Bloedtransfusie-2.0-11-11-2011.pdf

43. NICE (2015) Everview | blood transfusion | guidance | NICE. https://www.nice.org.uk/guidance/ng24/evidence/full-guideline-pdf-2177160733

44. New HV, Berryman J, Bolton-Maggs PHB, Cantwell C, Chalmers EA, Davies T, Gottstein R, Kelleher A, Kumar S, Morley SL, Stanworth SJ (2016) Guidelines on transfusion for fetuses, neonates and older children. Br J Haematol 175(5):784–828. https://doi.org/10.1111/bjh.14233

45. Smith PJ, Ekert H (1976) Evidence of stem-cell competition in children with malignant disease. The Lancet 307(7963):776–779. https://doi.org/10.1016/s0140-6736(76)91613-5

46. Toogood I (1978) Controlled study of hypertransfusion during remission induction in childhood acute lymphocytic leukemia. The Lancet 312(8095):862–864. https://doi.org/10.1016/s0140-6736(78)91570-2

47. English M, Ahmed M, Ngando C, Berkley J, Ross A (2002) Blood transfusion for severe anaemia in children in a Kenyan hospital. The Lancet 359(9305):494–495. https://doi.org/10.1016/s0140-6736(02)07666-3

48. Lackritz EM, Campbell CC, Ruebush TK, Hightower AW, Wakube W, Were JBO (1992) Effect of blood transfusion on survival among children in a Kenyan hospital. The Lancet 340(8818):524–528. https://doi.org/10.1016/0140-6736(92)91719-o

49. Akech SO, Hassall O, Pamba A, Idro R, Williams TN, Newton CRJC, Maitland K (2008) Survival and haematological recovery of children with severe malaria transfused in accordance to WHO guidelines in Kilifi, Kenya. Malaria J 7(1):256. https://doi.org/10.1186/1475-2875-7-256

50. Connelly RJ, Stone SH, Whyte RK (1999) Early versus late red cell transfusion in low-birth-weight infants. Pediatr Res 43(4):170A

51. Bell EF (2005) Randomized trial of liberal versus restrictive guidelines for red blood cell transfusion in preterm infants. Pediatrics 115(6):1685–1691. https://doi.org/10.1542/peds.2004-1884

52. Whyte R, Kirpalani H (2011) Low versus high haemoglobin concentration threshold for blood transfusion for preventing morbidity and mortality in very low birth weight infants. Cochrane Database Syst Rev 1. https://doi.org/10.1002/14651858.cd000512.pub2

53. Chen H-L, Tseng H-I, Lu C-C, Yang S-N, Fan H-C, Yang R-C (2009) Effect of blood transfusions on the outcome of very low body weight preterm infants under two different transfusion criteria. Pediatr Neonatol 50(3):110–116. https://doi.org/10.1016/s1875-9572(09)60045-0

54. Venkatesh V, Khan R, Curley A, Hopewell S, Doree C, Stanworth S (2012) The safety and efficacy of red cell transfusions in neonates: a systematic review of randomized controlled trials. Br J Haematol 158(3):370–385. https://doi.org/10.1111/j.1365-2141.2012.09180.x

55. Brooks SE, Marcus DM, Gillis D, Pirie E, Johnson MH, C. R. N. I. §., Bhatia J (1999) The effect of blood transfusion protocol on retinopathy of prematurity: a prospective, randomized study. Pediatrics 104(3):514–518. https://doi.org/10.1542/peds.104.3.514

56. Mukhopadhyay K, Ghosh PS, Narang A, Dogra MR (2004) Cut off level for RBC trans-fusion in sick preterm neonates. PediatricResearch 55:288A

57. Marsh K, Forster D, Waruiru C, Mwangi I, Winstanley M, Marsh V, Newton CR, Winstanley P, Warn P, Peshu N, Pasvol G, Snow RW (1995) Indicators of life-threatening malaria in African children. N Engl J Med 332(21):1399–1404. https://doi.org/10.1056/nejm199505253322102

58. Retter A, Wyncoll D, Pearse R, Carson D, McKechnie S, Stanworth S, Allard S, Thomas D, Walsh T (2012) Guidelines on the management of anaemia and red cell transfusion in adult critically ill patients. Br J Haematol 160(4):445–464. https://doi.org/10.1111/bjh.12143

59.Mulder RL, Font-Gonzalez A, Hudson MM, Van Santen HM, Loeffen EAH, Burns K, Quinn GP, Van Dulmen-Den Broeder E, Byrne J, Haupt R, Wallace WH, Van Den Heuvel-Eibrink MM, Anazodo A, Anderson RA, Barnbrock A, Beck JD, Bos AME, Demeestere I, Denzer C, Verhaak CM (2021) Fertility preservation for female patients with childhood, adolescent, and young adult cancer: recommendations from the PanCareLIFE Consortium and the international late effects of childhood cancer guideline harmonization group. Lancet Oncol 22(2):e45–e56. https://doi.org/10.1016/s1470-2045(20)30594-5
