# Supplementary material for: Prophylactic red blood cell transfusions in children and neonates with cancer: An evidence-based clinical practice guideline
Source: Support Care Cancer. 2024 Nov 4;32(11):766. doi: 10.1007/s00520-024-08888-3 (PMC11534970; doi:10.1007/s00520-024-08888-3)
Supplement: Supplementary file 8 — Supplementary file8 (DOCX 19 KB) [file 520_2024_8888_MOESM8_ESM.docx]

**Supplemental Materials 8: Overview of the recommendations**

**Table 1a.** Overview of red blood cell transfusion recommendations for children with cancer

| Recommendation | Strength of recommendation | Quality of evidence |
| --- | --- | --- |
| Children with cancer | | |
| Recommendation 1.1.1. We suggest a hemoglobin (Hb) threshold of 4.3 mmol/L for red blood cell (RBC) transfusion in children with cancer. | WEAK | VERY LOW QUALITY evidence |
| Recommendation 1.1.2. We suggest *against* a hemoglobin (Hb) threshold of 3.7 mmol/L for red blood cell (RBC) transfusion in children with cancer. | WEAK | VERY LOW QUALITY evidence |
| Recommendation 1.1.3. We recommend *against* a hemoglobin (Hb) threshold of 3.1 mmol/L or lower for red blood cell (RBC) transfusion in children with cancer. | STRONG | VERY LOW QUALITY evidence |
| Children with cancer during sepsis | | |
| Recommendation 2.1.1. We suggest a hemoglobin (Hb) threshold of 4.3 mmol/L for red blood cell (RBC) transfusion in children with cancer during sepsis who are hemodynamically stable. | WEAK | VERY LOW QUALITY evidence |
| Recommendation 2.1.2. We believe that for hemodynamically unstable children with cancer during sepsis and evidence of oxygen deficiency (e.g., use of inotropes, elevated lactate), an Hb threshold that ranges between 4.3 mmol/L and 6.2 mmol/L can be considered. | - | EXPERT opinion |
| Children with cancer who undergo radiotherapy | | |
| Recommendation 3.1.1. We believe a hemoglobin (Hb) threshold of 4.3 mmol/L for red blood cell (RBC) transfusion should be maintained in children with cancer who undergo radiotherapy. | - | EXPERT opinion |
| Children with cancer with cardiac and/or pulmonary comorbidities | | |
| Recommendation 4.1.1. We suggest a hemoglobin (Hb) threshold of 4.3 mmol/L for red blood cell (RBC) transfusion in children with cancer and cardiac and pulmonary comorbidities. | WEAK | VERY LOW QUALITY evidence |
| Recommendation 4.1.2. We believe that in case of a hemodynamically unstable child with cancer and pulmonary and/or cardiac comorbidities (e.g., use of inotropes, elevated lactate) a higher Hb threshold can be considered. | - | EXPERT opinion |
| Recommendation 4.1.3. For children on ECMO:  - In critically ill children on ECMO, there is insufficient evidence to recommend a specific RBC transfusion decision-making strategy using physiologic-based metrics and biomarkers.  - In critically ill children on ECMO, we believe in using physiologic metrics and biomarkers of oxygen delivery in addition to Hb concentration to guide RBC transfusion. Administration of a RBC transfusion should be based on evidence of inadequate cardiorespiratory support or decreased systemic and/or regional oxygen delivery. | - | EXPERT opinion |
| Children with cancer during hyperleukocytosis | | |
| Recommendation 5.1.1. In children with cancer and hyperleukocytosis, we believe that a RBC transfusion should be given with restraint until the number of leukocytes has fallen below 100 x 109 /L or in the presence of clinical symptoms of hyperleukocytosis | - | EXPERT opinion |
| Recommendation 5.1.2. In children with cancer and hyperleukocytosis, we believe that a RBC transfusion should be given with restraint, unless there are severe clinical signs of anemia or in case of an Hb below 3.1 mmol/L. | - | EXPERT opinion |
| Recommendation 5.1.3. If needed, transfuse with a maximum of 5 ml/kg/4-6 hours. | - | EXPERT opinion |
| Irradiated red blood cell transfusions in children with cancer | | |
| Recommendation 6.1.1. We believe that irradiated blood products should be used in case of an HLA related product and donor: a) Transfusion between 1st to 3rd degree relatives of cell-containing blood products;  b) HLA-compatible platelet concentrates. | - | EXPERT opinion |
| Recommendation 6.1.2. We believe that irradiated blood products should be used in case of granulocyte transfusions. | - | EXPERT opinion |
| Recommendation 6.1.3. We believe that irradiated blood products should be used depending on the patient's immune status:  a) During intrauterine transfusions until 6 months after the due date;  b) Children with congenital combined immune deficiencies (e.g. SCID);  c) Acquired immune deficiencies such as:  - Allogeneic stem cell transplantations up to 1 year after transplantation;  - Autologous stem cell transplantations up to 6 months after transplantation;  - After application of donor lymphocyte infusion (DLI) or infusion of cytotoxic T lymphocytes (CTL) up to 1 year after transfusion;  d) In case of patients with prolonged T-cell depletion after medication:  - Fludarabine or other T-cell depleting therapy as indicated by the pharmacist (up to 6 months after discontinuation of the therapy). | - | EXPERT opinion |
| Recommendation 6.1.4. We believe that irradiated blood products should be used in case of patients that receive CAR-T cell therapy from 4 weeks before the leukapheresis until 1 year after the infusion. Unless otherwise described in the study protocol. | - | EXPERT opinion |
| Low or high-volume red blood cell transfusions in children with cancer | | |
| Recommendation 7.1.1. We suggest a transfusion volume of 10-15 ml/kg in children with cancer. | WEAK | VERY LOW QUALITY evidence |
| Recommendation 7.1.2. We suggest *against* a transfusion volume of 20 ml/kg or higher in children with cancer. | WEAK | VERY LOW QUALITY evidence |
| Recommendation 7.1.3. We suggest a transfusion volume with a maximum of 2 donor units (between 500-600 ml) per anemic episode. | - | EXPERT opinion |
| Infusion rates of red blood cell transfusions in children with cancer | | |
| Recommendation 8.1.1. We believe that the infusion rate of a red blood cell (RBC) transfusion should be 5ml/kg/hour in children with cancer, with a minimum of 3 hours. | - | EXPERT opinion |

*The color-coding in this table emphasizes the strength of the recommendation and shows if something is advised (green or yellow) or discouraged (orange or red).

**Table 1b.** Overview of red blood cell transfusion recommendations for children and neonates with cancer

| Recommendation | Strength of recommendation | Quality of evidence |
| --- | --- | --- |
| Neonates with cancer | | |
| Recommendation 1.2.1. We suggest a hemoglobin (Hb) threshold of 6.5 mmol/L for red blood cell (RBC) transfusion in neonates with cancer when they are less than 1 week old. | WEAK | VERY LOW QUALITY evidence |
| Recommendation 1.2.2. We suggest a hemoglobin (Hb) threshold of 5.5 mmol/L for red blood cell (RBC) transfusion in neonates with cancer when they are between 1 and 3 weeks old. | WEAK | VERY LOW QUALITY evidence |
| Recommendation 1.2.3. We suggest a hemoglobin (Hb) threshold of 4.5 mmol/L for red blood cell (RBC) transfusion in neonates with cancer when they are between 3 and 4 weeks old. | WEAK | VERY LOW QUALITY evidence |
| Neonates with cancer during sepsis | | |
| Recommendation 2.2.1. We suggest a hemoglobin (Hb) threshold of 6.5 mmol/L for red blood cell (RBC) transfusion in neonates with cancer during sepsis when they are less than 1 week old. | WEAK | VERY LOW QUALITY evidence |
| Recommendation 2.2.2. We suggest a hemoglobin (Hb) threshold for red blood cell (RBC) transfusion of 5.5 mmol/L in neonates with cancer during sepsis when they are between 1 and 3 weeks old. | WEAK | VERY LOW QUALITY evidence |
| Recommendation 2.2.3. We suggest a hemoglobin (Hb) threshold for red blood cell (RBC) transfusion of 4.5 mmol/L in neonates with cancer during sepsis when they are between 3 and 4 weeks old. | WEAK | VERY LOW QUALITY evidence |
| Neonates with cancer who undergo radiotherapy | | |
| Recommendation 3.2.1. We believe a hemoglobin (Hb) threshold of 6.5 mmol/L for red blood cell (RBC) transfusion should be maintained in neonates with cancer who undergo radiotherapy when they are less than 1 week old. | - | EXPERT opinion |
| Recommendation 3.2.2. We believe a hemoglobin (Hb) threshold for red blood cell (RBC) transfusion of 5.5 mmol/L should be maintained in neonates with cancer who undergo radiotherapy when they are between 1 and 3 weeks old. | - | EXPERT opinion |
| Recommendation 3.2.3. We believe a hemoglobin (Hb) threshold for red blood cell (RBC) transfusion of 4.5 mmol/L should be maintained in neonates with cancer who undergo radiotherapy when they are between 3 and 4 weeks old. | - | EXPERT opinion |
| Neonates with cancer with cardiac and/or pulmonary comorbidities | | |
| Recommendation 4.2.1. We suggest a hemoglobin (Hb) threshold of 7.5 mmol/L for red blood cell (RBC) transfusion in neonates with cancer and cardiac and pulmonary comorbidities when they are less than 1 week old. | WEAK | VERY LOW QUALITY evidence |
| Recommendation 4.2.2. We suggest a hemoglobin (Hb) threshold of 6.5 mmol/L for red blood cell (RBC) transfusion in neonates with cancer and cardiac and pulmonary comorbidities when they are between 2 and 3 weeks old. | WEAK | VERY LOW QUALITY evidence |
| Recommendation 4.2.3. We suggest a hemoglobin (Hb) threshold of 5.5 mmol/L for red blood cell (RBC) transfusion in neonates with cancer and cardiac and pulmonary comorbidities when they are between 3 and 4 weeks old. | WEAK | VERY LOW QUALITY evidence |
| Neonates with cancer during hyperleukocytosis | | |
| Recommendation 5.2.1. In neonates with cancer and hyperleukocytosis, we believe that a RBC transfusion should be given with restraint until the number of leukocytes has fallen below 100 x 109 /L or in the presence of clinical symptoms of hyperleukocytosis. | - | EXPERT opinion |
| Recommendation 5.2.2. In neonates with cancer and hyperleukocytosis, we believe that a RBC transfusion should be given with restraint unless there are severe clinical signs of anemia or in case of an Hb below 5.5 mmol/L in neonates with cancer when they are less than 1 week old. | - | EXPERT opinion |
| Recommendation 5.2.3. In neonates with cancer and hyperleukocytosis, we believe that a RBC transfusion should be given with restraint unless there are severe clinical signs of anemia or in case of an Hb below 4.5 mmol/L for RBC transfusion in neonates with cancer when they are between 1 and 3 weeks old. | - | EXPERT opinion |
| Recommendation 5.2.4. In neonates with cancer and hyperleukocytosis, we believe that a RBC transfusion should be given with restraint unless there are severe clinical signs of anemia or in case of an Hb below 3.5 mmol/L for RBC transfusion in neonates with cancer when they are between 3 and 4 weeks old. | - | EXPERT opinion |
| Recommendation 5.2.5. If needed, transfuse with a maximum of 5 ml/kg/4-6 hours. | - | EXPERT opinion |
| Irradiated red blood cell transfusions in neonates with cancer | | |
| Recommendation 6.2.1. We believe that irradiated blood products should be used in case of an HLA related product and donor: a) Transfusion between 1st to 3rd degree relatives of cell-containing blood products;  b) HLA-compatible platelet concentrates. | - | EXPERT opinion |
| Recommendation 6.2.2. We believe that irradiated blood products should be used in case of granulocyte transfusions. | - | EXPERT opinion |
| Recommendation 6.2.3. We believe that irradiated blood products should be used depending on the patient's immune status:  a) During intrauterine transfusions until 6 months after the due date;  b) Children with congenital combined immune deficiencies (e.g. SCID);  c) Acquired immune deficiencies such as:  - Allogeneic stem cell transplantations up to 1 year after transplantation;  - Autologous stem cell transplantations up to 6 months after transplantation;  - After application of donor lymphocyte infusion (DLI) or infusion of cytotoxic T lymphocytes (CTL) up to 1 year after transfusion;  d) In case of patients with prolonged T-cell depletion after medication:  - Fludarabine or other T-cell depleting therapy as indicated by the pharmacist (up to 6 months after discontinuation of the therapy). | - | EXPERT opinion |
| Recommendation 6.2.4. We believe that irradiated blood products should be used in case of patients that receive CAR-T cell therapy from 4 weeks before the leukapheresis until 1 year after the infusion. Unless otherwise described in the study protocol. | - | EXPERT opinion |
| Low or high-volume red blood cell transfusions in neonates with cancer | | |
| Recommendation 7.2.1. We suggest a transfusion volume of 10-15 ml/kg in neonates with cancer. | WEAK | VERY LOW QUALITY evidence |
| Recommendation 7.2.2. We suggest *against* a transfusion volume of 20 ml/kg or higher in neonates with cancer. | WEAK | VERY LOW QUALITY evidence |
| Infusion rates of red blood cell transfusions in neonates with cancer | | |
| Recommendation 8.2.1. We believe that the infusion rate of a red blood cell (RBC) transfusion should be 5ml/kg/hour in neonates with cancer. | - | EXPERT opinion |

*The color-coding in this table emphasizes the strength of the recommendation and shows if something is advised (green or yellow) or discouraged (orange or red).
