# Supplementary material for: Prophylactic red blood cell transfusions in children and neonates with cancer: An evidence-based clinical practice guideline
Source: Support Care Cancer. 2024 Nov 4;32(11):766. doi: 10.1007/s00520-024-08888-3 (PMC11534970; doi:10.1007/s00520-024-08888-3)
Supplement: Supplementary file 9 — Supplementary file9 (DOCX 753 KB) [file 520_2024_8888_MOESM9_ESM.docx]

**Supplemental materials S9: Supporting materials**

**1. PROPHYLACTIC RED BLOOD CELL TRANSFUSION IN GENERAL
1.1 PROPHYLACTIC RED BLOOD CELL TRANSFUSION IN CHILDREN WITH CANCER
A. Primary search**

**Table 1.** Evidence table of the primary included studies.

| **Outcome reported in study** | **Author, study design** | **No. of participants, total (cases vs controls) & Group definition** | **Results (outcome: infections, number of patients infected, infection rates etc.)** | **Statistical methods** | **Effect size** | **Quality of evidence** |
| --- | --- | --- | --- | --- | --- | --- |
| **Mortality**  *100-day mortality* | 1. Lightdale,  Pre-post trial | 1. 141 (66 vs 75), Children with cancer. Pre: routine RBC transfusion <5.6 mmol/L. Post: routine RBC transfusion <4.3 mmol/L | 1. Pre: Total 17 (25.8%), relapse related: 9 (13.6%), transplantation related 8 (12.1%). Post: Total 13 (17.3%), relapse related: 6 (8.0%), transplantation related 7 (9.3%). | 1. Wilcoxon rank-sum test  **Total Risk Ratio (95% CI)** | 1. *p*=.22  **RR 0.67  (0.35 - 1.28)** | ⨁◯◯◯^A^ VERY LOW |
| **Quality of life** | - | - | - | - | - | - |
| **Transfusion- related complications**  *Incidence of VOD* | 1. Robitaille, RCT | 1. 6 (3 vs 3), Children with cancer. Control >4.3 mmol/L, experimental >7.5 mmol/L. | 1. control: 0, experimental: 3. | 1. Unilateral Fisher exact test  **Total Risk Ratio (95% CI)** | 1. *p*=.05  **RR 7.00 (0.51 - 96.06)** | ⨁⨁◯◯^B^  LOW |
| **Anti-cancer treatment related complications**  *Deferred chemotherapy* | 1. Smith, RCT  2. Toogood, RCT | 1. 27 (16 vs 11), Children with cancer. Control: 6.2-7.5 mmol/L, experimental: 8.69-9.93 mmol/L.  2. 26 (13 vs 13), Children with cancer. Control: 6.2-7.5 mmol/L, experimental: 9.93-11.17 mmol/L | 1. control 7/16, experimental 0/11  2. control 5, experimental 0 | 1. Chi squared  **Total Risk Ratio (95% CI)**  2. non-parametric Wilcoxon rank-sum test and the X2 test with Yates’ correction  **Total Risk Ratio (95% CI)** | 1. .02<*p*<.05  **RR 0.09**  **(0.01 - 1.49**  2. .02<*p*<.05  **RR = 0.09**  **(0.01 - 1.49)** | ⨁⨁◯◯^C1^ LOW  ⨁⨁◯◯^C2^ LOW |
| **Morbidity**  *Incidence of infections* | 1. Toogood, RCT  2. Smith, RCT | 1. 26 (13 vs 13), Children with cancer. Control: 6.2-7.5 mmol/L, experimental: 9.93-11.17 mmol/L  2. 27 (16 vs 11), Children with cancer. Control: 6.2-7.5 mmol/L, experimental: 8.69-9.93 mmol/L. | 1. Control: Total 7, at presentation 3, acquired 4. Experimental: Total 5, at presentation 4, acquired 1  2. Control 11, experimental 1 | 1. Wilcoxon rank-sum test & x2 test with Yates’s correction  **Total Risk Ratio (95% CI)**  2. Chi squared  **Total Risk Ratio (95% CI)** | 1. 0.3<*p*<0.5  **RR 0.71 (0.30 - 1.67)**  2. .02<*p*<.05  **RR 0.13 (0.02 - 0.88)** | ⨁⨁◯◯^D1^  LOW  ⨁⨁◯◯^D2^  LOW |
| **Event-free survival** | - | - | - | - | - | - |
| **Admission to hospital**  *Length of stay* | 1. Lightdale, Pre-post trial | 1. 141 (66 vs 75), Children with cancer. Pre: routine RBC transfusion <5.6 mmol/L. Post: routine RBC transfusion <4.3 mmol/L | 1. Pre: Median (IQR): 37 (30, 46), Post: Median 37 (29, 51) | 1. Wilcoxon rank-sum test* | 1. *p*=.69* | ⨁◯◯◯^E^  VERY LOW |
| **Late complications** | - | - | - | - | - | - |
| **Costs** | 1. Lightdale, Pre-post trial | 1. 141 (66 vs 75), Children with cancer. Pre: routine RBC transfusion <5.6 mmol/L. Post: routine RBC transfusion <4.3 mmol/L | 1. Pre: Median (IQR): $3624 (2265, $6040). Post: $2185 (1812, 3997). | 1. Wilcoxon rank-sum test* | 1. *p*=.004* | ⨁◯◯◯^F^  VERY LOW |

* Reported from study

*GRADE* assessment of the primary included studies

A. Design is pre-post trial, inconsistency not serious, indirectness not serious, imprecision serious (downgraded one level because of few cases), publication bias unlikely, downgraded 1 level because of serious risk of bias (selection bias low, attrition bias high, detection bias low, reporting bias high, confounding bias low, other bias high).

B. Design is randomized control trial, inconsistency not serious, indirectness not serious, imprecision serious (downgraded one level because of few cases), publication bias unlikely, downgraded 1 level because of serious risk of bias (random sequence generation bias low, allocation concealment unclear, performance bias unclear, detection bias unclear, attrition bias low, reporting bias low, other bias high).

C1. Design is randomized control trial, inconsistency not serious, indirectness not serious, imprecision serious (downgraded one level because of few cases), publication bias unlikely, downgraded 1 level because of serious risk of bias (random sequence generation bias unclear, allocation concealment unclear, performance bias unclear, detection bias unclear, attrition bias low, reporting bias unclear, other bias high).

C2. Design is randomized control trial, inconsistency not serious, indirectness not serious, imprecision serious (downgraded one level because of few cases), publication bias unlikely, downgraded 1 level because of serious risk of bias (random sequence generation bias unclear, allocation concealment unclear, performance bias unclear, detection bias unclear, attrition bias high, reporting bias low, other bias high).

D1. Design is randomized control trial, inconsistency not serious, indirectness not serious, imprecision serious (downgraded one level because of few cases), publication bias unlikely, downgraded 1 level because of serious risk of bias (random sequence generation bias unclear, allocation concealment unclear, performance bias unclear, detection bias unclear, attrition bias high, reporting bias unclear, other bias high).

D2. Design is randomized control trial, inconsistency not serious, indirectness not serious, imprecision serious (downgraded one level because of few cases), publication bias unlikely, downgraded 1 level because of serious risk of bias (random sequence generation bias unclear, allocation concealment unclear, performance bias unclear, detection bias unclear, attrition bias low, reporting bias unclear, other bias high).

E. Design is pre-post study, inconsistency not serious, indirectness not serious, imprecision serious (downgraded one level because of few cases), publication bias unlikely, downgraded 1 level because of serious risk of bias (selection bias low, attrition bias high, detection bias low, reporting bias high, confounding bias low, other bias high).

F. Design is pre-post study, inconsistency not serious, indirectness not serious, imprecision serious (downgraded one level because of few cases), publication bias unlikely, downgraded 1 level because of serious risk of bias (selection bias low, attrition bias high, detection bias low, reporting bias high, confounding bias low, other bias high).

**B. Additional search**

The full description of the additional guidelines and the AGREE II-scores are presented in Supplemental Materials S5.

**Table 2.** Included additional guidelines.

| **Research question 1A - The effect of prophylactic RBC transfusion in children with cancer** | |
| --- | --- |
| Recommendations for pediatric oncology: | **Valentine (2018): Consensus Recommendations for RBC Transfusion Practice in Critically Ill Children From the Pediatric Critical Care Transfusion and Anemia Expertise Initiative. Pediatric Critical Care Medicine (Steiner, 2018).**  *AGREE II assessment: Domain 1 = 89%, Domain 2 = 44%, Domain 3 = 79%, Domain 4 = 78%, Domain 5 = 63%, Domain 6 = 92%, Overall Guideline Assessment: Score 5.*  Recommendations for critically ill children with hematologic and oncologic diagnosis according to Valentine (2018):   - In children with oncologic diagnosis who are critically ill or at risk for critical illness, **an Hb concentration between 4.3 and 5.0 mmol/dL is advised**. Weak recommendation, Low quality pediatric evidence (2C) 88% Agreement, (n=35), Median 8, IQR 7-8.   - Supportive arguments: The recommendation is based on consensus, due to lack of evidence, which is mostly based on adult data (Lacroix, 2007; Hébert, 1999). - In children undergoing a HSCT who are critically ill or at risk for critical illness and are hemodynamically stable, **an Hb concentration between 4.3 and 5.0 mmol/dL is advised**. Weak recommendation, Low quality pediatric evidence (2C) 88% Agreement, (n=35), Median 8, IQR 7-8.   - Supportive arguments: The recommendation is based on consensus, due to lack of evidence, which is mostly based on adult data (Lacroix, 2007; Hébert, 1999). |
|  | **JPAC (2013): Transfusion Handbook**  *AGREE II assessment: Domain 1 = 94%, Domain 2 = 56%, Domain 3 = 35%, Domain 4 = 56%, Domain 5 = 58%, Domain 6 = 25%, Overall Guideline Assessment: Score 4.*  Recommendation according to the Transfusion Handbook from the JPAC (2013):   - **An Hb threshold <4.3 mmol/L** in case of pediatric hemato-oncology patients.   - Supporting arguments: The TRIPICU study showed that a restrictive RBC threshold was safe in hemodynamically stable critically ill children (Lacroix, 2007). |
| Recommendations for pediatrics: | **Valentine (2018): Consensus Recommendations for RBC Transfusion Practice in Critically Ill Children From the Pediatric Critical Care Transfusion and Anemia Expertise Initiative. Pediatric Critical Care Medicine (Doctor, 2018).** *AGREE II assessment: Domain 1 = 89%, Domain 2 = 44%, Domain 3 = 79%, Domain 4 = 78%, Domain 5 = 63%, Domain 6 = 92%, Overall Guideline Assessment: Score 5.*  Recommendations for critically ill children according to Valentine (2018):   - In critically ill children or those at risk for critical illness **an Hb threshold of 3.1 mmol/dL is advised**. Strong recommendation, Low quality pediatric evidence (1C), 100% Agreement, (n=35), Median 9, IQR 8-9.   - Supporting arguments: Several descriptive studies have reported significantly adverse outcomes in hospitalized children with an Hb level of 3.1 mmol/dL. However, the study of Lackritzsuggests that RBC transfusion <3.1 mmol improves survival in patients. (English, 2002; Lackritz, 1992; Lackritz, 1997; Akech, 2008; Olupot-Olupot, 2014) - In critically ill children or those at risk for critical illness, who are hemodynamically stable, **an Hb threshold >4.3 mmol/dL is not advised**. Strong recommendation, Moderate quality pediatric evidence (1B), 97% Agreement, (n=29), Median 9, IQR 8-9.   - Supporting arguments: The TRIPICU study showed that a restrictive RBC threshold was safe in hemodynamically stable critically ill children (Lacroix, 2007). - There is **insufficient evidence** to make a recommendation regarding transfusion thresholds for critically ill children who have **an Hb concentration between 3.1 and 4.3 mmol/dL**. However, it is reasonable to consider transfusion based on clinical judgment in these children. Consensus panel expertise, 100% Agreement, (n=29), Median 9, IQR 9-9 .   - Supporting arguments: The TRIPICU study suggests that a RBC threshold of 4.3 mmol/dL is safe and Lackritzstate that a RBC transfusion should be given in patients with an Hb <3.1 mmol/dL. It is unknown whether a RBC transfusion should be given between 3.1 and 4.3 mmol/dL (Lackritz, 1997; Lacroix, 2007). - In critically ill children or those at risk for critical illness who are hemodynamically stable, it is **recommended** that the **post-transfusion goal is between 4.3 mmol/dL and 5.6 mmol/dL**. Weak recommendation, Low quality pediatric evidence (2C), 96% Agreement, (n=28), Median 8, IQR 8-9.   - Supporting evidence: The TRIPICU study set the post-transfusion Hb goal between 4.3 and 5.6 mmol/dL, but not to increase the Hb to the normal range (>7.5 mmol/dL), which was safe (Lacroix, 2007). |
|  | **British Committee for Standards in Haematology (2016): Guidelines on transfusion for fetuses, neonates and older children.**  *AGREE II assessment: Domain 1 = 83%, Domain 2 = 55%, Domain 3 = 54%, Domain 4 = 83%, Domain 5 = 29%, Domain 6 = 50%, Overall Guideline Assessment: Score 4.*  Recommendations according to New (2016):   - **An Hb threshold <4.3 mmol/L is advised in stable non-cyanotic patients**. 1B recommendation. - **An Hb threshold >4.3 mmol/L may be considered in unstable patients or symptomatic anemia**. 2C recommendation.   - Supporting arguments: Based on the TRIPICU study and others (Lacroix, 2007; Lacroix, 2012; Carson, 2012; BCSH, 2013; Hébert & Carson, 2014; NICE, 2015) |
|  | **NICE (2015): Blood transfusion.**  *AGREE II assessment: Domain 1 = 100%, Domain 2 = 89%, Domain 3 = 69%, Domain 4 = 94%, Domain 5 = 54%, Domain 6 = 92%, Overall Guideline Assessment: Score 5.*  Recommendations according to the NICE (2015) blood transfusion guideline:   - **Consider an Hb threshold of 4.3 mmol/dL** and a hemoglobin concentration target of 4.3–5.6 mmol/dL after transfusion.   - Supporting arguments: This is based on 2 studies (Lacroix, 2007; Cholette, 2011). |
| Recommendations for adults with cancer | **CBO (2011): Guideline transfusions.**  *AGREE II assessment: Domain 1 = 89%, Domain 2 = 94%, Domain 3 = 85%, Domain 4 = 61%, Domain 5 = 50%, Domain 6 = 0%, Overall Guideline Assessment: Score 5.*  Recommendations according to CBO (2011):   - **An Hb <3 mmol/L is an absolute indication** for a RBC transfusion.   - Supporting arguments: Based on an old study in Jehovah’s Witnesses stating that mortality rates increased with an Hb <3 mmol/dL (Viele & Weiskopf, 1994). - Prophylactic RBC transfusions may be indicated for asymptomatic chronic anemia in a **patient without cardiopulmonary limitations and an Hb <4 mmol/L**.   - Supporting arguments: Recommendation is based on consensus. - Prophylactic RBC transfusions may be indicated with **limited cardiopulmonary compensation** options or risk factors according to the **4-5-6 rule**.   - Supporting arguments: Recommendation is based on consensus. - When there are **no clear limited cardiopulmonary compensation option**s or risk factors, an Hb **threshold 3.5-4.5 mmol/dL** may be used for prophylactic RBC transfusions in children and adolescents <25 years.   - Supporting arguments: Recommendation is based on consensus. - In case of **aplasia-inducing treatments** it has been shown that a **restrictive transfusion policy (4.4-5.5 mmol/L)** compared to a more liberal policy (6 mmol/L) did not lead to more adverse patient outcomes.   - Supporting arguments: Based on one study (Jansen, 2004). - In case of **solid tumors**, cancer patients often receive transfusions at **Hb <6 mmol/L**.   - Supporting arguments: Recommendation is based on consensus. - In case of **lymphatic and myeloid leukemias**, there are **no studies** on Hb thresholds for this condition.   - Supporting arguments: none. |
|  | **JPAC (2013): Transfusion Handbook.**  *AGREE II assessment: Domain 1 = 94%, Domain 2 = 56%, Domain 3 = 35%, Domain 4 = 56%, Domain 5 = 58%, Domain 6 = 25%, Overall Guideline Assessment: Score 4.*  Recommendation according to the Transfusion Handbook from the JPAC (2013):   - Consider **an Hb threshold between 5.0 and 5.6 mmol/dL**.   - Supporting arguments: this is what most units in the UK followed, but this is not based on evidence. |

**C. Evidence to Decision Frameworks
Table 3.** Evidence to Decision Framework & Overall conclusions - <3.1 mmol/L versus >3.1 mmol/L.

| **Hb threshold <3.1 mmol/L versus an Hb threshold >3.1 mmol/L** | | | | |
| --- | --- | --- | --- | --- |
|  | **Criteria** | **Judgements** | **Research evidence** | **Additional considerations** |
| **PROBLEM** | Is the problem a priority? | ☐ No  ☐ Probably no  ☐ Uncertain  ☐ Probably yes  ■ **Yes** | RBC transfusions are one of the backbones in the supportive care management of children with oncologic diagnoses and those who are undergoing hematopoietic stem cell transplants (HSCT). Children with cancer may require RBC transfusions due to the underlying oncologic disease or bone marrow suppression as a result of the anti-cancer treatment. This guideline is the first guideline to be established specifically for pediatric oncology. |  |
| **BENEFITS AND HARMS** | What is the overall certainty of this evidence? | ☐ No included studies  **■ Very low**  ☐ Low  ☐ Moderate  ☐ High | Summary of findings:  Mortality - Pediatric oncology: No studies included.  - Pediatric: Lackritz (1997) reported significantly more mortality in the group with an Hb <3.1 mmol/L in comparison to the group with an Hb >3.1 (**RR 1.93 (95% CI 1.36 - 2.74)**). *Evidence cited in existing guidelines.*  - Adult: Viele & Weiskopf (1994), Shander (2014), and Carson (2002) all reported significantly more mortality in the group with an Hb <3.1 mmol/L in comparison to the group with an Hb >3.1 mmol/L (**23 of the 50 reported deaths were primarily due to anemia and died with Hb concentrations <or = 3.1 mmol/L, RR 3.87 (95% CI 1.56 - 9.58)**, and **RR 7.18 (95% CI 3.32 - 15.54)**, respectively). *Evidence cited in existing guidelines.*  *Pooled effect (Shander & Carson):* **RR 5.50 (95% CI 3.08 - 9.83)** |  |
|  | Is there important uncertainty about how much people value the main outcomes? | ☐ Important uncertainty or variability  ☐ Possibly important uncertainty or variability  ☐ Probably no important uncertainty or variability  ■ **No important uncertainty or variability**  ☐ No known undesirable outcomes |  | The relative importance of all outcomes was unanimously determined. |
|  | Are the desirable anticipated effects large? | ☐ No  ☐ Probably no  **■** **Uncertain**  ☐ Probably yes  ☐ Yes  ☐ Varies |  | The desirable effects are unknown e.g. quality of life, hospital admission, costs. |
|  | Are the undesirable anticipated effects small? | ☐ No  ■ **Probably no**  ☐ Uncertain  ☐ Probably yes  ☐ Yes  ☐ Varies |  | The risk of mortality increases significantly in all studies. Therefore, the undesirable anticipated effects are not considered to be small. However, the evidence is mainly based on adult data, thus probably no. |
|  | Are the desirable effects large relative to undesirable effects? | ☐ No  ■ **Probably no**  ☐ Uncertain  ☐ Probably yes  ☐ Yes  ☐ Varies |  | Even though this is purely based on data in adults: the risk of mortality, and thus the most critical undesirable outcome, overrules the uncertain desirable effects. However, this is purely based on mainly adult data, thus probably no. |
| **RESOURCE USE** | Are the resources required small? | ☐ No  ☐ Probably no  ☐ Uncertain  ■ **Probably yes**  ☐ Yes  ☐ Varies |  | The guideline panel decided that the resources necessary are probably small. |
|  | Is the incremental cost small relative to the net benefits? | ☐ No  ☐ Probably no  ■ **Uncertain**  ☐ Probably yes  ☐ Yes  ☐ Varies |  | There are costs involved (e.g., hospital admission costs). However, benefits are considered uncertain. |
| **EQUITY** | What would be the impact on health inequities? | ☐ Increased  ☐ Probably increased  ☐ Uncertain  ☐ Probably reduced  ☐ Reduced  ■ **Varies** |  | The panel expected that implementing this option has no effect on health inequities, given the structure of the Dutch healthcare system. However, in other countries this may vary depending on their healthcare system. |
| **ACCEPTABILITY** | Is the option acceptable to key stakeholders? | ■ **No**  ☐ Probably no  ☐ Uncertain  ☐ Probably yes  ☐ Yes  ☐ Varies |  | The panel considered the option of a transfusion threshold <3.1 mmol/L not acceptable for the key stakeholders, e.g., doctors and parents. |
| **FEASIBILITY** | Is the option feasible to implement? | ☐ No  ☐ Probably no  ☐ Uncertain  ☐ Probably yes  ■ **Yes**  ☐ Varies |  | The panel considered the option of a transfusion threshold <3.1 mol/l feasible to implement. |

| **Balance of consequences – Hb threshold <3.1 mmol/L relative to an Hb threshold >3.1 mmol/L** | | | | |
| --- | --- | --- | --- | --- |
| **Undesirable consequences  *clearly outweigh*  desirable consequences in most settings**  ■ | Undesirable consequences *probably outweigh*  desirable consequences in most settings  ☐ | The balance between  desirable and undesirable consequences  *is closely balanced or uncertain*  ☐ | Desirable consequences  *probably outweigh* undesirable consequences in most settings  ☐ | Desirable consequences  *clearly outweigh*  undesirable consequences in most settings  ☐ |

| **Type of recommendation – Hb threshold <3.1 mmol/L relative to an Hb threshold >3.1 mmol/L** | | | |
| --- | --- | --- | --- |
| **We recommend against**  **offering this option**  ■ | We suggest not offering  this option  ☐ | We suggest offering  this option  ☐ | We recommend offering  this option  ☐ |
| Recommendation (text) | We recommend *against* a hemoglobin (Hb) threshold of 3.1 mmol/L or lower for red blood cell (RBC) transfusion in children with cancer (Strong recommendation). | | |
| Justification | Several descriptive studies have all reported significantly more mortality in hospitalised adults and children with an Hb level of 3.1 mmol/dL (Carson, 2002; Lackritz, 1997; Viele & Weiskopf, 1994; Shander, 2014). Although the level of evidence is low and from mainly adult studies, we recommend against offering this option considering the severe adverse event, death. There are no studies reporting any potential benefit from an Hb threshold <3.1 mmol/L. In addition, this option is considered not acceptable for all stakeholders. | | |
| Subgroup considerations | No subgroup considerations were formulated. | | |
| Implementation considerations | No implementation considerations were formulated. | | |
| Monitoring and evaluation | Not applicable. | | |
| Research priorities | See supplemental materials S9 "Gaps in research". | | |

**Table 4.** Evidence to Decision Framework & Overall conclusions - <3.7 mmol/L versus >3.7 mmol/L.

| **Hb threshold <3.7 mmol/L versus an Hb threshold >3.7 mmol/L** | | | | |
| --- | --- | --- | --- | --- |
|  | **Criteria** | **Judgements** | **Research evidence** | **Additional considerations** |
| **PROBLEM** | Is the problem a priority? | ☐ No  ☐ Probably no  ☐ Uncertain  ☐ Probably yes  ■ **Yes** | RBC transfusions are one of the backbones in the supportive care management of children with oncologic diagnoses and those who are undergoing hematopoietic stem cell transplants (HSCT). Children with cancer may require RBC transfusions due to the underlying oncologic disease or bone marrow suppression as a result of the anti-cancer treatment. This guideline is the first guideline to be established specifically for pediatric oncology. |  |
| **BENEFITS AND HARMS** | What is the overall certainty of this evidence? | ☐ No included studies  **■ Very low**  ☐ Low  ☐ Moderate  ☐ High | Summary of findings:  Mortality - Pediatric oncology: No studies included.  - Pediatric: No studies included.  - Adults: Shander (2014) reported significantly more mortality in the group with an Hb threshold <3.1 mmol/L in comparison to the group with an Hb threshold >3.1 mmol/L, **RR 5.46 (95% CI 1.81 - 16.46)**. However, Carson (2002) reported no significant difference, RR 2.87 (95% CI 0.86 - 9.53). *Evidence cited in existing guidelines.*  Pooled estimate: **RR 4.01 (95% CI 1.80 - 8.95)** |  |
|  | Is there important uncertainty about how much people value the main outcomes? | ☐ Important uncertainty or variability  ☐ Possibly important uncertainty or variability  ☐ Probably no important uncertainty or variability  ■ **No important uncertainty or variability**  ☐ No known undesirable outcomes |  | The relative importance of all outcomes was unanimously determined. |
|  | Are the desirable anticipated effects large? | ☐ No  ☐ Probably no  **■ Uncertain**  ☐ Probably yes  ☐ Yes  ☐ Varies |  | The desirable effects are unknown e.g., quality of life, hospital admission, costs. |
|  | Are the undesirable anticipated effects small? | ☐ No  ■ **Probably no**  ☐ Uncertain  ☐ Probably yes  ☐ Yes  ☐ Varies |  | The pooled risk of mortality was statistically different. Thus, the undesirable anticipated effects are not small. However, considering that this is based on adult data and on pooled data the risks are probably not small. |
|  | Are the desirable effects large relative to undesirable effects? | ☐ No  **■ Probably no**  ☐ Uncertain  ☐ Probably yes  ☐ Yes  ☐ Varies |  | Even though this is purely based on data in adults: the risk of mortality, and thus the most critical undesirable outcome, overrules the uncertain desirable effects. However, this is purely based on pooled adult data, thus probably no. |
| **RESOURCE USE** | Are the resources required small? | ☐ No  ☐ Probably no  ☐ Uncertain  ■ **Probably yes**  ☐ Yes  ☐ Varies |  | The guideline panel decided that the resources necessary are probably small. |
|  | Is the incremental cost small relative to the net benefits? | ☐ No  ☐ Probably no  ■ **Uncertain**  ☐ Probably yes  ☐ Yes  ☐ Varies |  | There are costs involved (e.g., hospital admission costs). However, benefits are considered uncertain. |
| **EQUITY** | What would be the impact on health inequities? | ☐ Increased  ☐ Probably increased  ☐ Uncertain  ☐ Probably reduced  ☐ Reduced  ■ **Varies** |  | The panel expected that implementing this option has no effect on health inequities, given the structure of the Dutch healthcare system. However, in other countries this may vary depending on their healthcare system. |
| **ACCEPTABILITY** | Is the option acceptable to key stakeholders? | ☐ No  ■ **Probably no**  ☐ Uncertain  ☐ Probably yes  ☐ Yes  ☐ Varies |  | The panel considered the option of a transfusion threshold <3.7 mmol/L probably not acceptable for the key stakeholders, e.g., doctors and parents. However, more acceptable than <3.1 mmol/L. |
| **FEASIBILITY** | Is the option feasible to implement? | ☐ No  ☐ Probably no  ☐ Uncertain  ☐ Probably yes  ■ **Yes**  ☐ Varies |  | The panel considered the option of a transfusion threshold <3.7 mol/l feasible to implement. |

| **Balance of consequences – Hb threshold <3.7 mmol/L relative to an Hb threshold >3.7 mmol/L** | | | | |
| --- | --- | --- | --- | --- |
| Undesirable consequences  *clearly outweigh*  desirable consequences in most settings  ☐ | **Undesirable consequences *probably outweigh*  desirable consequences in most settings**  ■ | The balance between  desirable and undesirable consequences  *is uncertain*  ☐ | Desirable consequences  *probably outweigh* undesirable consequences in most settings  ☐ | Desirable consequences  *clearly outweigh*  undesirable consequences in most settings  ☐ |

| **Type of recommendation – Hb threshold <3.7 mmol/L relative to an Hb threshold >3.7 mmol/L** | | | |
| --- | --- | --- | --- |
| We recommend against  offering this option  ☐ | **We suggest not offering**  **this option**  ■ | We suggest offering  this option  ☐ | We recommend offering  this option  ☐ |
| Recommendation (text) | We suggest *against* a hemoglobin (Hb) threshold of 3.7 mmol/L for red blood cell (RBC) transfusion in children with cancer (Weak recommendation). | | |
| Justification | The pooled results report a significantly increased mortality risk with an Hb of 3.7 mmol/L in comparison to an Hb greater than 3.7 mmol/L in adult patients (Shander, 2014; Carson, 2002). Moreover, there are no studies reporting any potential benefit from an Hb of 3.7 mmol/L, and thus the guideline panel decided that the risk of mortality, the most critical outcome, overrules the uncertain desirable effects. In addition, this option is considered probably not acceptable for all stakeholders. Therefore, the guideline panel decided against recommending an Hb threshold of 3.7 mmol/L in children with cancer. | | |
| Subgroup considerations | No subgroup considerations were formulated. | | |
| Implementation considerations | No implementation considerations were formulated. | | |
| Monitoring and evaluation | Not applicable. | | |
| Research priorities | See supplemental materials S9 "Gaps in research". | | |

**Table 5.** Evidence to Decision Framework & Overall conclusions - <4.3 mmol/L versus >4.3 mmol/L.

| **Hb threshold <4.3 mmol/L versus an Hb threshold >4.3 mmol/L** | | | | |
| --- | --- | --- | --- | --- |
|  | **Criteria** | **Judgements** | **Research evidence** | **Additional considerations** |
| **PROBLEM** | Is the problem a priority? | ☐ No  ☐ Probably no  ☐ Uncertain  ☐ Probably yes  ■ **Yes** | RBC transfusions are one of the backbones in the supportive care management of children with oncologic diagnoses and those who are undergoing hematopoietic stem cell transplants (HSCT). Children with cancer may require RBC transfusions due to the underlying oncologic disease or bone marrow suppression as a result of the anti-cancer treatment. This guideline is the first guideline to be established specifically for pediatric oncology. |  |
| **BENEFITS AND HARMS** | What is the overall certainty of this evidence? | ☐ No included studies  ■ **Very low**  ☐ Low  ☐ Moderate  ☐ High | Summary of findings:  Mortality - Pediatric oncology: Lightdale (2012) reported no significant differences regarding mortality when comparing an Hb threshold <4.3 mmol/L with an Hb threshold <5.6 mmol/L, RR 0.67 (0.35 - 1.28). *Quality of evidence: Very low.* - Pediatric: Lacroix (2007) reported no significant differences regarding mortality when comparing an Hb threshold <4.3 mmol/L with an Hb threshold <5.0 mmol/L, RR 0.99 (95% CI 0.48 - 2.04). *Evidence cited in existing guidelines.*  - Adult: Shander (2014) reported no significant difference regarding mortality when comparing an Hb threshold <4.3 mmol/L with an Hb threshold >4.3 mmol/L, RR 3.44 (95% CI 0.59 - 20.04). However, Carson (2002) reported more mortality in the group with an Hb <4.3 mmol/L in comparison to an Hb >4.3 mmol/L, **RR 19.30 (95% CI 1.09 - 342.66)**. However, Carson (2012) reported less mortality in the group with an Hb threshold <4.3 mmol/L in comparison to an Hb threshold <5.6 mmol/L, **RR 0.75 (95% CI 0.59 - 0.96)**. When comparing an Hb threshold <4.3 mmol/L with <6.2 mmol/L Hébert (1999) found no significant difference, RR 0.79 (95% CI 0.63 - 1.00). *Evidence cited in existing guidelines.*   Quality of life  - Pediatric oncology: no included studies.  - Pediatric: no included studies.  - Adult: Carson (2012) reported no significant differences regarding quality of life when comparing an Hb threshold <4.3 mmol/L with <5.6 mmol/L. *Evidence cited in existing guidelines.*  Transfusion-related complications - Pediatric oncology: Robitaille (2013) reported no significant differences regarding transfusion-related complications when comparing an Hb threshold <4.3 mmol/L with <7.5 mmol/L, RR 7.00 (95% CI 0.51 - 96.06). *Quality of evidence: Low.*  - Pediatric: no included studies.  - Adult: no included studies.  Morbidity - Pediatric oncology: no included studies.  - Pediatric: Lacroix (2007) reported no significant differences regarding morbidity when comparing an Hb threshold <4.3 mmol/L with an Hb threshold <5.0 mmol/L, RR 0.97 (95% CI 0.63 - 1.47). *Evidence cited in existing guidelines.*  - Adult: Carson (2012) and Rohde (2014) reported significantly fewer infections when comparing an Hb threshold <4.3 mmol/L with an Hb threshold <5.6 mmol/L, **RR 0.81 (95% CI 0.67 - 0.98)** and **RR 0.83 (95% CI 0.72 - 0.96)**, other outcomes were not significant. Hébert (1999) reported no significant differences regarding morbidity when comparing an Hb threshold <4.3 mmol/L with an Hb threshold <6.2 mmol/L, RR 1.23 (95% CI 0.67 - 2.26). *Evidence cited in existing guidelines.*  Admission to hospital  - Pediatric oncology: Lightdale (2012) reported no significant differences regarding admission to hospital when comparing an Hb threshold <4.3 mmol/L with an Hb threshold <5.6 mmol/L. *Quality of evidence: Very low.* - Pediatric: Lacroix (2007) reported no significant differences regarding admission to hospital when comparing an Hb threshold <4.3 mmol/L with an Hb threshold <5.0 mmol/L, MD -0.46 (95% CI -0.70 - 1.70). *Evidence cited in existing guidelines.*  - Adult: Carson (2012) reported no significant differences regarding admission to hospital when comparing an Hb threshold <4.3 mmol/L with an Hb threshold <5.6 mmol/L, MD 0.11 (95% CI -0.16 - 0.13). Hébert (1999) reported no significant differences regarding admission to hospital when comparing an Hb threshold <4.3 mmol/L with an Hb threshold <6.2 mmol/L, MD -0.70 (95% CI -3.37 - 1.97). *Evidence cited in existing guidelines.*  Costs  - Pediatric oncology: Lightdale (2012) reported significantly less costs in the group with the Hb threshold <4.3 mmol/L in comparison to the group with an Hb threshold <5.6 mmol/L, ***p*=.004**. *Quality of evidence: Very low.*  - Pediatric: no included studies.  - Adult: no included studies. | The quality of the pediatric oncology studies was considered *very low*. |
|  | Is there important uncertainty about how much people value the main outcomes? | ☐ Important uncertainty or variability  ☐ Possibly important uncertainty or variability  ☐ Probably no important uncertainty or variability  ■ **No important uncertainty or variability**  ☐ No known undesirable outcomes |  | The relative importance of all outcomes was unanimously determined. |
|  | Are the desirable anticipated effects large? | ☐ No  ☐ Probably no  ■ **Uncertain**  ☐ Probably yes  ☐ Yes  ☐ Varies |  | There was no significant difference regarding quality of life and admission to hospital and there was a significant reduction of costs. However, these studies are of low quality. The expert panel decided that the benefits of an Hb threshold <4.3 mmol/L are uncertain considering the gap in evidence. |
|  | Are the undesirable anticipated effects small? | ☐ No  ☐ Probably no  ☐ Uncertain  ■ **Probably yes**  ☐ Yes  ☐ Varies |  | There was no significant difference regarding mortality in most studies, except for 2 adult studies who showed contradictory results (Carson 2002; Carson 2012). There was no significant difference regarding morbidity except for 2 adult studies that reported a significant decrease in the risk of infections. There was no significant difference regarding transfusion-related complications (Robitaille, 2013), however this study was stopped prematurely considering that in the group with the higher Hb threshold three out of three children with cancer developed veno-occlusive disease. |
|  | Are the desirable effects large relative to undesirable effects? | ☐ No  ☐ Probably no  ☐ Uncertain  ■ **Probably yes**  ☐ Yes  ☐ Varies |  | Desirable effects are uncertain except for costs and the undesirable effects are probably small. Therefore, the panel agreed that the desirable effects are probably large relative to the undesirable effects. |
| **RESOURCE USE** | Are the resources required small? | ☐ No  ☐ Probably no  ☐ Uncertain  ■ **Probably yes**  ☐ Yes  ☐ Varies |  | The guideline panel decided that the resources necessary are probably small. |
|  | Is the incremental cost small relative to the net benefits? | ☐ No  ☐ Probably no  ■ **Uncertain**  ☐ Probably yes  ☐ Yes  ☐ Varies |  | There are costs involved (e.g., hospital admission costs). However, benefits are considered uncertain. |
| **EQUITY** | What would be the impact on health inequities? | ☐ Increased  ☐ Probably increased  ☐ Uncertain  ☐ Probably reduced  ☐ Reduced  ■ **Varies** |  | The panel expected that implementing this option has no effect on health inequities, given the structure of the Dutch healthcare system. However, in other countries this may vary depending on their healthcare system. |
| **ACCEPTABILITY** | Is the option acceptable to key stakeholders? | ☐ No  ☐ Probably no  ☐ Uncertain  ■ **Probably yes**  ☐ Yes  ☐ Varies |  | The panel considered the option of a transfusion threshold <4.3 mmol/L acceptable for the key stakeholders, e.g., doctors and parents. However, it is unknown if some parties might find it not acceptable, thus probably yes. |
| **FEASIBILITY** | Is the option feasible to implement? | ☐ No  ☐ Probably no  ☐ Uncertain  ☐ Probably yes  ■ **Yes**  ☐ Varies |  | The panel considered the option of a transfusion threshold <4.3 mol/l feasible to implement. |

| **Balance of consequences – Hb threshold <4.3 mmol/L relative to an Hb threshold >4.3 mmol/L** | | | | |
| --- | --- | --- | --- | --- |
| Undesirable consequences  *clearly outweigh*  desirable consequences in most settings  ☐ | Undesirable consequences *probably outweigh*  desirable consequences in most settings  ☐ | The balance between  desirable and undesirable consequences  *is closely balanced*  ☐ | **Desirable consequences  *probably outweigh* undesirable consequences in most settings**  ■ | Desirable consequences  *clearly outweigh*  undesirable consequences in most settings  ☐ |

| **Type of recommendation – Hb threshold <4.3 mmol/L relative to an Hb threshold >4.3 mmol/L** | | | |
| --- | --- | --- | --- |
| We recommend against  offering this option  ☐ | We suggest not offering  this option  ☐ | **We suggest offering**  **this option**  **■** | We recommend offering  this option  ☐ |
| Recommendation (text) | We suggest a hemoglobin (Hb) threshold of 4.3 mmol/L for red blood cell (RBC) transfusion in children with cancer (Weak recommendation). | | |
| Justification | Two pediatric oncology studies, one pediatric non-cancer study, and five adult non-cancer studies were identified. Based on the evidence, there is no significant increased risk for mortality, morbidity, and transfusion-related complications with an Hb of 4.3 mmol/L in comparison to an Hb greater than 4.3 mmol/L in children with cancer, children in general and adults in seven out of nine studies. However, one study did show significantly more mortality (Carson, 2002) and one study reported less mortality (Carson, 2012). The panel therefore concluded that likely there is no significant difference (Lightdale, 2012; Robitaille, 2013; Lacroix, 2007; Shander, 2014; Carson, 2002; Carson, 2012; Hébert, 1999; Rohde, 2014). And two studies reported less infections with an Hb of 4.3 mmol/L in comparison to an Hb greater than 4.3 mmol/L (Carson, 2012; Rohde, 2014). Moreover, there are no other studies reporting any significant potential benefit from a higher Hb threshold (Rohde, 2014; Lightdale, 2012; Lacroix, 2007; Lacroix, 2012; Carson, 2012). In addition, all of these studies are considered of low quality. Based on this, the guideline panel decided that the benefits of attaining an Hb threshold of 4.3 mmol/L are probably large relative to an Hb threshold greater than 4.3 mmol/L. In addition, this option is considered probably acceptable for all stakeholders. Studies that included higher restrictive Hb thresholds than an Hb threshold of 4.3 mmol/L did not report significant outcomes regarding mortality, morbidity, quality of life, admission to hospital, and anti-cancer treatment-related complications (Jansen, 2004; Carson, 2011). Therefore, the guideline panel decided to suggest an Hb threshold of 4.3 mmol/L in children with cancer. | | |
| Subgroup considerations | However, it is reasonable to consider different transfusion thresholds based on clinical judgment in these children. For instance, in case of unstable children with cancer during sepsis, a higher Hb threshold should be maintained. | | |
| Implementation considerations | No implementation considerations were formulated. | | |
| Monitoring and evaluation | Not applicable. | | |
| Research priorities | See supplemental materials S9 "Gaps in research". | | |

**1.2 PROPHYLACTIC RED BLOOD CELL TRANSFUSION IN NEONATES WITH CANCER
A. Primary search**No supporting materials.

**B. Additional search**

The full description of the additional guidelines and the AGREE II-scores are presented in Supplemental Materials S5.

**Table 1.** Included additional guidelines.

| **Research question 1B - The effect of prophylactic RBC transfusion in neonates with cancer** | |
| --- | --- |
| Recommendations for neonates | **Federation of Medical Specialists (2019): Blood transfusion policy.**  *AGREE II assessment: Domain 1 = 94%, Domain 2 = 100%, Domain 3 = 71%, Domain 4 = 89%, Domain 5 = 13%, Domain 6 = 100%, Overall Guideline Assessment: Score 6.*  Recommendations according to the Federation of Medical Specialists (2019):   - That very low birth weight infants (birth weight <1500 grams) should receive these restrictive RBC transfusions thresholds and in absence of studies with regard to full term neonates and late preterm infants (gestational age>32 weeks), these recommendations are also applying to these groups:   - Maintain **an Hb >6.5 mmol/L in neonates <1 week old**.   - Maintain **an Hb >5.5 mmol/L in neonates between 2 and 3 weeks old**.   - Maintain **an Hb >4.5 mmol/L in neonates >3 weeks old**.     - Supporting arguments: Based on 4 studies and 1 Cochrane-analysis (Connelly, 1999; Bell, 2005; Kirpalani, 2006; Chen, 2009; Whyte & Kirpalani, 2011). |
|  | **JPAC (2013): Transfusion Handbook**  *AGREE II assessment: Domain 1 = 94%, Domain 2 = 56%, Domain 3 = 35%, Domain 4 = 56%, Domain 5 = 58%, Domain 6 = 25%, Overall Guideline Assessment: Score 4.*  The Transfusion Handbook from the JPAC (2013) advises a neonatal top-up transfusion threshold:   - In case of neonates without oxygen:   - Maintain **an Hb >6.2 mmol/L in neonates <24 hours old**.   - Maintain **an Hb >6.2 mmol/L in neonates <1 week old**.   - Maintain **an Hb >4.65 - 5.28 mmol/L depending on clinical situation in neonates between 2-3 weeks old**.   - Maintain **an Hb >4.65 - 5.28 mmol/L depending on clinical situation in neonates >4 weeks old**.     - Supporting arguments: Based on the British Committee for Standards in Haematology Transfusion Guidelines for Neonates and Older Children (New, 2016). |
|  | **British Committee for Standards in Haematology (2016): Guidelines on transfusion for fetuses, neonates and older children.**  *AGREE II assessment: Domain 1 = 83%, Domain 2 = 55%, Domain 3 = 54%, Domain 4 = 83%, Domain 5 = 29%, Domain 6 = 50%, Overall Guideline Assessment: Score 4.*  New (2016) advises a neonatal top-up transfusion threshold:   - In case of neonates without oxygen:   - Maintain **an Hb >6.2 mmol/L in neonates <24 hours old**.   - Maintain **an Hb >6.2 mmol/L in neonates <1 week old**.   - Maintain **an Hb >4.65 - 5.28 mmol/L depending on clinical situation in neonates between 2-3 weeks old**.   - Maintain **an Hb >4.65 - 5.28 mmol/L depending on clinical situation in neonates >4 weeks old**.     - Supporting arguments: Based on studies (Whyte & Kirpalani, 2006; Chen, 2009, Bell, 2005; Whyte & Kirpalani, 2011; Venkatesh, 2012). |

**2. PROPHYLACTIC RED BLOOD CELL TRANSFUSION - SEPSIS
2.1 PROPHYLACTIC RED BLOOD CELL TRANSFUSION IN CHILDREN WITH CANCER ANS SEPSIS**

**A. Primary search**No supporting materials.

**B. Additional search**

The full description of the additional guidelines and the AGREE II-scores are presented in Supplemental Materials S5.

**Table 1.** Included additional guidelines.

| **Research question 2A - The effect of prophylactic RBC transfusion in children with cancer who suffer from sepsis** | |
| --- | --- |
| Recommendations for children with sepsis | **Valentine (2018): Consensus Recommendations for RBC Transfusion Practice in Critically Ill Children From the Pediatric Critical Care Transfusion and Anemia Expertise Initiative. Pediatric Critical Care Medicine (Muszynski, 2018).**  *AGREE II assessment: Domain 1 = 89%, Domain 2 = 44%, Domain 3 = 79%, Domain 4 = 78%, Domain 5 = 63%, Domain 6 = 92%, Overall Guideline Assessment: Score 5.*  Recommendations for critically ill children with a non-hemorrhagic shock, such as a septic shock according to Valentine (2018):   - In **hemodynamically stable** critically ill children with a diagnosis of severe sepsis or septic shock, they **recommended not administering a RBC transfusion if the Hb concentration is ≥ 4.3 mmol/dL.** - Weak recommendation, Low quality pediatric evidence, 96% Agreement, (n=29), Median 8, IQR 8-9.   - Supporting arguments: The recommendation is based on the TRIPICU study and the adult study TRISS stating that an Hb threshold of 4.3 mmol/dL is safe (Lacroix, 2007; Holst, 2014) - In **hemodynamically unstable** critically ill children with a diagnosis of severe sepsis or septic shock and evidence of oxygen deficiency, they suggest **an Hb threshold between 4.3 mmol/dL and 6.2 mmol/dL**. Consensus panel expertise, 100% Agreement, Median 9, IQR 8-9   - Supporting arguments: The recommendation is based on consensus, due to lack of evidence. |

**C. Evidence to Decision Frameworks**

**Table 2.** Evidence to Decision Framework & Overall conclusions - >4.3 mmol/L versus <4.3 mmol/L.

| **Hemoglobin (Hb) threshold >4.3 mmol/L versus a hemoglobin (Hb) threshold of 4.3 mmol/L** | | | | |
| --- | --- | --- | --- | --- |
|  | **Criteria** | **Judgements** | **Research evidence** | **Additional considerations** |
| **PROBLEM** | Is the problem a priority? | ☐ No  ☐ Probably no  ☐ Uncertain  ☐ Probably yes  ■ **Yes** | RBC transfusions are one of the backbones in the supportive care management of children with oncologic diagnoses and those who are undergoing hematopoietic stem cell transplants (HSCT). Children with cancer may require RBC transfusions due to the underlying oncologic disease or bone marrow suppression as a result of the anti-cancer treatment. This guideline is the first guideline to be established specifically for children with cancer during sepsis. |  |
| **BENEFITS AND HARMS** | What is the overall certainty of this evidence? | ☐ No included studies  **■ Very low**  ☐ Low  ☐ Moderate  ☐ High | Summary of findings: Mortality  - Pediatric oncology: no studies included.  - Pediatric: Lacroix (2012) reported no significant differences regarding mortality when comparing an Hb threshold <4.3 mmol/L versus an Hb threshold <5.0 mmol/L, RR 3.45 (95% CI 0.74 - 16.02).  - Adult: Holst (2014) reported no significant differences regarding mortality when comparing an Hb threshold <4.3 mmol/L versus an Hb threshold <5.6 mmol/L, RR 0.96 (95% CI 0.83 - 1.10).  Morbidity  - Pediatric oncology: no included studies.  - Pediatric: Lacroix (2012) reported no significant differences regarding morbidity when comparing an Hb threshold <4.3 mmol/L versus an Hb threshold <5.0 mmol/L RR 0.99 (95% CI 0.49 - 1.97).  - Adult: Holst (2014) reported no significant differences regarding morbidity when comparing an Hb threshold <4.3 mmol/L versus an Hb threshold <5.6 mmol/L, RR 0.33 (95% CI 0.01 - 8.18).  Admission to hospital  - Pediatric oncology: no included studies.  - Pediatric: Lacroix (2012) reported no significant differences regarding admission to hospital when comparing an Hb threshold <4.3 mmol/L versus an Hb threshold <5.0 mmol/L.  - Adult: No pediatric oncology: no included studies. |  |
|  | Is there important uncertainty about how much people value the main outcomes? | ☐ Important uncertainty or variability  ☐ Possibly important uncertainty or variability  ☐ Probably no important uncertainty or variability  ■ **No important uncertainty or variability**  ☐ No known undesirable outcomes |  | The relative importance of all outcomes was unanimously determined. |
|  | Are the desirable anticipated effects large? | ☐ No  ■ **Probably no**  ☐ Uncertain  ☐ Probably yes  ☐ Yes  ☐ Varies |  | There is no significant difference regarding mortality and morbidity. Thus, the undesirable anticipated effects are probably not large. |
|  | Are the undesirable anticipated effects small? | ☐ No  ☐ Probably no  ■ **Uncertain**  ☐ Probably yes  ☐ Yes  ☐ Varies |  | Apart from admission to hospital, there are no other desirable anticipated effects included, thus uncertain. |
|  | Are the desirable effects large relative to undesirable effects? | ☐ No  **■ Probably no**  ☐ Uncertain  ☐ Probably yes  ☐ Yes  ☐ Varies |  | Considering that there is no significant difference regarding mortality, morbidity, and admission to hospital. The desirable anticipated effects are probably not large relative to the undesirable effects. |
| **RESOURCE USE** | Are the resources required small? | ☐ No  ☐ Probably no  ☐ Uncertain  ☐ Probably yes  ■ **Yes**  ☐ Varies |  | The guideline panel decided that the resources necessary are probably small. |
|  | Is the incremental cost small relative to the net benefits? | ☐ No  ☐ Probably no  ☐ Uncertain  ■ **Probably yes**  ☐ Yes  ☐ Varies |  | There are costs involved. However, benefits are considered uncertain. |
| **EQUITY** | What would be the impact on health inequities? | ☐ Increased  ☐ Probably increased  ☐ Uncertain  ☐ Probably reduced  ☐ Reduced  ■ **Varies** |  | The panel expected that implementing this option has no effect on health inequities, given the structure of the Dutch healthcare system. However, in other countries this may vary depending on their healthcare system. |
| **ACCEPTABILITY** | Is the option acceptable to key stakeholders? | ☐ No  ☐ Probably no  ☐ Uncertain  ☐ Probably yes  ■ **Yes**  ☐ Varies |  | The guideline panel considered the option for an Hb threshold >4.3 mmol/L acceptable to the key stakeholders. |
| **FEASIBILITY** | Is the option feasible to implement? | ☐ No  ☐ Probably no  ☐ Uncertain  ☐ Probably yes  ■ **Yes**  ☐ Varies |  | The panel considered the option of a transfusion threshold >4.3 mol/l feasible to implement. |

| **Balance of consequences – Hb threshold >4.3 mmol/L relative to an Hb threshold <4.3 mmol/L** | | | | |
| --- | --- | --- | --- | --- |
| Undesirable consequences  *clearly outweigh*  desirable consequences in most settings  ☐ | Undesirable consequences *probably outweigh*  desirable consequences in most settings  ☐ | **The balance between  desirable and undesirable consequences  *is closely balanced***  ■ | Desirable consequences  *probably outweigh* undesirable consequences in most settings  ☐ | Desirable consequences  *clearly outweigh*  undesirable consequences in most settings  ☐ |

| **Type of recommendation – Hb threshold >4.3 mmol/L relative to an Hb threshold <4.3 mmol/L** | | | |
| --- | --- | --- | --- |
| We recommend against  offering this option  ☐ | **We suggest not offering**  **this option**  ■ | We suggest offering  this option  ☐ | We recommend offering  this option  ☐ |
| Recommendation (text) | We suggest a hemoglobin (Hb) threshold <4.3 mmol/L for RBC transfusion in children with cancer during sepsis (Weak recommendation). | | |
| Justification | Based on limited evidence there is a suggestion that there is no increased risk for mortality or morbidity with an Hb threshold for RBC transfusion <4.3 mmol/L in comparison to an Hb threshold >4.3 mmol/L in children and adults with sepsis (Lacroix, 2012; Holst, 2014). In addition, there are no studies reporting any significant potential benefit from an Hb threshold >4.3 mmol/L (Lacroix, 2012). In addition, this option of an Hb threshold <4.3 mmol/L is considered probably acceptable for all stakeholders. In addition, the expert panel considered that a higher Hb threshold might lead to more iron overload. Therefore, we suggest not offering the option of an Hb threshold >4.3 mmol/L. | | |
| Subgroup considerations | In hemodynamically unstable children with cancer during sepsis and evidence of oxygen deficiency without ongoing blood loss, it is recommended to consider an Hb threshold that ranges between <4.3 mmol/L and <6.2 mmol/L as part of a comprehensive approach to improve oxygen delivery for children with unstable non hemorrhagic shock and evidence of oxygen debt (based on Muszynski, 2018). | | |
| Implementation considerations | No implementation considerations were formulated. | | |
| Monitoring and evaluation | Not applicable. | | |
| Research priorities | See supplemental materials S9 "Gaps in research". | | |

**2.2 PROPHYLACTIC RED BLOOD CELL TRANSFUSION IN NEONATES WITH CANCER DURING SEPSIS**

**A. Primary search**No supporting materials.

**B. Additional search**

The full description of the additional guidelines and the AGREE II-scores are presented in Supplemental Materials S5.

**Table 1.** Included additional guidelines.

| **Research question 2A - The effect of prophylactic RBC transfusion in children with cancer who suffer from sepsis** | |
| --- | --- |
| Recommendations for children with sepsis | **Valentine (2018): Consensus Recommendations for RBC Transfusion Practice in Critically Ill Children From the Pediatric Critical Care Transfusion and Anemia Expertise Initiative. Pediatric Critical Care Medicine (Muszynski, 2018).**  *AGREE II assessment: Domain 1 = 89%, Domain 2 = 44%, Domain 3 = 79%, Domain 4 = 78%, Domain 5 = 63%, Domain 6 = 92%, Overall Guideline Assessment: Score 5.*  Recommendations for critically ill children with a non-hemorrhagic shock, such as a septic shock according to Valentine (2018):   - In **hemodynamically stable** critically ill children with a diagnosis of severe sepsis or septic shock, they **recommended not administering a RBC transfusion if the Hb concentration is ≥ 4.3 mmol/dL.** - Weak recommendation, Low quality pediatric evidence, 96% Agreement, (n=29), Median 8, IQR 8-9.   - Supporting arguments: The recommendation is based on the TRIPICU study and the adult study TRISS stating that an Hb threshold of 4.3 mmol/dL is safe (Lacroix, 2007; Holst, 2014) - In **hemodynamically unstable** critically ill children with a diagnosis of severe sepsis or septic shock and evidence of oxygen deficiency, they suggest **an Hb threshold between 4.3 mmol/dL and 6.2 mmol/dL**. Consensus panel expertise, 100% Agreement, Median 9, IQR 8-9   - Supporting arguments: The recommendation is based on consensus, due to lack of evidence. |

**C. Evidence to Decision Frameworks
Table 2:** Evidence to Decision Framework & Overall conclusions - >4.3 mmol/L versus <4.3 mmol/L.

| **Hemoglobin (Hb) threshold >4.3 mmol/L versus a hemoglobin (Hb) threshold of 4.3 mmol/L** | | | | |
| --- | --- | --- | --- | --- |
|  | **Criteria** | **Judgements** | **Research evidence** | **Additional considerations** |
| **PROBLEM** | Is the problem a priority? | ☐ No  ☐ Probably no  ☐ Uncertain  ☐ Probably yes  ■ **Yes** | RBC transfusions are one of the backbones in the supportive care management of children with oncologic diagnoses and those who are undergoing hematopoietic stem cell transplants (HSCT). Children with cancer may require RBC transfusions due to the underlying oncologic disease or bone marrow suppression as a result of the anti-cancer treatment. This guideline is the first guideline to be established specifically for children with cancer during sepsis. |  |
| **BENEFITS AND HARMS** | What is the overall certainty of this evidence? | ☐ No included studies  **■ Very low**  ☐ Low  ☐ Moderate  ☐ High | Summary of findings: Mortality  - Pediatric oncology: no studies included.  - Pediatric: Lacroix (2012) reported no significant differences regarding mortality when comparing an Hb threshold <4.3 mmol/L versus an Hb threshold <5.0 mmol/L, RR 3.45 (95% CI 0.74 - 16.02).  - Adult: Holst (2014) reported no significant differences regarding mortality when comparing an Hb threshold <4.3 mmol/L versus an Hb threshold <5.6 mmol/L, RR 0.96 (95% CI 0.83 - 1.10).  Morbidity  - Pediatric oncology: no included studies.  - Pediatric: Lacroix (2012) reported no significant differences regarding morbidity when comparing an Hb threshold <4.3 mmol/L versus an Hb threshold <5.0 mmol/L RR 0.99 (95% CI 0.49 - 1.97).  - Adult: Holst (2014) reported no significant differences regarding morbidity when comparing an Hb threshold <4.3 mmol/L versus an Hb threshold <5.6 mmol/L, RR 0.33 (95% CI 0.01 - 8.18).  Admission to hospital  - Pediatric oncology: no included studies.  - Pediatric: Lacroix (2012) reported no significant differences regarding admission to hospital when comparing an Hb threshold <4.3 mmol/L versus an Hb threshold <5.0 mmol/L.  - Adult: No pediatric oncology: no included studies. |  |
|  | Is there important uncertainty about how much people value the main outcomes? | ☐ Important uncertainty or variability  ☐ Possibly important uncertainty or variability  ☐ Probably no important uncertainty or variability  ■ **No important uncertainty or variability**  ☐ No known undesirable outcomes |  | The relative importance of all outcomes was unanimously determined. |
|  | Are the desirable anticipated effects large? | ☐ No  ■ **Probably no**  ☐ Uncertain  ☐ Probably yes  ☐ Yes  ☐ Varies |  | There is no significant difference regarding mortality and morbidity. Thus, the undesirable anticipated effects are probably not large. |
|  | Are the undesirable anticipated effects small? | ☐ No  ☐ Probably no  ■ **Uncertain**  ☐ Probably yes  ☐ Yes  ☐ Varies |  | Apart from admission to hospital, there are no other desirable anticipated effects included, thus uncertain. |
|  | Are the desirable effects large relative to undesirable effects? | ☐ No  **■ Probably no**  ☐ Uncertain  ☐ Probably yes  ☐ Yes  ☐ Varies |  | Considering that there is no significant difference regarding mortality, morbidity, and admission to hospital. The desirable anticipated effects are probably not large relative to the undesirable effects. |
| **RESOURCE USE** | Are the resources required small? | ☐ No  ☐ Probably no  ☐ Uncertain  ☐ Probably yes  ■ **Yes**  ☐ Varies |  | The guideline panel decided that the resources necessary are probably small. |
|  | Is the incremental cost small relative to the net benefits? | ☐ No  ☐ Probably no  ☐ Uncertain  ■ **Probably yes**  ☐ Yes  ☐ Varies |  | There are costs involved. However, benefits are considered uncertain. |
| **EQUITY** | What would be the impact on health inequities? | ☐ Increased  ☐ Probably increased  ☐ Uncertain  ☐ Probably reduced  ☐ Reduced  ■ **Varies** |  | The panel expected that implementing this option has no effect on health inequities, given the structure of the Dutch healthcare system. However, in other countries this may vary depending on their healthcare system. |
| **ACCEPTABILITY** | Is the option acceptable to key stakeholders? | ☐ No  ☐ Probably no  ☐ Uncertain  ☐ Probably yes  ■ **Yes**  ☐ Varies |  | The guideline panel considered the option for an Hb threshold >4.3 mmol/L acceptable to the key stakeholders. |
| **FEASIBILITY** | Is the option feasible to implement? | ☐ No  ☐ Probably no  ☐ Uncertain  ☐ Probably yes  ■ **Yes**  ☐ Varies |  | The panel considered the option of a transfusion threshold >4.3 mol/l feasible to implement. |

| **Balance of consequences – Hb threshold >4.3 mmol/L relative to an Hb threshold <4.3 mmol/L** | | | | |
| --- | --- | --- | --- | --- |
| Undesirable consequences  *clearly outweigh*  desirable consequences in most settings  ☐ | Undesirable consequences *probably outweigh*  desirable consequences in most settings  ☐ | **The balance between  desirable and undesirable consequences  *is closely balanced***  ■ | Desirable consequences  *probably outweigh* undesirable consequences in most settings  ☐ | Desirable consequences  *clearly outweigh*  undesirable consequences in most settings  ☐ |

| **Type of recommendation – Hb threshold >4.3 mmol/L relative to an Hb threshold <4.3 mmol/L** | | | |
| --- | --- | --- | --- |
| We recommend against  offering this option  ☐ | **We suggest not offering**  **this option**  ■ | We suggest offering  this option  ☐ | We recommend offering  this option  ☐ |
| Recommendation (text) | We suggest a hemoglobin (Hb) threshold <4.3 mmol/L for RBC transfusion in children with cancer during sepsis (Weak recommendation). | | |
| Justification | Based on limited evidence there is a suggestion that there is no increased risk for mortality or morbidity with an Hb threshold for RBC transfusion <4.3 mmol/L in comparison to an Hb threshold >4.3 mmol/L in children and adults with sepsis (Lacroix, 2012; Holst, 2014). In addition, there are no studies reporting any significant potential benefit from an Hb threshold >4.3 mmol/L (Lacroix, 2012). In addition, this option of an Hb threshold <4.3 mmol/L is considered probably acceptable for all stakeholders. In addition, the expert panel considered that a higher Hb threshold might lead to more iron overload. Therefore, we suggest not offering the option of an Hb threshold >4.3 mmol/L. | | |
| Subgroup considerations | In hemodynamically unstable children with cancer during sepsis and evidence of oxygen deficiency without ongoing blood loss, it is recommended to consider an Hb threshold that ranges between <4.3 mmol/L and <6.2 mmol/L as part of a comprehensive approach to improve oxygen delivery for children with unstable non hemorrhagic shock and evidence of oxygen debt (based on Muszynski, 2018). | | |
| Implementation considerations | No implementation considerations were formulated. | | |
| Monitoring and evaluation | Not applicable. | | |
| Research priorities | See supplemental materials S9 "Gaps in research". | | |

#

**3 PROPHYLACTIC RED BLOOD CELL TRANSFUSION - RADIOTHERAPY**

**3.1 PROPHYLACTIC RED BLOOD CELL TRANSFUSION IN CHILDREN WHO UNDERGO RADIOTHERAPY
A. Primary search**No supporting materials.

**B. Additional search**

The full description of the additional guidelines and the AGREE II-scores are presented in Supplemental Materials S5.

**Table 1.** Included additional guidelines.

| **Research question 3A - The effect of prophylactic RBC transfusion in children with cancer who undergo radiotherapy** | |
| --- | --- |
| Recommendations for adults who undergo radiotherapy | **National Blood Authority (2012): Patient Blood Management Guidelines module 3.**  *AGREE II assessment: Domain 1 = 89%, Domain 2 = 44%, Domain 3 = 67%, Domain 4 = 89%, Domain 5 = 29%, Domain 6 = 58%, Overall Guideline Assessment: Score 4.*  Recommendations according to the Patient Blood Management Guidelines National Blood Authority (2012):   - The **same hemoglobin (Hb) thresholds as other patients with cancer**.   - Supporting arguments: Based on a review stating that the correction of anemia by RBC transfusions led to adverse effects (Varlotto & Stevenson, 2005). |

**3.2 PROPHYLACTIC RED BLOOD CELL TRANSFUSION IN NEONATES WHO UNDERGO RADIOTHERAPY
A. Primary search**No supporting materials.

**B. Additional search**

The full description of the additional guidelines and the AGREE II-scores are presented in Supplemental Materials S5.

**Table 1.** Included additional guidelines.

| **Research question 3B - The effect of prophylactic RBC transfusion in neonates with cancer who undergo radiotherapy** | |
| --- | --- |
| Recommendations for adults who undergo radiotherapy | **National Blood Authority (2012): Patient Blood Management Guidelines module 3.**  *AGREE II assessment: Domain 1 = 89%, Domain 2 = 44%, Domain 3 = 67%, Domain 4 = 89%, Domain 5 = 29%, Domain 6 = 58%, Overall Guideline Assessment: Score 4.*  Recommendations according to the Patient Blood Management Guidelines National Blood Authority (2012):   - The **same Hb thresholds as other patients with cancer**.   - Supporting arguments: Based on a review stating that the correction of anemia by RBC transfusions led to adverse effects (Varlotto & Stevenson, 2005). |

**4. PROPHYLACTIC RED BLOOD CELL TRANSFUSION - CARDIAC AND PULMONARY COMORBIDITIES**

**4.1 PROPHYLACTIC RED BLOOD CELL TRANSFUSION IN CHILDREN WITH CANCER WITH CARDIAC AND/OR PULMONARY COMORBIDITIES**

**A. Primary search**No supporting materials.

**B. Additional search**

The full description of the additional guidelines and the AGREE II-scores are presented in Supplemental Materials S5.

**Table 1.** Included additional guidelines.

| **Research question 4A - The effect of prophylactic RBC transfusion in children with cancer with cardiac and pulmonary comorbidity** | |
| --- | --- |
| Recommendations for children with acute respiratory failure | **Valentine (2018): Consensus Recommendations for RBC Transfusion Practice in Critically Ill Children From the Pediatric Critical Care Transfusion and Anemia Expertise Initiative. Pediatric Critical Care Medicine (Demaret, 2018).**  *AGREE II assessment: Domain 1 = 89%, Domain 2 = 44%, Domain 3 = 79%, Domain 4 = 78%, Domain 5 = 63%, Domain 6 = 92%, Overall Guideline Assessment: Score 5.*  Recommendations for critically ill children with acute respiratory failure according to Valentine (2018):   - In case of a critically ill **child with respiratory failure an Hb threshold of 3.1 mmol/L is recommended**. Strong recommendation, Low quality pediatric evidence (1C), 100% Agreement, (n=35), Median 9 IQR 8-9.   - Supporting arguments: The recommendation is based on several studies in children and adults stating that an Hb <3.1 mmol/dL is associated with adverse patient outcomes (Marsh, 1995; English, 2002; Lackritz, 1992; Viele & Weiskopf, 1994; Carson, 2002; Shander, 2014). - In critically ill children with **respiratory failure without severe acute hypoxemia, a chronic cyanotic condition or hemolytic anemia, and with a stable hemodynamic situation a RBC transfusion >4.3 mmol/L is not recommended**. Strong recommendation, Moderate quality pediatric evidence (1B), 100% Agreement, (n=29), Median 8.5, IQR 8-9.   - Supporting arguments: This recommendation is based on the TRIPICU study stating that an Hb >4.3 mmol/dL is safe (Lacroix, 2007). - In critically ill children with **respiratory failure and severe hypoxemia a recommendation could not be made**. Consensus panel expertise, 97% Agreement, (n=29), Median 8, IQR 8-9.   - Supporting arguments: The recommendation is based on consensus, due to lack of evidence. - There was **not enough evidence to create a recommendation regarding RBC transfusion thresholds between 3.1 - 4.3 mmol/L**, but the clinical judgements should be considered. Consensus panel expertise, 97% Agreement, (n=35), Median 9 IQR 8-9.   - Supporting arguments: The recommendation is based on consensus, due to lack of evidence. |
| Recommendations for children with acquired and congenital heart disease | **Valentine (2018): Consensus Recommendations for RBC Transfusion Practice in Critically Ill Children From the Pediatric Critical Care Transfusion and Anemia Expertise Initiative. Pediatric Critical Care Medicine (Cholette, 2018).**  *AGREE II assessment: Domain 1 = 89%, Domain 2 = 44%, Domain 3 = 79%, Domain 4 = 78%, Domain 5 = 63%, Domain 6 = 92%, Overall Guideline Assessment: Score 5.*  Recommendations for critically ill children with acquired and congenital heart disease according to Valentine (2018):   - Children with **right or left ventricular myocardial dysfunction, acquired or congenital: there is insufficient evidence to support RBC transfusion thresholds. There is no evidence that an Hb level >6.2 mmol/dL is beneficial**. Consensus panel expertise, 83% Agreement, (n=30), Median 8 IQR 7.25-8.75.   - Supporting arguments: The recommendation is based on consensus, due to lack of evidence. - Children with a **structurally normal heart and idiopathic or acquired pulmonary hypertension (mean pulmonary arterial pressure >25 mmHg with normal pulmonary capillary wedge pressure): There is insufficient evidence to support RBC transfusion thresholds. There is no evidence that an Hb level >6.2 mmol/dL is beneficial**. Consensus panel expertise, 97% Agreement, (n=35), Median 9, IQR 8-9.   - Supporting arguments: The recommendation is based on consensus, due to lack of evidence. - In a hemodynamically stable critically ill child with **uncorrected congenital heart disease an Hb threshold between 4.3 mmol/dL and 5.6 mmol/dL is advised, depending on the degree of cardiopulmonary reserve**. Weak recommendation, Low quality pediatric evidence (2C), 81% Agreement, (n=35), Median 8, IQR 7-8.   - Supporting arguments: This recommendation is based on the TRIPICU study stating that an Hb >4.3 mmol/dL is safe (Lacroix, 2007). However, there is no evidence that transfusion to the Hb >5.6 mmol/dL is beneficial and might be of some risk. |
|  | **British Committee for Standards in Haematology (2016): Guidelines on transfusion for fetuses, neonates, and older children.**  *AGREE II assessment: Domain 1 = 83%, Domain 2 = 55%, Domain 3 = 54%, Domain 4 = 83%, Domain 5 = 29%, Domain 6 = 50%, Overall Guideline Assessment: Score 4.*  Recommendations according to New (2016):   - **An Hb threshold <4.3 mmol/L is advised in stable children with non-cyanotic heart disease**. 2B recommendation.   - Supporting arguments: Based on the TRIPICU study and others (Lacroix, 2007; Lacroix, 2012; Carson, 2012; Retter, 2013; Hébert, 1999; Hajjar, 2010; Carson, 2011; NICE, 2015) - There is **insufficient evidence to make a recommendation for children with cyanotic heart disease**. 2C recommendation. - There is **insufﬁcient evidence to make recommendations for pre-transfusion Hb thresholds in pediatric hematology/oncology patients and those undergoing stem cell transplantation.** 2C recommendation. |

**C. Evidence to Decision Frameworks
Table 2.** Evidence to Decision Framework & Overall conclusions - <4.3 mmol/L versus >4.3 mmol/L.

| **Hb threshold >4.3 mmol/L (intervention) versus an Hb threshold <4.3 mmol/L (control)** | | | | |
| --- | --- | --- | --- | --- |
|  | **Criteria** | **Judgements** | **Research evidence** | **Additional considerations** |
| **PROBLEM** | Is the problem a priority? | ☐ No  ☐ Probably no  ☐ Uncertain  ☐ Probably yes  ■ **Yes** | RBC transfusions are one of the backbones in the supportive care management of children with oncologic diagnoses and those who are undergoing hematopoietic stem cell transplants (HSCT). Children with cancer may require RBC transfusions due to the underlying oncologic disease or bone marrow suppression as a result of the anti-cancer treatment. This guideline is the first guideline to be established specifically for pediatric oncology. |  |
| **BENEFITS AND HARMS** | What is the overall certainty of this evidence? | ☐ No included studies  **■ Very low**  ☐ Low  ☐ Moderate  ☐ High | Summary of findings:  1. Mortality - Pediatric oncology: no included studies.  - Pediatric: Willems (2010) reported no significant difference regarding mortality when comparing an Hb threshold <4.3 mmol/L versus <5.0 mmol/L, RR 0.98 (95% CI 0.14 - 6.77). *Evidence cited in existing guidelines.*  - Adult: The results from Carson (2002) were not estimable considering that no one died when comparing an Hb threshold <4.3 mmol/L versus <5.0 mmol/L. *Evidence cited in existing guidelines.*  5. Morbidity  - Pediatric oncology: no included studies.  - Pediatric: Willems (2010), and Lacroix (2012) both reported no significant differences regarding morbidity when comparing an Hb threshold <4.3 mmol/L with <5.0 mmol/L, RR 1.97 (95% CI 0.62 - 6.20, RR 0.99 (95% CI 0.64 - 1.54) respectively. *Evidence cited in existing guidelines.* - Adult: no included studies.  7. Admission to hospital  - Pediatric oncology: no included studies.  - Pediatric: Willems (2010), and Lacroix (2012) reported no significant differences regarding admission to hospital when comparing an Hb threshold <4.3 mmol/L with <5.0 mmol/L, MD -0.40 (95% CI -2.42 - 1.62), and MD 0.10 (95% CI -.78 - 0.98) respectively. *Evidence cited in existing guidelines.* |  |
|  | Are the desirable anticipated effects large? | ☐ No  ■ **Probably no**  ☐ Uncertain  ☐ Probably yes  ☐ Yes  ☐ Varies |  | There is no significant difference regarding mortality and morbidity. Thus the desirable effects are probably small. |
|  | Are the undesirable anticipated effects small? | ☐ No  ☐ Probably no  **■ Uncertain**  ☐ Probably yes  ☐ Yes  ☐ Varies |  | Apart from admission to hospital, no other undesirable effects are included, thus uncertain. |
|  | Are the desirable effects large relative to undesirable effects? | ☐ No  **■ Probably no**  ☐ Uncertain  ☐ Probably yes  ☐ Yes  ☐ Varies |  | Considering that there is no significant difference regarding mortality or morbidity and there is no significant difference regarding admission to hospital, the desirable effects are probably not large relative to the undesirable effects. |
| **RESOURCE USE** | Are the resources required small? | ☐ No  ■ **Probably no**  ☐ Uncertain  ☐ Probably yes  ☐ Yes  ☐ Varies |  | The guideline panel decided that the resources necessary are probably not small, e.g., hospital admission costs. |
|  | Is the incremental cost small relative to the net benefits? | ☐ No  ■ **Probably no**  ☐ Uncertain  ☐ Probably yes  ☐ Yes  ☐ Varies |  | There are costs involved (e.g., hospital admission costs). However, benefits are considered probably not large |
| **EQUITY** | What would be the impact on health inequities? | ☐ Increased  ☐ Probably increased  ☐ Uncertain  ☐ Probably reduced  ☐ Reduced  ■ **Varies** |  | The panel expected that implementing this option has no effect on health inequities, given the structure of the Dutch healthcare system. However, in other countries this may vary depending on their healthcare system. |
| **ACCEPTABILITY** | Is the option acceptable to key stakeholders? | ☐ No  ☐ Probably no  ☐ Uncertain  ■ **Probably yes**  ☐ Yes  ☐ Varies |  | The panel considered the option of a transfusion threshold <4.3 mmol/L acceptable for the key stakeholders, e.g., doctors and parents. However, it is unknown if some parties might find it not acceptable, thus probably yes. |
| **FEASIBILITY** | Is the option feasible to implement? | ☐ No  ☐ Probably no  ☐ Uncertain  ☐ Probably yes  ■ **Yes**  ☐ Varies |  | The panel considered the option of a transfusion threshold <4.3 mol/l feasible to implement. |

| **Balance of consequences – Hb threshold >4.3 mmol/L relative to an Hb threshold <4.3 mmol/L** | | | | |
| --- | --- | --- | --- | --- |
| Undesirable consequences  *clearly outweigh*  desirable consequences in most settings  ☐ | **Undesirable consequences *probably outweigh*  desirable consequences in most settings**  ■ | The balance between  desirable and undesirable consequences  *is closely balanced*  ☐ | Desirable consequences  *probably outweigh* undesirable consequences in most settings  ☐ | Desirable consequences  *clearly outweigh*  undesirable consequences in most settings  ☐ |

| **Type of recommendation – Hb threshold >4.3 mmol/L relative to an Hb threshold <4.3 mmol/L** | | | |
| --- | --- | --- | --- |
| We recommend against  offering this option  ☐ | **We suggest not offering**  **this option**  ■ | We suggest offering  this option  ☐ | We recommend offering  this option  ☐ |
| Recommendation (text) | We suggest a hemoglobin (Hb) threshold <4.3 mmol/L for RBC transfusion in children with cancer and cardiac and pulmonary comorbidities (weak recommendation). | | |
| Justification | Regarding the comparison of an Hb threshold <4.3 mmol/L to an Hb threshold >4.3 mmol/L, 3 pediatric non-cancer studies and 1 adult non-cancer study were identified. Based on the evidence there is a suggestion that there is no increased risk for mortality, morbidity, and hospital admission with an Hb threshold for RBC transfusion <4.3 mmol/L in comparison to an Hb >4.3 mmol/L in children and adults with cardiac and pulmonary comorbidities (Lacroix, 2012; Carson, 2002; Willems, 2010). In addition, this option is considered probably acceptable for all stakeholders. Studies that included higher restrictive Hb thresholds (such as <5.0 mmol/L and 5.6 mmol/L) also did not report significant outcomes regarding mortality, morbidity, quality of life, and admission to hospital (Hajjar, 2010; Carson, 2011; Cholette, 2011). Therefore, the guideline panel decided to suggest an Hb threshold <4.3 mmol/L in clinically stable children with cancer and cardiac and pulmonary comorbidities. | | |
| Subgroup considerations | It is reasonable to consider transfusion based on clinical judgment in these children. In case of a hemodynamically unstable child with cancer and pulmonary and/or cardiac comorbidities (e.g., use of inotropes, elevated lactate) a higher Hb threshold can be considered. | | |
| Implementation considerations | No implementation considerations were formulated. | | |
| Monitoring and evaluation | Not applicable | | |
| Research priorities | See supplemental materials S9 "Gaps in research". | | |

**4.2 PROPHYLACTIC RED BLOOD CELL TRANSFUSION IN NEONATES WITH CANCER WITH CARDIAC AND/OR PULMONARY COMORBIDITIES**

**A. Primary search**No supporting materials.

**B. Additional search**

The full description of the additional guidelines and the AGREE II-scores are presented in Supplemental Materials S5.

**Table 1.** Included additional guidelines.

| **Research question 4B - The effect of prophylactic RBC transfusion in neonates with cancer with cardiac and pulmonary comorbidities** | |
| --- | --- |
| Recommendations for neonates with cardiac and pulmonary comorbidities | **Federation of Medical Specialists (2019): Blood transfusion policy.**  *AGREE II assessment: Domain 1 = 94%, Domain 2 = 100%, Domain 3 = 71%, Domain 4 = 89%, Domain 5 = 13%, Domain 6 = 100%, Overall Guideline Assessment: Score 6.*  Recommendations according to the Federation of Medical Specialists (2019):   - That very low birth weight infants (birth weight <1500 grams) should receive restrictive RBC transfusions in infants with cardiac and or pulmonary comorbidity and in absence of studies about full term neonates and late preterm infants (gestational age>32 weeks), these recommendations are also applied to these groups:   - Maintain **an Hb >7.5 mmol/L in neonates <1 week old**.   - Maintain **an Hb >6.5 mmol/L in neonates between 2 and 3 weeks old**.   - Maintain **an Hb >5.5 mmol/L in neonates >3 weeks old**.     - Supporting arguments: Based on 4 studies and 1 Cochrane-analysis (Connelly, 1999; Bell, 2005; Kirpalani, 2006; Chen, 2009; Whyte & Kirpalani, 2011). |
|  | **Valentine (2018): Consensus Recommendations for RBC Transfusion Practice in Critically Ill Children From the Pediatric Critical Care Transfusion and Anemia Expertise Initiative. Pediatric Critical Care Medicine.**  *AGREE II assessment: Domain 1 = 89%, Domain 2 = 44%, Domain 3 = 79%, Domain 4 = 78%, Domain 5 = 63%, Domain 6 = 92%, Overall Guideline Assessment: Score 5.*  Recommendations for critically ill neonates with acquired and congenital heart disease according to Valentine (2018):   - In a **hemodynamically stable** critically ill infant with uncorrected congenital heart disease **an Hb threshold between 4.3 mmol/dL and 5.6 mmol/dL is advised**, depending on the degree of cardiopulmonary reserve. Weak recommendation, low quality pediatric evidence (2C), 81% Agreement, (n=35), Median 8, IQR 7-8.   - Supporting arguments: This recommendation is based on the TRIPICU study stating that an Hb >4.3 mmol/dL is safe (Lacroix, 2007). However, there is no evidence that transfusion to the Hb >5.6 mmol/dL is beneficial and might be of some risk. |
|  | **JPAC (2013): Transfusion Handbook**  *AGREE II assessment: Domain 1 = 94%, Domain 2 = 56%, Domain 3 = 35%, Domain 4 = 56%, Domain 5 = 58%, Domain 6 = 25%, Overall Guideline Assessment: Score 4.*  The Transfusion Handbook from the JPAC (2013) advises a neonatal top-up transfusion threshold:   - In case of ventilated neonates:   - Maintain **an Hb >7.5 mmol/L in neonates <24 hours old**.   - Maintain **an Hb >7.5 mmol/L in neonates <1 week old**.   - Maintain **an Hb >6.2 mmol/L in neonates between 2-3 weeks old**.   - Maintain **an Hb >6.2 mmol/L in neonates >4 weeks old**.     - Supporting arguments: Based on the British Committee for Standards in Haematology Transfusion Guidelines for Neonates and Older Children (New, 2016). - In case of neonates on oxygen/CPAP:   - Maintain **an Hb >7.5 mmol/L in neonates <24 hours old**.   - Maintain **an Hb >6.2 mmol/L in neonates <1 week old**.   - Maintain **an Hb >5.9 mmol/L in neonates between 2-3 weeks old**.   - Maintain **an Hb >5.3 mmol/L in neonates >4 weeks old**.     - Supporting arguments: Based on the British Committee for Standards in Haematology Transfusion Guidelines for Neonates and Older Children (New, 2016). |
|  | **British Committee for Standards in Haematology (2016): Guidelines on transfusion for fetuses, neonates and older children.**  *AGREE II assessment: Domain 1 = 83%, Domain 2 = 55%, Domain 3 = 54%, Domain 4 = 83%, Domain 5 = 29%, Domain 6 = 50%, Overall Guideline Assessment: Score 4.*  Recommendations according to New (2016):   - In case of ventilated neonates:   - Maintain **an Hb >7.5 mmol/L in neonates <24 hours old**.   - Maintain **an Hb >7.5 mmol/L in neonates <1 week old**.   - Maintain **an Hb >6.2 mmol/L in neonates between 2-3 weeks old**.   - Maintain **an Hb >6.2 mmol/L in neonates >4 weeks old**.     - Supporting arguments: Based on studies (Whyte & Kirpalani, 2006; Chen, 2009, Bell, 2005; Whyte & Kirpalani, 2011; Venkatesh, 2012; Brooks 1999; Mukhopadhyay, 2004; Ransome, 1989) - In case of neonates on oxygen/CPAP:   - Maintain **an Hb >7.5 mmol/L in neonates <24 hours old**.   - Maintain **an Hb >6.2 mmol/L in neonates <1 week old**.   - Maintain **an Hb >5.9 mmol/L in neonates between 2-3 weeks old**.   - Maintain **an Hb >5.3 mmol/L in neonates >4 weeks old**.     - Supporting arguments: Based on studies (Whyte & Kirpalani, 2006; Chen, 2009, Bell, 2005; Whyte & Kirpalani, 2011; Venkatesh, 2012). |

**5. PROPHYLACTIC RED BLOOD CELL TRANSFUSION - HYPERLEUKOCYTOSIS**

**5.1 PROPHYLACTIC RED BLOOD CELL TRANSFUSION IN CHILDREN AND NEONATES WITH CANCER DURING HYPERLEUKOCYTOSIS**

As no studies were included, there are no supporting materials attached.

**6. IRRADIATED RED BLOOD CELL TRANSFUSIONS
6.1 IRRADIATED RED BLOOD CELL TRANSFUSIONS IN CHILDREN AND NEONATES WITH CANCER
A. Primary search**No supporting materials.

**B. Additional search**

The full description of the additional guidelines and the AGREE II-scores are presented in Supplemental Materials S5.

**Table 1.** Included additional guidelines.

| **Research question 6A/B - The effect of irradiated RBC products in children and neonates with cancer** | |
| --- | --- |
| Recommendations for children | **Federation of Medical Specialists (2019): Blood transfusion policy.**  *AGREE II assessment: Domain 1 = 94%, Domain 2 = 100%, Domain 3 = 71%, Domain 4 = 89%, Domain 5 = 13%, Domain 6 = 100%, Overall Guideline Assessment: Score 6.*  Recommendations for irradiated RBC products according to Dutch Association of Medical Specialists (FMS, 2019):   1. In case of HLA related products and donors: 2. Transfusion between 1st to 3rd degree relatives of cell-containing blood products; 3. HLA-compatible plated concentrates.  - In case of granulocyte transfusions - Depending on the patient's immune status:  1. During intrauterine transfusions until 6 months after the due date; 2. Children with congenital combined immune deficiencies (e.g. SCID); 3. Acquired immune deficiencies such as:    - Allogeneic stem cell transplantations up to 1 year after transplantation;    - Autologous stem cell transplantations up to 6 months after transplantation;    - After application of donor lymphocyte infusion (DLI) or infusion of cytotoxic T lymphocytes (CTL) up to 1 year after transfusion.  - In case of patients with prolonged T-cell depletion after medication:  1. Fludarabine or other T-cell depleting therapy or indicated by the pharmacotherapeutic compass (up to 6 months after discontinuation of the therapy); 2. Medications that, in combination with the disease, cause long-term T-cell depletions, such as anti-CD52 treatments in hematological diseases and ATG treatment in aplastic anemia from the initiation to 6 months after completion of the treatment. |

**7. LOW OR HIGH-VOLUME RED BLOOD CELL TRANSFUSIONS
7.1 LOW OR HIGH-VOLUME RED BLOOD CELL TRANSFUSIONS IN CHILDREN WITH CANCER**

**A. Primary search**No supporting materials.

**B. Additional search**

No supporting materials.

**C. Evidence to Decision Frameworks
Table 1.** Evidence to Decision Framework & Overall conclusions - <20 ml/kg versus >20 ml/kg.

| **Prophylactic RBC transfusion volume 20 ml/kg versus >20 ml/kg** | | | | |
| --- | --- | --- | --- | --- |
|  | **Criteria** | **Judgements** | **Research evidence** | **Additional considerations** |
| **PROBLEM** | Is the problem a priority? | ☐ No  ☐ Probably no  ☐ Uncertain  ☐ Probably yes  ■ **Yes** | RBC transfusions are one of the backbones in the supportive care management of children with oncologic diagnoses and those who are undergoing hematopoietic stem cell transplants (HSCT). Children with cancer may require RBC transfusions due to the underlying oncologic disease or bone marrow suppression as a result of the anti-cancer treatment. This guideline is the first guideline to be established specifically for RBC transfusion volumes in children with cancer. |  |
| **BENEFITS AND HARMS** | What is the overall certainty of this evidence? | ☐ No included studies  **■ Very low**  ☐ Low  ☐ Moderate  ☐ High | Summary of findings:  Mortality - Pediatric oncology: no included studies.  - Pediatric: Olupot-Olupot (2014) reported no significant differences regarding mortality when comparing a volume of 20 ml/kg with 30 ml/kg, RR 5.71 (95% CI 0.70 - 46.34).  - Adult: no included studies.  Morbidity  - Pediatric oncology: no included studies.  - Pediatric: Olupot-Olupot (2014) reported no significant differences regarding morbidity when comparing a volume of 20 ml/kg with 30 ml/kg, RR 2.85 (95% CI 0.59 - 13.72).  - Adult: no included studies.  Costs  - Pediatric oncology: no included studies.  - Pediatric: Olupot-Olupot (2014) reported no significant differences regarding costs when comparing a volume of 20 ml/kg with 30 ml/kg, RR 2.85 (95% CI 0.96 - 8.47).  - Adult: no included studies. |  |
|  | Is there important uncertainty about how much people value the main outcomes? | ☐ Important uncertainty or variability  ☐ Possibly important uncertainty or variability  ☐ Probably no important uncertainty or variability  ■ **No important uncertainty or variability**  ☐ No known undesirable outcomes |  | The relative importance of all outcomes was unanimously determined. |
|  | Are the desirable anticipated effects large? | ☐ No  ☐ Probably no  ■ **Uncertain**  ☐ Probably yes  ☐ Yes  ☐ Varies |  | The desirable effects are unknown e.g., quality of life, hospital admission, costs. |
|  | Are the undesirable anticipated effects small? | ☐ No  ☐ Probably no  ☐ Uncertain  ■ **Probably yes**  ☐ Yes  ☐ Varies |  | There is no significant difference regarding mortality and morbidity. Thus the undesirable effects are probably small. |
|  | Are the desirable effects large relative to undesirable effects? | ☐ No  ☐ Probably no  ■ **Uncertain**  ☐ Probably yes  ☐ Yes  ☐ Varies |  | Considering that there are probably no large, anticipated effects and the undesirable effects are uncertain. Thus, the desirable effects are uncertain relative to the undesirable effects. |
| **RESOURCE USE** | Are the resources required small? | ☐ No  ☐ Probably no  ☐ Uncertain  ■ **Probably yes**  ☐ Yes  ☐ Varies |  | The guideline panel decided that the resources necessary are probably small. |
|  | Is the incremental cost small relative to the net benefits? | ☐ No  ☐ Probably no  ■ **Uncertain**  ☐ Probably yes  ☐ Yes  ☐ Varies |  | There are costs involved (e.g., hospital admission costs). However, benefits are considered uncertain. |
| **EQUITY** | What would be the impact on health inequities? | ☐ Increased  ☐ Probably increased  ☐ Uncertain  ☐ Probably reduced  ☐ Reduced  ■ **Varies** |  | The panel expected that implementing this option has no effect on health inequities, given the structure of the Dutch healthcare system. However, considering the different healthcare structures in the world the impact may vary per country. |
| **ACCEPTABILITY** | Is the option acceptable to key stakeholders? | ☐ No  ☐ Probably no  ☐ Uncertain  ■ **Probably yes**  ☐ Yes  ☐ Varies |  | The panel considered the option of a transfusion volume of 20 ml/kg probably acceptable for the key stakeholders, e.g. doctors and parents. |
| **FEASIBILITY** | Is the option feasible to implement? | ☐ No  ☐ Probably no  ☐ Uncertain  ☐ Probably yes  ■ **Yes**  ☐ Varies |  | The panel considered the option of a transfusion volume of 20 ml/kg feasible to implement. |

| **Balance of consequences – Prophylactic RBC transfusion volume 20 ml/kg versus >20 ml/kg** | | | | |
| --- | --- | --- | --- | --- |
| Undesirable consequences  *clearly outweigh*  desirable consequences in most settings  ☐ | Undesirable consequences *probably outweigh*  desirable consequences in most settings  ☐ | **The balance between  desirable and undesirable consequences  *is closely balanced***  ■ | Desirable consequences  *probably outweigh* undesirable consequences in most settings  ☐ | Desirable consequences  *clearly outweigh*  undesirable consequences in most settings  ☐ |

| **Type of recommendation – Prophylactic RBC transfusion volume 20 ml/kg versus >20 ml/kg** | | | |
| --- | --- | --- | --- |
| We recommend against  offering this option  ☐ | **We suggest not offering**  **this option**  ■ | We suggest offering  this option  ☐ | We recommend offering  this option  ☐ |
| Recommendation (text) | We suggest not offering a transfusion volume of 20 ml/kg or higher in neonates with cancer (Weak recommendation). | | |
| Justification | Based on limited evidence there is a suggestion that a RBC transfusion volume of 20 ml/kg in comparison to a RBC transfusion volume of >20 ml/kg does not lead to more mortality or morbidity (Olupot-Olupot, 2014). Moreover, there are no studies included that report any potential benefit from a RBC transfusion volume >20 ml/kg, apart from no significant difference regarding costs (Olupot-Olupot, 2014). In addition, the expert panel decided that a lower transfusion volume leads to less exposure and this option is considered probably acceptable for all stakeholders. Therefore we suggest not offering this option. | | |
| Subgroup considerations | No subgroup considerations were formulated. | | |
| Implementation considerations | No implementation considerations were formulated. | | |
| Monitoring and evaluation | Not applicable | | |
| Research priorities | See supplemental materials S9 "Gaps in research". | | |

**7.2 LOW OR HIGH-VOLUME RED BLOOD CELL TRANSFUSIONS IN NEONATES WITH CANCER
A. Primary search**No supporting materials.

**B. Additional search**

The full description of the additional guidelines and the AGREE II-scores are presented in Supplemental Materials S5.

**Table 1.** Included additional guidelines.

| **Research question 7B - The effect of low-volume prophylactic RBC transfusion compared to high-volume RBC transfusion in neonates with cancer** | |
| --- | --- |
| Recommendation for neonates | **Federation of Medical Specialists (2019): Blood transfusion policy.**  *AGREE II assessment: Domain 1 = 94%, Domain 2 = 100%, Domain 3 = 71%, Domain 4 = 89%, Domain 5 = 13%, Domain 6 = 100%, Overall Guideline Assessment: Score 6.*  Recommendations according to the Federation of Medical Specialists (2019):   - In case of (extremely) premature neonates the blood transfusions must be given “top-up” considering the “anemia of the premature”. The transfusion volume differs between 10 to 20 ml/kg. There is little literature regarding the optimal transfusion volume:   - Supporting arguments: 2 studies have shown that a high-volume transfusion does not have negative effects on the patient. However in another studie it has been shown that a high-volume (20 ml/kg) transfusion does not have any beneficial effects for the patients as well, but a low-volume (15 ml/kg) reduces the total transfusion volume and thus donor exposition. Conclusion: **Transfuse with 15 ml/kg** (Wong, 2005; Paul, 2002; Khodabux, 2009). |
|  | **British Committee for Standards in Haematology (2016): Guidelines on transfusion for fetuses, neonates and older children.**  *AGREE II assessment: Domain 1 = 83%, Domain 2 = 55%, Domain 3 = 54%, Domain 4 = 83%, Domain 5 = 29%, Domain 6 = 50%, Overall Guideline Assessment: Score 4.*  Recommendations according to New (2016):   - **A volume of 15 ml/kg**.   Supporting arguments: Based on consensus |

**C. Evidence to Decision Frameworks
Table 2.** Evidence to Decision Framework & Overall conclusions - <10 ml/kg versus >10 ml/kg.

| **Prophylactic RBC transfusion volume 10 ml/kg versus >10 ml/kg** | | | | |
| --- | --- | --- | --- | --- |
|  | **Criteria** | **Judgements** | **Research evidence** | **Additional considerations** |
| **PROBLEM** | Is the problem a priority? | ☐ No  ☐ Probably no  ☐ Uncertain  ☐ Probably yes  ■ **Yes** | RBC transfusions are one of the backbones in the supportive care management of children with oncologic diagnoses and those who are undergoing hematopoietic stem cell transplants (HSCT). Children with cancer may require RBC transfusions due to the underlying oncologic disease or bone marrow suppression as a result of the anti-cancer treatment. This guideline is the first guideline to be established specifically for RBC transfusion volumes in children with cancer. |  |
| **BENEFITS AND HARMS** | What is the overall certainty of this evidence? | ☐ No included studies  **■ Very low**  ☐ Low  ☐ Moderate  ☐ High | Summary of findings:  Morbidity  - Pediatric oncology: no included studies.  - Pediatric: Paul (2002) reported no significant differences regarding morbidity when comparing a volume of 10 ml/kg with 20 ml/kg.  - Adults: no included studies. |  |
|  | Is there important uncertainty about how much people value the main outcomes? | ☐ Important uncertainty or variability  ☐ Possibly important uncertainty or variability  ☐ Probably no important uncertainty or variability  ■ **No important uncertainty or variability**  ☐ No known undesirable outcomes |  | The relative importance of all outcomes was unanimously determined. |
|  | Are the desirable anticipated effects large? | ☐ No  ☐ Probably no  ■ **Uncertain**  ☐ Probably yes  ☐ Yes  ☐ Varies |  | The desirable effects are unknown e.g., quality of life, hospital admission, costs. |
|  | Are the undesirable anticipated effects small? | ☐ No  ☐ Probably no  ☐ Uncertain  ■ **Probably yes**  ☐ Yes  ☐ Varies |  | There is no significant difference regarding morbidity. Thus, there are probably small undesirable anticipated effects. |
|  | Are the desirable effects large relative to undesirable effects? | ☐ No  ☐ Probably no  ■ **Uncertain**  ☐ Probably yes  ☐ Yes  ☐ Varies |  | Considering that there are probably no large, anticipated effects and the undesirable effects are uncertain. Thus, the desirable effects are uncertain relative to the undesirable effects. |
| **RESOURCE USE** | Are the resources required small? | ☐ No  ☐ Probably no  ☐ Uncertain  ■ **Probably yes**  ☐ Yes  ☐ Varies |  | The guideline panel decided that the resources necessary are probably small. |
|  | Is the incremental cost small relative to the net benefits? | ☐ No  ☐ Probably no  ■ **Uncertain**  ☐ Probably yes  ☐ Yes  ☐ Varies |  | There are costs involved (e.g., hospital admission costs). However, benefits are considered uncertain. |
| **EQUITY** | What would be the impact on health inequities? | ☐ Increased  ☐ Probably increased  ☐ Uncertain  ☐ Probably reduced  ☐ Reduced  ■ **Varies** |  | The panel expected that implementing this option has no effect on health inequities, given the structure of the Dutch healthcare system. However, in other countries this may vary depending on their healthcare system. |
| **ACCEPTABILITY** | Is the option acceptable to key stakeholders? | ☐ No  ☐ Probably no  ☐ Uncertain  ■ **Probably yes**  ☐ Yes  ☐ Varies |  | The panel considered the option of a transfusion volume of 10 ml/kg probably acceptable for the key stakeholders, e.g., doctors and parents. |
| **FEASIBILITY** | Is the option feasible to implement? | ☐ No  ☐ Probably no  ☐ Uncertain  ☐ Probably yes  ■ **Yes**  ☐ Varies |  | The panel considered the option of a transfusion volume of 10 ml/kg feasible to implement. |

| **Balance of consequences – Prophylactic RBC transfusion volume 10 ml/kg versus >10 ml/kg** | | | | |
| --- | --- | --- | --- | --- |
| Undesirable consequences  *clearly outweigh*  desirable consequences in most settings  ☐ | Undesirable consequences *probably outweigh*  desirable consequences in most settings  ☐ | **The balance between  desirable and undesirable consequences  *is uncertain***  ■ | Desirable consequences  *probably outweigh* undesirable consequences in most settings  ☐ | Desirable consequences  *clearly outweigh*  undesirable consequences in most settings  ☐ |

| **Type of recommendation – Prophylactic RBC transfusion volume 10 ml/kg versus >10 ml/kg** | | | |
| --- | --- | --- | --- |
| We recommend against  offering this option  ☐ | We suggest not offering  this option  ☐ | **We suggest offering**  **this option**  ■ | We recommend offering  this option  ☐ |
| Recommendation (text) | We suggest offering a transfusion volume of 10 ml/kg in neonates with cancer (Weak recommendation). | | |
| Justification | Based on limited evidence there is a suggestion that a RBC transfusion volume of 10 ml/kg in comparison to a RBC transfusion volume of >10 ml/kg does not lead to more morbidity (Paul, 2002). However, other outcomes were not included. Moreover, there are no studies included that report any potential benefit from a RBC transfusion volume >10 ml/kg. In addition, the expert panel decided that a lower transfusion volume leads to less exposure and this option is considered probably acceptable for all stakeholders. Therefore we suggest offering this option. | | |
| Subgroup considerations | No subgroup considerations were formulated. | | |
| Implementation considerations | No implementation considerations were formulated. | | |
| Monitoring and evaluation | Not applicable | | |
| Research priorities | See supplemental materials S9 "Gaps in research". | | |

**Table 3.** Evidence to Decision Framework & Overall conclusions - <15 ml/kg versus >15 ml/kg.

| **Prophylactic RBC transfusion volume 15 ml/kg versus >15 ml/kg** | | | | |
| --- | --- | --- | --- | --- |
|  | **Criteria** | **Judgements** | **Research evidence** | **Additional considerations** |
| **PROBLEM** | Is the problem a priority? | ☐ No  ☐ Probably no  ☐ Uncertain  ☐ Probably yes  ■ **Yes** | RBC transfusions are one of the backbones in the supportive care management of children with oncologic diagnoses and those who are undergoing hematopoietic stem cell transplants (HSCT). Children with cancer may require RBC transfusions due to the underlying oncologic disease or bone marrow suppression as a result of the anti-cancer treatment. This guideline is the first guideline to be established specifically for RBC transfusion volumes in children with cancer. |  |
| **BENEFITS AND HARMS** | What is the overall certainty of this evidence? | ☐ No included studies  **■ Very low**  ☐ Low  ☐ Moderate  ☐ High | Summary of findings:  Mortality - Pediatric oncology: no included studies.  - Pediatric: Wong (2005) and Khodabux (2009) reported no significant differences regarding mortality when comparing a volume of 15 ml/kg with 20 ml/kg, RR 1.00 (95% CI 0.07 - 13.87) and RR 0.94 (95% CI 0.43 - 2.04) respectively.  - Adult: no included studies.  Morbidity  Pediatric oncology: no included studies.  - Pediatric: Wong (2005) and Khodabux (2009) reported no significant differences regarding morbidity when comparing a volume of 15 ml/kg with 20 ml/kg.  - Adult: no included studies. |  |
|  | Is there important uncertainty about how much people value the main outcomes? | ☐ Important uncertainty or variability  ☐ Possibly important uncertainty or variability  ☐ Probably no important uncertainty or variability  ■ **No important uncertainty or variability**  ☐ No known undesirable outcomes |  | The relative importance of all outcomes was unanimously determined. |
|  | Are the desirable anticipated effects large? | ☐ No  ☐ Probably no  ■ **Uncertain**  ☐ Probably yes  ☐ Yes  ☐ Varies |  | The desirable effects are unknown e.g., quality of life, hospital admission, costs. |
|  | Are the undesirable anticipated effects small? | ☐ No  ☐ Probably no  ☐ Uncertain  ■ **Probably yes**  ☐ Yes  ☐ Varies |  | There is no significant difference regarding mortality and morbidity. Thus the undesirable effects are probably small. |
|  | Are the desirable effects large relative to undesirable effects? | ☐ No  ☐ Probably no  ■ **Uncertain**  ☐ Probably yes  ☐ Yes  ☐ Varies |  | Considering that there are probably no large, anticipated effects and the undesirable effects are uncertain. Thus, the desirable effects are uncertain relative to the undesirable effects. |
| **RESOURCE USE** | Are the resources required small? | ☐ No  ☐ Probably no  ☐ Uncertain  ■ **Probably yes**  ☐ Yes  ☐ Varies |  | The guideline panel decided that the resources necessary are probably small. |
|  | Is the incremental cost small relative to the net benefits? | ☐ No  ☐ Probably no  ■ **Uncertain**  ☐ Probably yes  ☐ Yes  ☐ Varies |  | There are costs involved (e.g., hospital admission costs). However, benefits are considered uncertain. |
| **EQUITY** | What would be the impact on health inequities? | ☐ Increased  ☐ Probably increased  ☐ Uncertain  ☐ Probably reduced  ☐ Reduced  ■ **Varies** |  | The panel expected that implementing this option has no effect on health inequities, given the structure of the Dutch healthcare system. However, considering the different healthcare structures in the world the impact may vary per country. |
| **ACCEPTABILITY** | Is the option acceptable to key stakeholders? | ☐ No  ☐ Probably no  ☐ Uncertain  ■ **Probably yes**  ☐ Yes  ☐ Varies |  | The panel considered the option of a transfusion volume of 15 ml/kg probably acceptable for the key stakeholders, e.g., doctors and parents. |
| **FEASIBILITY** | Is the option feasible to implement? | ☐ No  ☐ Probably no  ☐ Uncertain  ☐ Probably yes  ■ **Yes**  ☐ Varies |  | The panel considered the option of a transfusion volume of 15 ml/kg feasible to implement. |

| **Balance of consequences – Prophylactic RBC transfusion volume 15 ml/kg versus >15 ml/kg** | | | | |
| --- | --- | --- | --- | --- |
| Undesirable consequences  *clearly outweigh*  desirable consequences in most settings  ☐ | Undesirable consequences *probably outweigh*  desirable consequences in most settings  ☐ | **The balance between  desirable and undesirable consequences  *is closely balanced***  ■ | Desirable consequences  *probably outweigh* undesirable consequences in most settings  ☐ | Desirable consequences  *clearly outweigh*  undesirable consequences in most settings  ☐ |

| **Type of recommendation – Prophylactic RBC transfusion volume 15 ml/kg versus >15 ml/kg** | | | |
| --- | --- | --- | --- |
| We recommend against  offering this option  ☐ | We suggest not offering  this option  ☐ | **We suggest offering**  **this option**  ■ | We recommend offering  this option  ☐ |
| Recommendation (text) | We suggest offering a transfusion volume of 15 ml/kg in neonates with cancer (Weak recommendation). | | |
| Justification | Based on limited evidence there is a suggestion that a RBC transfusion volume of 15 ml/kg in comparison to a RBC transfusion volume of >15 ml/kg does not lead to more mortality or morbidity (Khodabux, 2009; Wong 2005). Moreover, there are no studies included that report any potential benefit from a RBC transfusion volume >15 ml/kg. In addition, the expert panel decided that a lower transfusion volume leads to less exposure and this option is considered probably acceptable for all stakeholders. Therefore we suggest offering this option. | | |
| Subgroup considerations | No subgroup considerations were formulated. | | |
| Implementation considerations | No implementation considerations were formulated. | | |
| Monitoring and evaluation | Not applicable | | |
| Research priorities | See supplemental materials S9 "Gaps in research". | | |

**8. INFUSION RATES OF RED BLOOD TRANSFUSIONS**

**8.1 INFUSION RATES OF RED BLOOD TRANSFUSIONS IN CHILDREN WITH CANCER
A. Primary search**No supporting materials.

**B. Additional search**

The full description of the additional guidelines and the AGREE II-scores are presented in Supplemental Materials S5.

**Table 1.** Included additional guidelines.

| **Research question 8A - The effect of prophylactic RBC transfusion at any infusion rate in children with cancer** | |
| --- | --- |
| Recommendations for children | **JPAC (2013): Transfusion Handbook**  *AGREE II assessment: Domain 1 = 94%, Domain 2 = 56%, Domain 3 = 35%, Domain 4 = 56%, Domain 5 = 58%, Domain 6 = 25%, Overall Guideline Assessment: Score 4.*  Recommendations according to the Transfusion Handbook from the JPAC (2013):   - A Hb infusion rate of **5 ml/kg/hour** and the transfusion must be completed within 4 hours.   - Supporting arguments: Based on the British Committee for Standards in Haematology Transfusion Guidelines on the Administration of Blood Components (Robinson, 2017) |

**8.2 INFUSION RATES OF RED BLOOD TRANSFUSIONS IN NEONATES WITH CANCER
A. Primary search**No supporting materials.

**B. Additional search**

The full description of the additional guidelines and the AGREE II-scores are presented in Supplemental Materials S5.

**Table 1.** Included additional guidelines.

| **Research question 8B - The effect of prophylactic RBC transfusion at any transfusion rate in neonates with cancer** | |
| --- | --- |
| Recommendations for neonates | **Federation of Medical Specialists (2019): Blood transfusion policy.**  *AGREE II assessment: Domain 1 = 94%, Domain 2 = 100%, Domain 3 = 71%, Domain 4 = 89%, Domain 5 = 13%, Domain 6 = 100%, Overall Guideline Assessment: Score 6.*  Recommendations according to the Federation of Medical Specialists (2019):   - An infusion rate of **5 ml/kg/hour**   - Supporting arguments: recommendation is based on consensus. |
