# Supplementary material for: Prophylactic red blood cell transfusions in children and neonates with cancer: An evidence-based clinical practice guideline
Source: Support Care Cancer. 2024 Nov 4;32(11):766. doi: 10.1007/s00520-024-08888-3 (PMC11534970; doi:10.1007/s00520-024-08888-3)
Supplement: Supplementary file 10 — Supplementary file10 (DOCX 7 KB) [file 520_2024_8888_MOESM10_ESM.docx]

**Supplemental materials S10: Gaps in research**

During the development of the guideline for erythrocyte transfusions in children and neonates with cancer, a systematic search was created to answer the predetermined research questions. However, only part of these research questions could be answered with the results of this search and it has become abundantly clear that there are still many gaps in the available evidence for RBC transfusions in children and neonates with cancer. The guideline panel, therefore, believes that (follow up) research is not only desirable, but also necessary in order to be able to provide clearer answers to these research questions:

1. We recommend undertaking future (randomized controlled) trials aiming to identify the appropriate hemoglobin (Hb) concentration to guide administration of a RBC transfusion in:
   1. Children with cancer;
   2. Neonates with cancer;
   3. Children with cancer during sepsis;
   4. Neonates with cancer during sepsis;
   5. Children with cancer who undergo radiotherapy;
   6. Neonates with cancer who undergo radiotherapy
   7. Children with cancer with cardiac and pulmonary comorbidities;
   8. Neonates with cancer with cardiac and pulmonary comorbidities;
2. We recommend undertaking future (randomized controlled) trials aiming to guide the management of hyperleukocytosis in children and neonates with cancer.
3. We recommend further investigation in irradiated RBC transfusions aiming to guide the indications for irradiated RBC transfusions in children and neonates with cancer.
4. We recommend undertaking future (randomized controlled) trials aiming to identify the appropriate volume of RBC transfusions in children and neonates with cancer.
5. We recommend undertaking future (randomized controlled) trials aiming to identify the appropriate infusion rate of RBC transfusions in children and neonates with cancer.
6. We recommend undertaking future studies into the incidence of transfusion-related iron toxicity and its clinical consequences in children with an oncological disease considering the hypothesis that the risk for transfusion-related iron toxicity increases when >10 RBC transfusions are given.
