# Supplementary figures and images for: Prophylactic red blood cell transfusions in children and neonates with cancer: An evidence-based clinical practice guideline
Source: Support Care Cancer. 2024 Nov 4;32(11):766. doi: 10.1007/s00520-024-08888-3 (PMC11534970; doi:10.1007/s00520-024-08888-3)

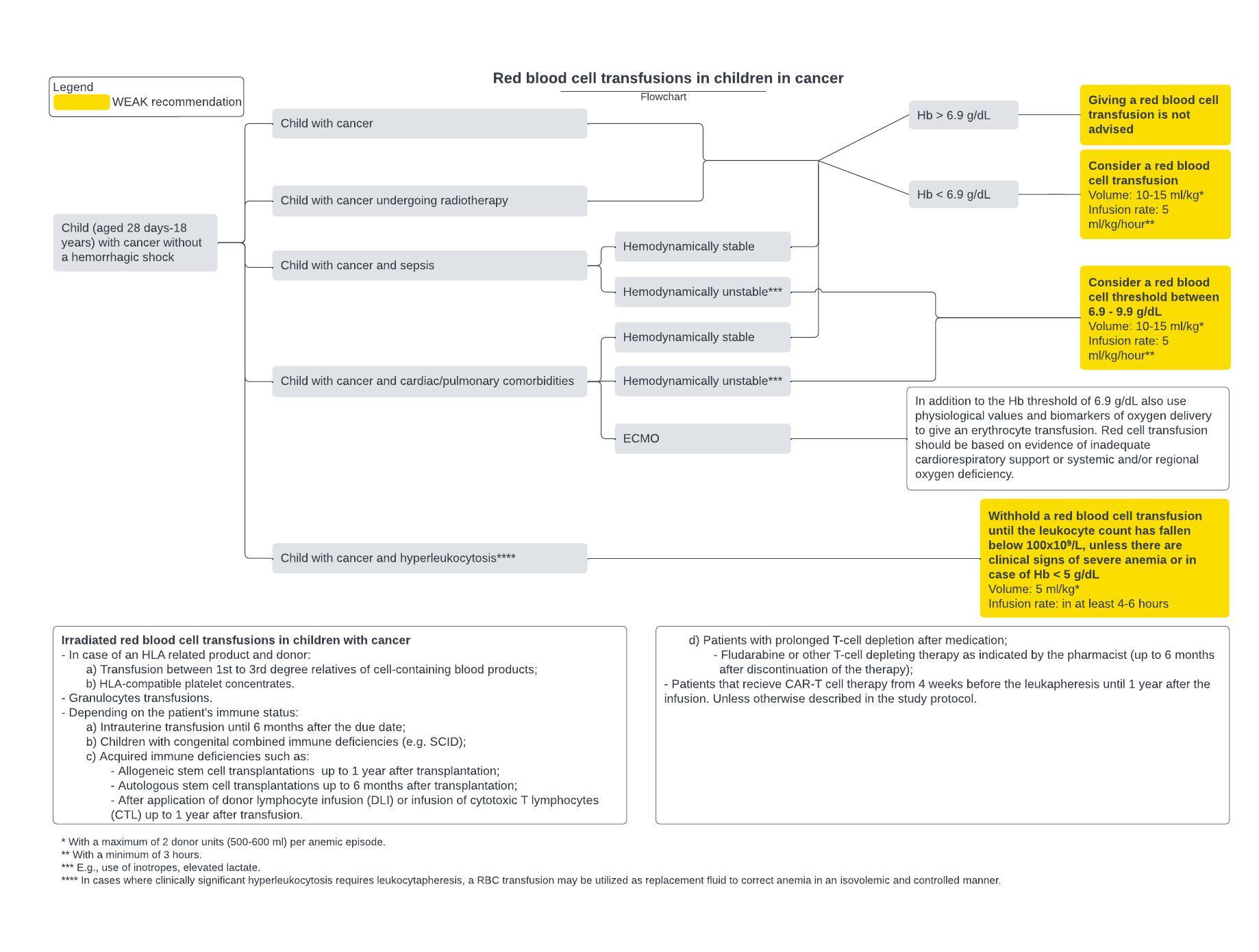


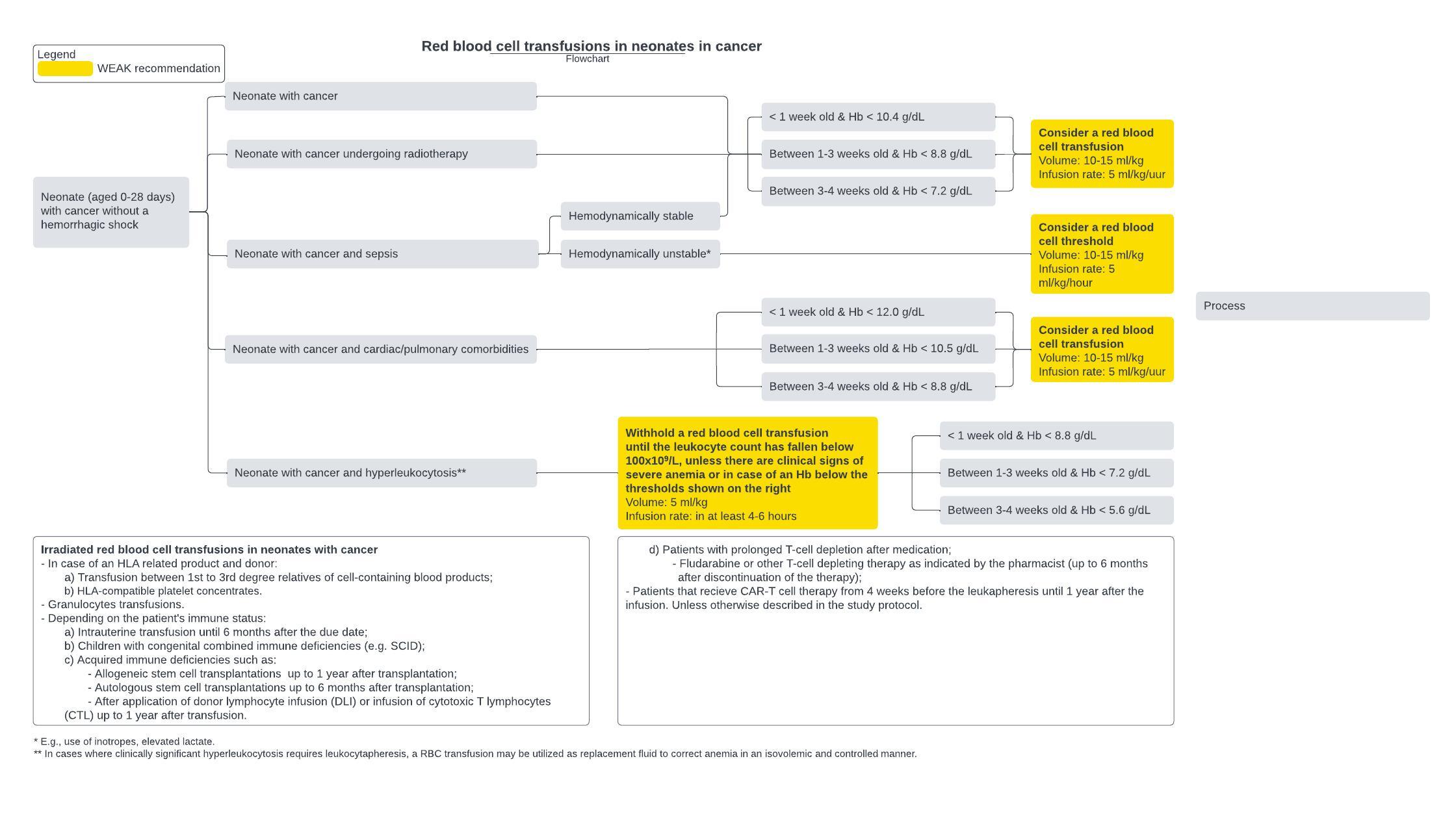

Supplement: Supplementary file 11 — Supplementary file11 (DOCX 455 KB) [file 520_2024_8888_MOESM11_ESM.docx]
